# Dimeric Pimprinine Alkaloids From Soil-Derived *Streptomyces* sp. NEAU-C99

**DOI:** 10.3389/fchem.2020.00095

**Published:** 2020-02-18

**Authors:** Zhiyin Yu, Hao Jiang, Li Wang, Feng-Xian Yang, Jian-Ping Huang, Chongxi Liu, Xiaowei Guo, Wensheng Xiang, Sheng-Xiong Huang

**Affiliations:** ^1^Heilongjiang Provincial Key Laboratory of Agricultural Microbiology, Northeast Agricultural University, Harbin, China; ^2^State Key Laboratory of Phytochemistry and Plant Resources in West China, CAS Center for Excellence in Molecular Plant Sciences, Kunming Institute of Botany, Chinese Academy of Sciences, Kunming, China

**Keywords:** *Streptomyces*, pimprinine, alkaloids, structural characterization, cytotoxicity

## Abstract

Six new pimprinine alkaloids (**1**–**6**), including four dimers, dipimprinines A–D (**1**–**4**), and two monomers, (±)-Pimprinol D (**5**), and pimprinone A (**6**), along with six known congeners (**7**–**12**), were isolated from a soil-derived actinomycete *Streptomyces* sp. NEAU-C99. Structures of the new compounds were elucidated by extensive spectroscopic analyses, single-crystal X-ray diffractions, and ECD calculations. Dipimprinines A–D (**1**–**4**) showed weak cytotoxic activities against five tumor cell lines, including HL-60, SMMC-7721, A-549, MCF-7, and SW-480, with IC_50_ values ranging from 12.7 to 30.7 μM.

## Introduction

Natural products, in particular secondary metabolites derived from actinomycetes, Gram-positive bacteria (Hoshino et al., 2018; Yang et al., 2018), such as antibiotics, enzymes, enzyme inhibitors, and other pharmacologically active agents (Sripreechasak et al., 2013), have contributed substantially to modern medical care (Onaka, 2017). These microbial natural products are still an attractive and indispensable resources for drug discovery due to their potential productivity of unique core skeletons, such as the antiparasitic drug ivermectin (Cragg and Newman, 2013) and the anticancer agent eribulin (Yu et al., 2011). Pimprinine is an indole alkaloid, which was first isolated from the filtrates of *Streptomyces pimprina* cultures in 1963 (Joshi et al., 1963). Members of this family display a range of biological activities, such as antiepileptic (Naik et al., 2001; Roy et al., 2006), platelet-aggregation-inhibitory (Miao et al., 2004), antitumor (Pettit et al., 2002), fungicidal (Zhang et al., 2012), and anti-plant-viral activities (Liu et al., 2019).

**Figure 1:**
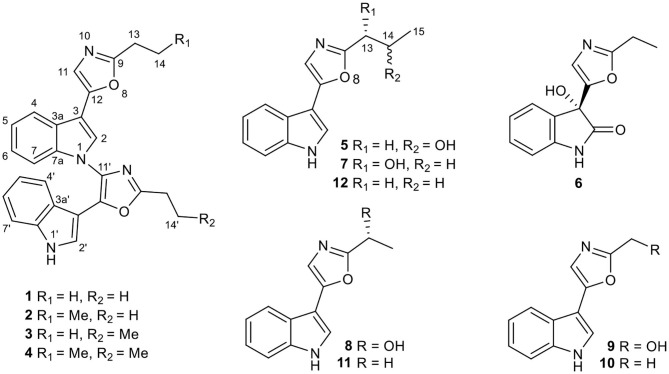
Chemical structures of compounds **1**–**12**.

In the continuation of our chemical and biological screenings of the extracts libraries from endophytes (mainly actinomycetes) in traditional Chinese medicinal (TCM) plants and extremophiles from un- and underexplored ecological niches (Yu et al., 2016; Yang et al., 2017; He et al., 2019), the extract of *Streptomyces* sp. NEAU-C99, isolated from a soil sample collected in Mount Song, Henan province, China, in 2016, indicated distinct UV absorptions compared with the extracts of other strains. As a result, six new pimprinine alkaloids (**1**–**6**), along with six known congeners (**7**–**12**) including pimprinol C (**7**) (Raju et al., 2012), pimprinol A (**8**) (Raju et al., 2012), (5-(1H-indol-3-yl)oxazol-2-yl)methanol (**9**) (Liu et al., 2019), pimprinine (**10**) (Noltemeyer et al., 1982), pimprinethine (**11**) (Pettit et al., 2002), and WS-30581 A (**12**) (Wei et al., 2014), were isolated from *Streptomyces* sp. NEAU-C99 (Figure 1). Herein, we describe the isolation and structure elucidation of six new pimprinine alkaloids analogs (**1–6**), as well as their cytotoxic activities against HL-60, SMMC-7721, A-549, MCF-7, and SW-480 cell lines.

## Materials and Methods

### General Experimental Procedures

NMR spectra were recorded in methanol-*d*_4_ or CDCl_3_ using a Bruker AVANCE III-600 or AVANCE III-400 spectrometer (Bruker Corp., Switzerland), and tetramethylsilane (TMS) was used as internal standard. HRESIMS data were obtained using an Agilent G6230 Q-TOF mass instrument (Agilent Corp., USA) or a Shimadzu UPLC-IT-TOF mass instrument (Shimadzu Corp., Japan). Optical rotation data were determined in MeOH on an Autopol VI S2&Plus polarimeter (Rudolph Research Analytical, Hackettstown, USA). CD spectra were recorded on an Applied Photophysics digital circular dichroism chiroptical spectrometer (Applied Photophysics Limited, Surrey, United Kingdom). IR spectra were measured on a Nicolet™ iS™ 10 FT-IR spectrometer with KBr disks (Thermo Fisher Scientific, Waltham, USA). X-ray crystallographic analysis was carried out with a Bruker APEX DUO single crystal X-ray diffractometer (Bruker Corp., Switzerland). Thin-layer chromatography (TLC) was performed using precoated silica gel GF254 plates (0.25 mm in thickness, Qingdao Marine Chemical Inc., China), and spots were visualized by UV light (254 nm) and colored by spraying heated silica gel plates with 10% H_2_SO_4_ in ethanol. Semipreparative HPLC was conducted on a HITACHI Chromaster system (Hitachi Corp., Japan) equipped with a DAD detector, an YMC-Hydrosphere C_18_ column (250 × 10 mm i.d., 5 μm) at a flow rate of 3.0 mL/min and a column temperature of 25°C.

### Bacterial Strains

The strain *Streptomyces* sp. NEAU-C99 was isolated from a soil sample collected in Mount Song, Henan Province, China, in 2016. It was identified as *Streptomyces* sp. on the basis of the morphological characteristics and 16S *rRNA* gene sequence (GenBank: MN647558) with closest homology to that of *Streptomyces netropsis* strain SXYM16 (100% similarity, GenBank: JN999913.1).

### Fermentation, Extraction, and Isolation

The strain *Streptomyces* sp. NEAU-C99 was grown on ISP3 agar plates (Oatmeal 20 g, KNO_3_ 0.2 g, MgSO_4_·7H_2_O 0.2 g, K_2_HPO_4_·3H_2_O 0.5 g, and Agar 20 g in 1 L of water, pH 7.2) for 7 days at 28°C. Then it was inoculated into 250 mL baffled erlenmeyer flasks containing 50 mL of sterile seed medium (Tryptone Soy Broth, 30 g/L) and cultivated for 2 days at 30°C on a rotary shaker (200 rpm). After that, aliquots (12.5 mL) of the seed culture were transferred into 1,000 mL baffled Erlenmeyer flasks filled with 250 mL of production medium consisting of 2% soluble starch (w/v), 2% tryptone (w/v), 1% glycerol (w/v), 0.05% NaCl (w/v), 0.05% K_2_HPO_4_·3H_2_O (w/v), 0.05% MgSO_4_·7H_2_O (w/v), 0.05% FeSO_4_·7H_2_O (w/v), and 0.1% KNO_3_ (w/v), and cultured on a rotary shaker (200 rpm) at 30°C for a week.

The fermentation broth (25 L) was centrifuged (4,000 rpm, 20 min), and the supernatant was extracted with EtOAc for three times. The EtOAc extract was subsequently evaporated in vacuo to afford 10.0 g of oily crude extract. The mycelia were extracted with methanol (1 L × 3) and then concentrated in vacuo to remove the methanol to yield the aqueous concentrate. This aqueous concentrate was finally extracted with EtOAc (1 L × 3) to give 1.0 g of oily crude extract after removing the EtOAc. Both extracts revealed an identical set of metabolites based on HPLC and TLC analyses, and therefore, they were combined for further purification.

The crude extract in total (11.0 g) was subjected to silica gel column chromatography (CC) using a successive elution of petroleum ether/EtOAc (1:0, 10:1, 5:1, 3:1, 1:1, and 0:1, v/v) to yield fractions A–F. Fr.A (petroleum ether/EtOAc, 10:1, v/v) was subjected to semipreparative HPLC (0–20.0 min, 45% CH_3_CN in H_2_O; 20.1–48.0 min, 69% CH_3_CN in H_2_O; 48.1–52.0 min, 100% CH_3_CN) directly to afford compounds **1** (*t*_R_ = 36.4 min, 2.2 mg), **2** (*t*_R_ = 41.4 min, 2.6 mg), **3** (*t*_R_ = 42.4 min, 2.3 mg), and **4** (*t*_R_ = 49.2 min, 2.0 mg). Fr.B (petroleum ether/EtOAc, 5:1, v/v) was further purified by semipreparative HPLC (0–20.0 min, 45% CH_3_CN in H_2_O; 20.1–48.0 min, 69% CH_3_CN in H_2_O; 48.1–52.0 min, 100% CH_3_CN) to give **10** (*t*_R_ = 17.5 min, 30.7 mg) and **11** (*t*_R_ = 24.3 min, 5.7 mg). Fr.C (petroleum ether/EtOAc, 3:1, v/v) was applied to semipreparative HPLC (0–20.0 min, 40% CH_3_OH in H_2_O; 20.1–35.0 min, 62% CH_3_OH in H_2_O; 35.1–40 min, 100% CH_3_OH) to obtain **12** (*t*_R_ = 35.1 min, 5.0 mg). Compounds **9** (*t*_R_ = 18.9 min,1.4 mg), **8** (*t*_R_ = 25.3 min, 22.6 mg), **5** (*t*_R_ = 32.2 min, 4.8 mg), **6** (*t*_R_ = 33.2 min, 5.7 mg), and **7** (*t*_R_ = 40.0 min, 8.1 mg) were obtained from fraction D (Petroleum ether/EtOAc, 1:1, v/v) by semipreparative HPLC (0–33.0 min, 48% CH_3_OH in H_2_O; 33.1–43.0 min, 56% CH_3_OH in H_2_O; 43.1–58.0 min, 78% CH_3_OH in H_2_O).

### Spectroscopic Characterization of Compounds 1–6

Dipimprinine A (**1**): yellow powder (MeOH), UV (MeOH) λ_max_ (log ε): 227 (4.75), 266 (4.60) nm; IR (KBr) ν_max_ 3,399, 2,962, 2,925, 2,854, 1,644, 1,572, 1,541, 1,461, 1,261, 1,098, 1,016, 802, 743 cm^−1^; ^1^H (600 MHz, CDCl_3_) and ^13^C (150 MHz, CDCl_3_) NMR data (see Table 1); HRESIMS *m/z* 421.1670 [M-H]^−^ (calcd for C_26_H_21_N_4_O_2_, 421.1670).

**Table 1:** ^1^H (600 MHz) and ^13^C (150 MHz) NMR Data of Compounds **1**–**4** in CDCl_3_.

**No**.	**1**		**2**		**3**		**4**	
	**δ_C_**	**δ_H_ (*J* in Hz)**	**δ_C_**	**δ_H_ (*J* in Hz)**	**δ_C_**	**δ_H_ (*J* in Hz)**	**δ_C_**	**δ_H_ (*J* in Hz)**
2	125.0	7.64, s	125.0	7.63, s	125.0	7.64, s	125.0	7.63, s
3	107.3		107.5		107.5		107.5	
3a	124.9		125.1		125.1		125.1	
4	120.2	7.88, d (7.9)	120.3	7.88, d (7.9)	120.3	7.88, d (7.9)	120.3	7.88, d (7.9)
5	121.5	7.27, m	121.7	7.27, m	121.7	7.27, m	121.7	7.27, m
6	123.5	7.22, m	123.6	7.21, m	123.6	7.21, m	123.6	7.22, m
7	111.9	7.25, d (7.1)	112.1	7.24, d (7.6)	112.1	7.24, d (7.6)	112.1	7.23, d (6.8)
7a	136.4		136.5		136.5		136.5	
9	164.0		163.2		164.1		163.2	
11	120.2	7.20, s	120.4	7.19, s	120.4	7.19, s	120.5	7.20, s
12	146.8		146.9		146.9		146.8	
13	21.7	2.87, q (7.6)	30.2	2.81, t (7.5)	21.9	2.87, q (7.6)	30.3	2.81, t (7.5)
14	11.2	1.40, t (7.6)	20.8	1.86, m	11.4	1.40, t (7.6)	20.7	1.86, m
15			13.9	1.03, t (7.4)			13.9	1.04, t (7.4)
2′	122.7	6.75, d (2.7)	122.9	6.74, d (2.7)	122.8	6.74, d (2.7)	122.9	6.74, d (2.6)
3′	103.9		104.0		104.0		104.0	
3a′	124.4		124.6		124.6		124.6	
4′	120.7	7.93, d (8.0)	120.8	7.93, d (8.0)	120.8	7.93, d (8.0)	120.8	7.93, d (8.0)
5′	121.2	7.21, m	121.3	7.20, m	121.3	7.20, m	121.3	7.21, m
6′	123.2	7.26, m	123.4	7.26, m	123.4	7.26, m	123.4	7.26, m
7′	111.4	7.38, d (8.1)	111.6	7.37, d (8.1)	111.6	7.37, d (8.1)	111.6	7.37, d (8.1)
7a′	135.6		135.8		135.8		135.8	
9′	162.5		162.7		161.8		161.8	
11′	128.5		128.6		128.6		128.6	
12′	140.4		140.6		140.5		140.5	
13′	22.1	3.00, q (7.6)	22.2	2.99, q (7.6)	30.6	2.94, t (7.5)	30.6	2.94, t (7.5)
14′	11.3	1.51, t (7.6)	11.3	1.51, t (7.6)	20.7	1.98, m	20.8	1.98, m
15′					14.0	1.13, t (7.4)	14.0	1.13, t (7.4)
NH		8.23, br s		8.32, br s		8.26, br s		8.25, br s

Dipimprinine B (**2**): yellow powder (MeOH), UV (MeOH) λ_max_ (log ε): 224 (4.56), 266 (4.32) nm; IR (KBr) ν_max_ 3,411, 2,963, 2,930, 2,874, 1,642, 1,572, 1,542, 1,463, 1,236, 1,193, 1,128, 1,014, 8,01, 743 cm^−1^; ^1^H (600 MHz, CDCl_3_) and ^13^C (150 MHz, CDCl_3_) NMR data (see Table 1); HRESIMS *m/z* 435.1839 [M-H]^−^ (calcd for C_27_H_23_N_4_O_2_, 435.1826).

Dipimprinine C (**3**): yellow powder (MeOH), UV (MeOH) λ_max_ (log ε*)*: 224 (4.56), 266 (4.31) nm; IR (KBr) ν_max_ 3,403, 2,962, 2,927, 1,643, 1,572, 1,541, 1,462, 1,261, 1,193, 1,127, 1,099, 1,013, 803, 743 cm^−1^; ^1^H (600 MHz, CDCl_3_) and ^13^C (150 MHz, CDCl_3_) NMR data (see Table 1); HRESIMS *m/z* 435.1840 [M-H]^−^ (calcd for C_27_H_23_N_4_O_2_, 435.1826).

Dipimprinine D (**4**): yellow powder (MeOH), UV (MeOH) λ_max_ (log ε): 224 (4.74), 266 (4.52) nm; IR (KBr) ν_max_ 3,412, 2,962, 2,929, 2,873, 1,641, 1,571, 1,542, 1,462, 1,260, 1,192, 1,097, 1,013, 803, 742 cm^−1^; ^1^H (600 MHz, CDCl_3_) and ^13^C (150 MHz, CDCl_3_) NMR data (see Table 1); HRESIMS *m/z* 449.1991 [M-H]^−^ (calcd for C_28_H_25_N_4_O_2_, 449.1983).

(±)-Pimprinol D (**5**): white block crystals (CHCl_3_:MeOH:H_2_O 10:5:1), UV (MeOH) λ_max_ (log ε): 225 (4.40), 267 (4.26) nm; IR (KBr) ν_max_ 3,244, 2,968, 1,638, 1,581, 1,442, 1,354, 1,247, 1,133, 1,120, 1,079, 733 cm^−1^; ^1^H (600 MHz, CD_3_OD) and ^13^C (150 MHz, CD_3_OD) NMR data (see Table 2); HRESIMS *m/z* 243.1128 [M+H]^+^ (calcd for C_14_H_15_N_2_O_2_, 243.1128).

**Table 2:** ^1^H (600 MHz) and ^13^C (150 MHz) NMR Data of Compounds **5** and **6** in CD_3_OD.

**No**.	**5**		**6**	
	**δ_C_**	**δ_H_ (*J* in Hz)**	**δ_C_**	**δ_H_ (*J* in Hz)**
2	123.8	7.61, s	178.0	
3	105.5		73.9	
3a	125.3		130.6	
4	120.5	7.80, d (7.8)	126.3	7.44, d (7.4)
5	121.3	7.15, m	124.0	7.09, t (7.4)
6	123.4	7.20, m	131.5	7.32, td (7.7, 0.9)
7	112.8	7.43, d (7.9)	111.6	6.94, d (7.8)
7a	138.2		142.9	
9	161.8		168.7	
11	119.3	7.19, s	125.5	6.88, s
12	150.2		151.2	
13	38.6	2.96, m	22.4	2.77, q (7.6)
14	66.9	4.28, m	11.3	1.28, t (7.6)
15	23.3	1.29, d (6.2)		

Crystal data for **5**: C_14_H_14_N_2_O_2_, M = 242.27, *a* = 16.7789(4) Å, *b* = 7.4526(2) Å, *c* = 19.6259(4) Å, α = 90°, β = 90°, γ = 90°, *V* = 2454.15(10) Å^3^, *T* = 100. (2) K, space group *Pbca, Z* = 8, μ(Cu Kα) = 0.724 mm^−1^, 25,660 reflections measured, 2,428 independent reflections (*R*_*int*_ = 0.0470). The final *R*_1_ values were 0.0566 [*I* > 2σ(*I*)]. The final *wR*(*F*^2^) values were 0.1354 [*I* > 2σ(*I*)]. The final *R*_1_ values were 0.0578 (all data). The final w*R* (*F*^2^) values were 0.1362 (all data). The goodness of fit on *F*^2^ was 1.126. Original crystallographic data of **5** has been deposited in the Cambridge Crystallographic Data Center (CCDC), with deposition number of CCDC1964253. Copies of the data can be obtained from the website of CCDC free of charge.

Pimprinone A (**6**): yellow oil (MeOH), ${\left[\alpha \right]}_{\text{D}}^{26.0}$−20.92 (*c* 0.1, MeOH); UV (MeOH) λ_max_ (log ε): 209 (4.78), 215 (4.76), 294 (3.49) nm; ECD (MeOH) λ(ε) 292 (−0.21), 278 (−0.04), 265 (−0.18), 227 (+1.06), 207 (−1.02); IR (KBr) ν_max_ 3,212, 2,984, 1,728, 1,621, 1,561, 1,473, 1,384, 1,327, 1,225, 1,185, 1,110, 1,062, 1,001, 911, 756, 689 cm^−1^; ^1^H (600 MHz, CD_3_OD) and ^13^C (150 MHz, CD_3_OD) NMR data (see Table 2); HRESIMS *m/z* 267.0731 [M+Na]^+^ (calcd for C_13_H_12_N_2_O_3_Na, 267.0740).

### Cytotoxicity Assay

Five tested human tumor cell lines, human leukemia (HL-60), hepatocellular carcinoma (SMMC-7721), lung cancer (A-549), breast adenocarcinoma (MCF-7), and colon carcinoma (SW-480), were purchased from ATCC (Manassas, VA, USA). Each of these cell lines was incubated in medium DMEM or RPMI-1640 containing 10% fetal bovine serum at 37°C under humidified atmosphere with 5% CO_2_. Cytotoxicity of the isolates toward these tumor cell lines was assessed via the 3-(4, 5-dimethylthiazol-2-yl)-5(3-carboxymethoxyphenyl)-2-(4-sulfopheny)-2H tetrazolium (MTS) (Promega, Madison, WI, USA) method (Cory et al., 1991), and cisplatin (Sigma) was used as a positive control. The cell lines were inoculated into each well of the normal 96-well plates and incubated for 12 h before addition of the test isolates. Different concentrations of each compound were added and exposed to the cells for a continuous cultivation of 48 h. The isolates with inhibition rates ≥50% against the cell lines were further assessed in triplicate at different concentrations (0.064, 0.32, 1.6, 8, and 40 μM). The IC_50_ values were measured based on Reed and Muench's method (Reed and Muench, 1938). All the experiments were carried out in triplicate.

## Results and Discussion

Compound **1** was obtained as yellow amorphous powder, and its molecular formula C_26_H_22_N_4_O_2_ was determined by high resolution electrospray ionization mass spectrometry (HRESIMS) data (*m/z* 421.1670 [M-H]^−^, calcd for 421.1670), corresponding to 18 degrees of unsaturation (Figure S8). The ^1^H NMR and ^1^H-^1^H COSY data (Table 1, Figures S3, S5) indicated a 1,3-substituted indole ring with signals at δ_H_ 7.88 (1H, d, *J* = 7.9 Hz, H-4), 7.64 (1H, s, H-2), 7.27(m, H-5), 7.25 (1H, d, *J* = 7.1 Hz, H-7), and 7.22 (m, H-6) and a 3-substituted indole ring with signals at δ_H_ 7.93 (1H, d, *J* = 8.0 Hz, H-4′), 7.38 (1H, d, *J* = 8.1 Hz, H-7′), 7.26 (m, H-6′), 7.21 (m, H-5′), and 6.75 (1H, d, *J* = 2.7 Hz, H-2′), along with the active amine-hydrogen signal (δ_H_ 8.23, H-1′). The ^13^C and DEPT spectra of **1** suggested the presence of 26 carbons, which were classified into two methyls, two methylenes, 11 aromatic nonprotonated carbons, and 11 aromatic methine carbons (Table 1, Figure S4). These signals appeared in pairs in the ^13^C NMR spectrum, which were very similar to those of pimprinethine (Pettit et al., 2002). The aforementioned spectroscopic evidences suggested that compound **1** was likely a dimeric pimprinine alkaloid.

**Figure 2:**
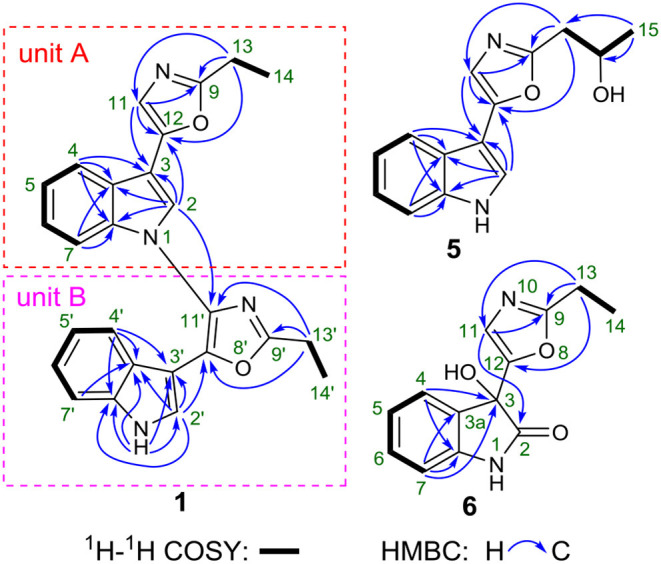
2D NMR correlations of **1**, **5**, and **6**.

In unit A, the ^1^H–^1^H COSY and HSQC spectra of **1** showed two spin-coupling systems, H-14/H-13 and H-4/H-5/H-6/H-7 (Figure 2, Figures S5, S6). The HMBC cross-peaks from H-2 and H-4 to C-3/C-3a/C-7a, from H-7 to C3a/C-7a, further revealed the presence of an indole moiety. The HMBC cross-peaks from H-11 to C-9/C-12/C-3, from H_2_-13 to C-9/C-11/C-12, and from H-2 to C-12 were observed in the HMBC spectrum (Figure 2, Figure S7), which suggested a 2-ethyl-oxazole was connected to C-3 of an indole moiety. The above data resembled those of pimprinethine. Similarly, unit B in **1** was constructed by the following signals, correlations of H-4′ to H-7′, NH-1′/H-2′, and H_2_-13′/H_3_-14′ observed in the ^1^H–^1^H COSY spectrum, and cross-peaks of H-4′ with C-3′/C-3a′/C-7a′, H-7′ with C-3a′, NH-1′ with C-2′/C-3′/C-3a′/C-7a′, H-2′ with C-3′/C-3a′/C-7a′/C-12′, and H_2_-13′ with C-9′/C-11′/C-12′ observed in the HMBC spectrum. Units A and B were finally established as being bridged via the N-1–C-11′ bond based on the key HMBC correlation from H-2 to C-11′ (Figure 2). Thus, the structure of compound **1** was determined as shown in Figure 1, and named as dipimprinine A.

Dipimprinine B (**2**) has a molecular formula of C_27_H_24_N_4_O_2_ as established by HRESIMS (*m/z* 435.1839 [M-H]^−^, calcd for 435.1826), which showed 14 mass units more than that of compound **1** (Figure S16). The ^1^H and ^13^C NMR spectra (Table 1, Figures S11, S12) of **2** showed high similarity to those of compound **1**, except for the presence of an additional methylene at δ_C_ 30.2 (C-13) and δ_H_ 2.81 (2H, t, *J* = 7.5 Hz, H-13), suggesting that compound **2** was a derivative of **1**. This deduction was further confirmed by the ^1^H–^1^H COSY coupling fragment of H_2_-13/H_2_-14/H_3_-15 and HMBC correlations from H_3_-15 to C-14/C-13 (Figures S2, S13, S15). Consequently, the structure of compound **2** was thus elucidated as shown (Figure 1).

Dipimprinine C (**3**) shared the same molecular formula C_28_H_26_N_4_O_2_ with **2** as determined by the HRESIMS ion peak at *m/z* 435.1840 [M-H]^−^ (calcd for 435.1826) (Figure S24), suggesting **3** is an isomer of **2**. Indeed, the ^1^H and ^13^C-NMR chemical shifts of **3** were almost the same as those of **2** (Table 1, Figures S19, S20), but differed in the ^1^H-NMR splitting pattern of the proton signals at δ_H_ 2.99 and δ_H_ 2.81, the signal at δ_H_ 2.99 was a quartet in **2** but a triplet in **3**, while the other signal at δ_H_ 2.81 was a triplet in **2** but a quartet in **3**. Based on the in-depth interpretation of its 1D NMR data (Table 1) and 2D NMR data (Figures S21–S23), particularly the ^1^H–^1^H COSY and HMBC correlations, **3** was further revealed as a structural analog of **2** with the obvious HMBC correlations from H_2_-13 to C-14 and from H_2_-13′ to C-14′/C-15′ and the ^1^H–^1^H COSY cross-peaks of H_2_-13/H_3_-14 and H_2_-13′/H_2_-14′/H_3_-15′ (Figure S2). Therefore, the structure of compound **3** was identified as shown in Figure 1.

**Figure 3:**
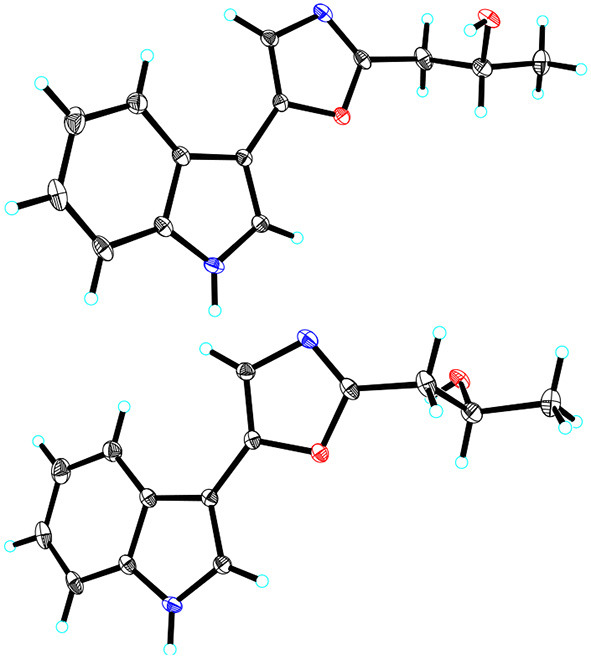
X–ray crystallographic structures of **5**.

Dipimprinine D (**4**) was isolated as yellow powder and its molecular formula was assigned as C_28_H_26_N_4_O_2_ based on HRESIMS analysis (*m/z* 449.1991 [M-H]^−^, calcd for 449.1983), with 18 degrees of unsaturation (Figure S32). Its ^1^H and ^13^C NMR data closely resembled those of **1**, apart from two additional sp^3^ methylene resonances at δ_H_ 1.86 (2H, m, H-14), δ_C_ 20.7 (C-14) and δ_H_ 1.98 (m, 2H, H-14′), δ_C_ 20.8 (C-14′) (Table 1, Figures S27, S28). It can be inferred that the two ethyl moieties at C-9 and C-9′ in **1** were replaced by two propyl groups in **4**, which was further supported by ^1^H–^1^H COSY cross-peaks of H_2_-13/H_2_-14/H_3_-15, and H_2_-13′/H_2_-14′/H_3_-15′ (Figures S2, S29). Hence, the structure of compound **4** was established.

(±)-Pimprinol D (**5**) possesses a molecular formula of C_14_H_14_N_2_O_2_ from its HRESIMS data (*m/z* 243.1128 [M+H]^+^, calcd for 243.1128) (Figure S40). The ^13^C NMR spectrum of **5** showed a total of 14 carbon resonances (Table 2). Detailed analyses of its 1D NMR and HSQC data enabled the classification of these carbons as one methyl, one methylene, one sp^3^ methine, six sp^2^ methines and five sp^2^ quaternary carbons (Figures S35, S36, S38). The ^1^H and ^13^C NMR spectra of **5** had similar features to those of pimprinol C (**7**) (Raju et al., 2012). The major difference was that the C-14 was replaced by a hydroxy group in **5**, which was confirmed by the ^1^H–^1^H COSY correlations of H_2_-13/H-14/H_3_-15 (Figure 2, Figure S37). To assign the absolute configuration of **5**, its X-ray diffraction data was obtained using Cu Kα radiation. Its X-ray crystallographic data showed a space group of *Pbca* (Yesilyurt et al., 2018; Cai et al., 2019). Detailed X-ray crystallographic analysis showed that compound **5** was a racemate, and the indole ring and the oxazole ring were almost coplanar in **5** (Figure 3). Hence, the structure of **5** was elucidated as shown in Figure 1, and it was named as (±)-Pimprinol D (**5**).

**Figure 4:**
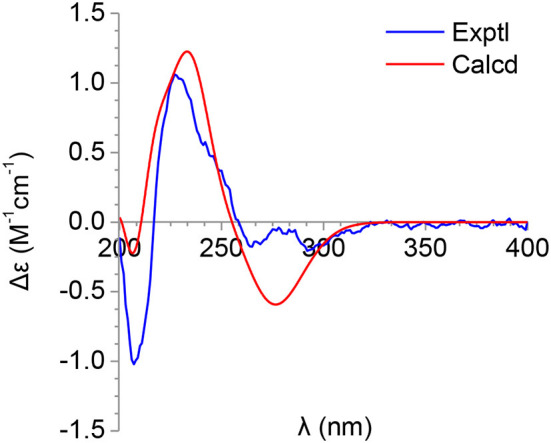
Calculated and experimental ECDs of **6**.

The molecular formula of pimprinone A (**6**) was determined as C_13_H_12_N_2_O_3_ on the basis of HRESIMS (*m/z* 267.0731 [M+Na]^+^, calcd for 267.0740), accounting for nine degrees of unsaturation (Figure S47). The ^1^H NMR spectrum exhibited nine signals, including one methyl signals at δ_H_ 1.28 (3H, t, *J* = 7.6 Hz, H-14), one methylene signal at δ_H_ 2.77 (2H, q, *J* = 7.6 Hz, H-13), one single olefin proton signal at δ_H_ 6.88 (s, H-11), and four mutually coupled signals of aryl protons at δ_H_ 7.44 (1H, d, *J* = 7.4 Hz, H-4), 7.32 (1H, td, *J* = 7.7, 0.9 Hz, H-6), 7.09 (1H, t, *J* = 7.4 Hz, H-5), and 6.94 (1H, d, *J* = 7.8 Hz, H-7), indicating the presence of an *ortho*-disubstituted benzene (Table 2, Figure S42). The ^13^C NMR spectrum revealed a carbonyl carbon (δ_C_ 178.0), six aromatic carbons (δ_C_ 142.9, 131.5, 130.6, 126.3, 124.0, 111.6), and an oxygenated tertiary carbon (δ_C_ 73.9) (Figure S43). These spectroscopic data implied the presence of 3-hydroxy-oxindole (Park et al., 2018) moiety in **6**. The ^1^H and ^13^C NMR data of **6** were similar to those of pimprinethine (**11**) (Pettit et al., 2002), except that the olefinic bond at C-2/C-3 was substituted by a carbonyl (δ_C_ 178.0, C-2) and a sp^3^ non-protonated carbon (δ_C_ 73.9, C-3). The assumption was confirmed by the HMBC correlations from H-7 (δ_H_ 6.94) to C-3 (δ_C_ 73.9), from H-4 (δ_H_ 7.44) to C-3 (δ_C_ 73.9), and from H-11 (δ_H_ 6.88) to C-2 (δ_C_ 178.0) (Figure 2), and evidenced from the molecular formula, respectively. Analysis of the 2D NMR data confirmed that the other parts of **6** were the same as those of pimprinethine (**11**) (Figures S44–S46). Therefore, the planar structure of **6** was elucidated as depicted in Figure 1. To confirm the absolute configuration of **6**, we then performed electronic circular dichroism (ECD) calculations of (3*R*)-**6** using time-dependent density functional theory (TDDFT) (Supplementary Material, p. S5). The calculated ECD spectrum of **6** was in good agreement with the experimental one (Figure 4). Ultimately, the absolute configuration of the only chiral carbon C-3 in **6** was identified as *R*.

As mentioned above, the stereochemistry of rings in the monomeric pimprinines turns out to be planar according to the result of X-ray crystallographic analysis (Figure 3). To investigate the possible potential axial chirality in dimeric pimprinine molecules, CD spectra for dipimprinines A–D (**1**–**4**) were acquired (Figures S10, S18, S26, S34). Unlike the reported natural dimeric atropisomers (Wang et al., 2013; Tshitenge et al., 2019), no Cotton effects can be found in any CD spectra of dipimprinines A–D (**1**–**4**). Consequently, either dipimprinines A–D (**1**–**4**) have no atropisomeric stereochemistry (that's to say a plane structure) or they were all racemates. Actually, it's more likely that compounds **1**–**4** have no atropisomeric stereochemistry. The carbon-nitrogen bond (N-1–C-11′) in compounds **1**–**4** can rotate in a circle without any steric hindrance for the reason that no substituents can be found at neither C-2 nor N-10′.

All the new compounds were evaluated for their cytotoxic activities against five human tumor cell lines, human leukemia (HL-60), hepatocellular carcinoma (SMMC-7721), lung cancer (A-549), breast adenocarcinoma (MCF-7), and colon carcinoma (SW-480), and cisplatin was used as a positive control. As shown in Table 3, compounds **1**, **2**, and **4** showed antiproliferative activity against breast adenocarcinoma cell line MCF-7 with IC_50_ values ranging from 13.8 to 18.2 μM, while the same treatment on cisplatin turned out to be an IC_50_ value of 26.8 μM. Compound **3** showed weak inhibitory activity against hepatocellular carcinoma cell line SMMC-7721 with an IC_50_ value of 25.2 μM.

**Table 3:** Cytotoxicity of compounds **1**–**4** against five human tumor cell lines^a^.

**Compounds**	**HL-60**	**A-549**	**SMMC-7721**	**MCF-7**	**SW-480**
1	29.65 ± 1.10	29.90 ± 0.51	12.68 ± 0.49	18.20 ± 0.83	27.70 ± 1.15
2	NA	17.36 ± 0.12	14.69 ± 0.48	15.68 ± 0.74	30.67 ± 1.51
3	NA	NA	25.19 ± 1.65	NA	NA
4	20.90 ± 0.28	22.88 ± 0.21	13.68 ± 0.64	13.75 ± 0.61	29.54 ± 1.76
Cisplatin^b^	2.79 ± 0.31	16.00 ± 0.69	5.98 ± 0.19	26.79 ± 0.77	25.43 ± 0.89

a *Results are expressed as IC_50_ ± SD values in μM*.

b *Positive control*.

*NA, not active*.

## Conclusions

In summary, this work describes the isolation and characterization of six new pimprinine alkaloids (**1**–**6**) from a soil-derived actinomycete *Streptomyces* sp. NEAU-C99. Their structures including absolute configurations were determined by extensive spectroscopic data, single-crystal X-ray diffraction analysis, and ECD calculations. Cytotoxicity assays showed that compounds **1**, **2**, and **4** displayed moderate antitumor activity against breast adenocarcinoma MCF-7. Compounds **1**–**4** were represented as the first examples of dimeric pimprinine alkaloids, which could further enrich the structure diversities of pimprinine alkaloids.

## Data Availability Statement

All datasets for this study are included in the article/Supplementary Material.

## Author Contributions

ZY performed the experiments, identified the structures, and prepared the original manuscript. HJ isolated and identified the strain, and conducted the cytotoxicity assay. LW collected the spectrographic data, assisted with the structure elucidation and manuscript revision. F-XY, J-PH, CL and XG revised the manuscript. WX and S-XH designed and supervised the research and revised the manuscript.

### Conflict of Interest

The authors declare that the research was conducted in the absence of any commercial or financial relationships that could be construed as a potential conflict of interest.

## Supplementary Material

The Supplementary Material for this article can be found online at: https://www.frontiersin.org/articles/10.3389/fchem.2020.00095/full#supplementary-material HRESIMS, IR, CD, 1D and 2D NMR spectra of compounds **1**–**6**, detailed ECD calculations of compound **6**, as well as the NMR data of compounds **7**–**12** (PDF).
