# Corrigendum: Dimeric Pimprinine Alkaloids From Soil-Derived *Streptomyces* sp. NEAU-C99

**DOI:** 10.3389/fchem.2020.00717

**Published:** 2020-08-19

**Authors:** Zhiyin Yu, Hao Jiang, Li Wang, Feng-Xian Yang, Jian-Ping Huang, Chongxi Liu, Xiaowei Guo, Wensheng Xiang, Sheng-Xiong Huang

**Affiliations:** ^1^Heilongjiang Provincial Key Laboratory of Agricultural Microbiology, Northeast Agricultural University, Harbin, China; ^2^State Key Laboratory of Phytochemistry and Plant Resources in West China, CAS Center for Excellence in Molecular Plant Sciences, Kunming Institute of Botany, Chinese Academy of Sciences, Kunming, China

**Keywords:** *Streptomyces*, pimprinine, alkaloids, structural characterization, cytotoxicity

In the original article, there was a mistake in Figure 1 as published. The structures of compounds 1–4 were drawn wrong. The corrected Figure 1 appears below.

**Figure 1:**
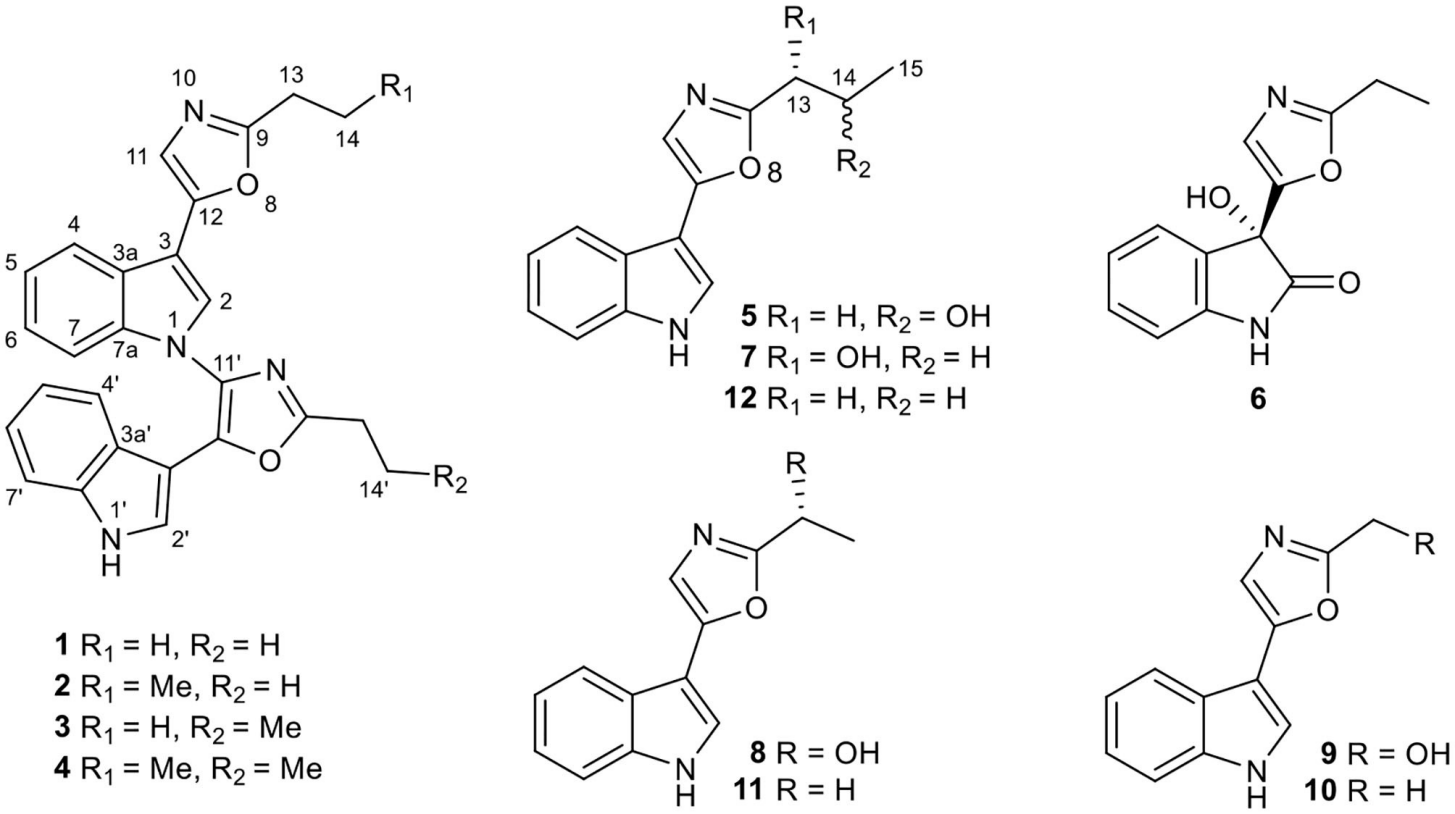
Chemical structures of compounds **1**–**12**.

Additionally, in section Materials and Methods, the first sentence of the “Cytotoxicity Assay” section should be revised as “Five tested human tumor cell lines, human leukemia (HL-60), hepatocellular carcinoma (SMMC-7721), lung cancer (A-549), breast adenocarcinoma (MCF-7), and colon carcinoma (SW-480), were purchased from ATCC (Manassas, VA, USA).”

The authors apologize for these errors and state that this does not change the scientific conclusions of the article in any way. The original article has been updated.

