# Supplementary material for: Dimeric Pimprinine Alkaloids From Soil-Derived Streptomyces sp. NEAU-C99
Source: Front Chem. 2020 Feb 18;8:95. doi: 10.3389/fchem.2020.00095 (PMC7040024; doi:10.3389/fchem.2020.00095)
Supplement: Supplementary file 1 [file Data_Sheet_1.PDF]

Supplementary Material

Dimeric Pimprinine Alkaloids from Soil Derived *Streptomyces* sp. NEAU-C99

Zhiyin Yu[1,2], Hao Jiang[1], Li Wang[2], Fengxian Yang[2], Jian-Ping Huang[2], Chongxi Liu[1,2], Xiaowei Guo[1,2], Wensheng Xiang[1,*] and Sheng-Xiong Huang[2,*]

[1] Heilongjiang Provincial Key Laboratory of Agricultural Microbiology, Northeast Agricultural University, Harbin, China

[2] State Key Laboratory of Phytochemistry and Plant Resources in West China, CAS Center for Excellence in Molecular Plant Sciences, Kunming Institute of Botany, Chinese Academy of Sciences, Kunming, China

*** Correspondence:**
Prof. Dr. Sheng-xiong Huang, E-mail: sxhuang@mail.kib.ac.cn;
Prof. Dr. Wensheng Xiang, E-mail: xiangwensheng@neau.edu.cn

Contents of Supporting Information

NMR data of compounds 7–12: S4

ECD calculation S5

REFERENCES...S6

Table S1. Important thermodynamic parameters (a.u.) and Boltzmann distributions of the optimized compound **6** at B3LYP/6-31G (d, p) level in the gas phase S7

Table S2. Optimized Z-matrixes of compound **6** in the gas phase (Å) at B3LYP/6-31 G (d, p) level S7

Figure S1. Optimized geometries of predominant conformers for compound **6** at the B3LYP/6-31G(d,p) level in the gas phase. S15

Figure S2. 2D NMR correlations of **2**, **3** and **4**. S15

Figure S3. ^{1}H NMR (600 MHz) spectrum of compound **1** in $CDCl_3$. S16

Figure S4. ^{13}C NMR and DEPT (150 MHz) spectrum of compound **1** in $CDCl_3$. ...S17

Figure S5. ^{1}H–^{1}H COSY (600 MHz) spectrum of compound **1** in $CDCl_3$. S18

Figure S6. HSQC (600 MHz) spectrum of compound **1** in $CDCl_3$..........................S19

Figure S7. HMBC (600 MHz) spectrum of compound **1** in $CDCl_3$..........................S20

Figure S8. HRESIMS spectrum of compound **1**. S21

Figure S9. IR spectrum of compound **1**. S22

Figure S10. CD spectrum of compound **1**..S23

Figure S11. ^{1}H NMR (600 MHz) spectrum of compound **2** in $CDCl_3$. S24

Figure S12. ^{13}C NMR (150 MHz) spectrum of compound **2** in CDCl3. ....S25

Figure **S13.** ^{1}H–^{1}H COSY (600 MHz) spectrum of compound **2** in $CDCl_3$. S26

Figure S14. HSQC (600 MHz) spectrum of compound **2** in $CDCl_3$........................S27

Figure S15. HMBC (600 MHz) spectrum of compound **2** in $CDCl_3$........................S28

Figure S16. HRESIMS spectrum of compound **2**. S29

Figure S17. IR spectrum of compound **2**. S30

Figure S18. CD spectrum of compound **2**..S31

Figure S19. ^{1}H NMR (600 MHz) spectrum of compound **3** in $CDCl_3$. S32

Figure S20. ^{13}C NMR (150 MHz) spectrum of compound **3** in $CDCl_3$. S33

Figure S21. ^{1}H–^{1}H COSY (600 MHz) spectrum of compound **3** in $CDCl_3$. S34

Figure S22. HSQC (600 MHz) spectrum of compound **3** in $CDCl_3$........................S35

Figure S23. HMBC (600 MHz) spectrum of compound **3** in $CDCl_3$........................S36
Figure S24. HRESIMS spectrum of compound **3**...S37
Figure S25. IR spectrum of compound **3**. S38
Figure S26. CD spectrum of compound **3**..S39
Figure S27. ^{1}H NMR (600 MHz) spectrum of compound **4** in $CDCl_3$. S40
Figure S28. ^{13}C NMR (150 MHz) spectrum of compound **4** in $CDCl_3$. S41
Figure S29. ^{1}H–^{1}H COSY (600 MHz) spectrum of compound **4** in $CDCl_3$. S42
Figure S30. HSQC (600 MHz) spectrum of compound **4** in $CDCl_3$.........................S43
Figure S31. HMBC (600 MHz) spectrum of compound **4** in $CDCl_3$........................S44
Figure S32. HRESIMS spectrum of compound **4**...S45
Figure S33. IR spectrum of compound **4**. S46
Figure S34. CD spectrum of compound **4**..S47
Figure S35. ^{1}H NMR (600 MHz) spectrum of compound **5** in methanol-d_4. ....S48
Figure S36. ^{13}C NMR (150 MHz) spectrum of compound **5** in methanol-d_4. ....S49
Figure S37. ^{1}H–^{1}H COSY (600 MHz) spectrum of compound **5** in methanol-d_4. ...S50
Figure S38. HSQC (600 MHz) spectrum of compound **5** in methanol-d_4. ....S51
Figure S39. HMBC (600 MHz) spectrum of compound **5** in methanol-d_4..............S52
Figure S40. HRESIMS spectrum of compound **5**...S53
Figure S41. IR spectrum of compound **5** S54
Figure S42. ^{1}H NMR (600 MHz) spectrum of compound **6** in methanol-d_4. ....S55
Figure S43. ^{13}C NMR (150 MHz) spectrum of compound **6** in methanol-d_4. ....S56
Figure S44. ^{1}H–^{1}H COSY (600 MHz) spectrum of compound **6** in methanol-d_4. ...S57
Figure S45. HSQC (600 MHz) spectrum of compound **6** in methanol-d_4. ....S58
Figure S46. HMBC (600 MHz) spectrum of compound **6** in methanol-d_4. ....S59
Figure S47. HRESIMS spectrum of compound **6**...S60
Figure S48. IR spectrum of compound **6**. S61
Figure S49. CD spectrum of compound **6**..S62

NMR data of compounds **7**–**12**:

Pimprinol C (**7**): ^{1}H NMR data (400 MHz, CD_3OD): δ_H7.82 (1H, d, J = 7.7 Hz, H-4), 7.65 (1H, s, H-2), 7.44 (1H, d, J = 7.9 Hz, H-7), 7.23 (1H, s, H-11), 7.17 (2H, m, H-5, H-6), 4.70 (1H, t, J = 6.9 Hz, H-13), 1.98 (2H, m, H-14), 1.01 (3H, t, J = 7.4 Hz, H-15); ^{13}C NMR data (100 MHz, CD_3OD): δ_C 164.5 (C, C-9), 150.4 (C, C-12), 138.1 (C, C-7a), 125.4 (C, C-3a), 124.0 (CH, C-2), 123.5 (CH, C-6), 121.4 (CH, C-5), 120.5 (CH, C-4), 119.1 (CH, C-11), 112.8 (CH, C-7), 105.4 (C, C-3), 69.7 (CH, C-13), 29.6 (CH_2, C-14), 10.1 (CH_3, C-15).

Pimprinol A (**8**): ^{1}H NMR data (400 MHz, CD_3OD): δ_H 7.81 (1H, d, J = 7.7 Hz, H-4), 7.64 (1H, s, H-2), 7.43 (1H, d, J = 7.9 Hz, H-7), 7.21 (1H, s, H-11), 7.18 (1H, m, H-6), 7.14 (1H, m, H-5), 4.95 (1H, q, J = 6.7 Hz, H-13), 1.62 (3H, d, J = 6.6 Hz, H-14); ^{13}C NMR (100 MHz, CD_3OD): δ_C165.0 (C, C-9), 150.4 (C, C-12), 138.1 (C, C-7a), 125.3 (C, C-3a), 124.0 (CH, C-2), 123.5 (CH, C-6), 121.4 (CH, C-5), 120.5 (CH, C-4), 119.2 (CH, C-11), 112.8 (CH, C-7), 105.3 (C, C-3), 64.2 (CH, C-13), 21.5 (CH_3, C-14).

(5-(1H-indol-3-yl)oxazol-2-yl)methanol (**9**): ^{1}H NMR data (400 MHz, CD_3OD): δ_H7.83 (1H, d, J = 7.6 Hz, H-4,), 7.66 (1H, s, H-2), 7.44 (1H, d, J = 7.9 Hz, H-7), 7.25 (1H, s, H-11), 7.20 (1H, m, H-6), 7.17(1H, m, H-5) ,4.69 (2H, s, H-13); ^{13}C NMR data (100 MHz, CD_3OD): δ_C 162.4 (C, C-9), 150.9 (C, C-12), 138.2 (C, C-7a), 125.3 (C, C-3a), 124.1 (CH, C-2), 123.5 (CH, C-6), 121.4 (CH, C-5), 120.5 (CH, C-4), 119.4 (CH, C-11), 112.8 (CH, C-7), 105.3 (C, C-3), 57.5 (CH_2, C-13).

Pimprinine (**10**): ^{1}H NMR data (400 MHz, CD_3OD): δ_H7.78 (1H, d, J = 7.8 Hz, H-4), 7.59 (1H, s, H-2), 7.43 (1H, d, J = 8.0 Hz, H-7), 7.19 (1H, m, H-6), 7.15 (1H, m, H-5) ,7.14 (1H, s, H-11), 2.52 (3H, s, H-13); ^{13}C NMR data (100 MHz, CD_3OD): δ_C160.8 (C, C-9), 150.2 (C, C-12), 138.2 (C, C-7a), 125.3 (C, C-3a), 123.7 (CH, C-2), 123.4 (CH, C-6), 121.3 (CH, C-5), 120.4 (CH, C-4), 119.2 (CH, C-11), 112.8 (CH, C-7), 105.5 (C, C-3), 13.5 (CH_3, C-13).

Pimprinethine (**11**): ^{1}H NMR data (400 MHz, CD_3OD): δ_H7.77 (1H, d, J = 7.8 Hz, H-4), 7.57 (1H, s, H-2), 7.43 (1H, d, J = 7.9 Hz, H-7),7.19 (1H, m, H-6), 7.14 (1H, m, H-5), 7.13 (1H, s, H-11) 2.83 (2H, q, J = 7.6 Hz, H-13), 1.36 (3H, t, J = 7.6 Hz, H-14), ^{13}C NMR data (100 MHz, CD_3OD): δ_C 165.0 (C, C-9), 149.9 (C, C-12), 138.1(C, C-7a), 125.3 (C, C-3a), 123.7 (CH, C-2), 123.4 (CH, C-6), 121.3 (CH, C-5), 120.4 (CH, C-4), 119.1 (CH, C-11), 112.8 (CH, C-7), 105.5 (C, C-3), 22.3 (CH_2, C-13), 11.6 (CH_3, C-14).

WS-30581 A (**12**): ^{1}H NMR data (400 MHz, CD_3OD): δ_H7.78 (1H, d, J = 7.8 Hz, H-4),7.60 (1H, s, H-2), 7.43 (1H, d, J = 7.9 Hz, H-7), 7.19 (1H, m, H-6), 7.16 (1H, s, H-11), 7.15 (1H, m, H-5), 2.82 (2H, t, J = 7.4 Hz, H-13), 1.86 (2H, m, H-14), 1.04 (3H, t, J = 7.4 Hz, H-15).

ECD calculation

The theoretical calculations of compound **6** were performed using Gaussian 09 (Frisch et al., 2010) and figured using GaussView 5.0 (Dennington et al, 2009). Conformation search using molecular mechanics calculations was performed in Discovery Studio 3.5 Client with MMFF force field with 20 kcal mol^{-1} upper energy limit (Smith et al., 2010).

The optimized conformation geometries and thermodynamic parameters of all selected conformations were provided. The predominant conformers were optimized at B3LYP/6-31G (d, p) level. The theoretical calculation of ECD was performed using time dependent Density Functional Theory (TDDFT) at B3LYP/6-31G (d, p) level in MeOH with PCM model (Miertuš et al., 1981; Miertuš et al., 1982; Cossi et al.,1996). The ECD spectra of compound **6** were obtained by weighing the Boltzmann distribution rate of each geometric conformation (Tähtinen et al., 2003).

The ECD spectra were simulated by overlapping Gaussian functions for each transition according to:

$$\Delta\varepsilon(E) = \frac{1}{2.297 \times 10^{-39}} \times \frac{1}{\sqrt{2\pi\sigma}} \sum_{i}^{A} \Delta E_i R_i e^{-[(E-E_i)/(2\sigma)]^2} \quad (1)$$

The σ represented the width of the band at $1/e$ height, and ΔE_i and R_i were the excitation energies and rotational strengths for transition i, respectively. R_{vel} had been used in this work.

REFERENCES

Cossi, M., Barone, V., Cammi, R., Tomasi, J. (1996). Ab initio study of solvated molecules: a new implementation of the polarizable continuum model. *Chem. Phys. Lett. 255*, 327-335. doi:10.1016/0009-2614(96)00349-1

Dennington, R., Keith, T., Millam, J. GaussView, Version 5, *Semichem Inc.*, Shawnee Mission, KS, 2009.

Frisch, M. J., Trucks, G. W., Schlegel, H. B., Scuseria, G. E., Robb, M. A., Cheeseman, J. R., et al. Gaussian 09, Revision C.01, *Gaussian, Inc.*, Wallingford CT, 2010.

Miertuš, S., Scrocc, E., Tomasi, J. (1981) Electrostatic interaction of a solute with a continuum. A direct utilizaion of AB initio molecular potentials for the prevision of solvent effects. *Chem. Phys.*, *55*, 117-129. doi: 10.1016/0301-0104(81)85090-2

Miertuš, S., Tomasi, J. (1982). Approximate evaluations of the electrostatic free energy and internal energy changes in solution processes. *Chem. Phys.*, *65*, 239-245. doi:10.1016/0301-0104(82)85072-6

Smith, S. G., Goodman, J. M. (2010). Assigning stereochemistry to single diastereoisomers by GIAO NMR calculation: The DP4 probability. *J. Am. Chem. Soc.*, *132*, 12946-12959. doi:10.1021/ja105035r

Tähtinen, P., Bagno, A., Klika, K. D., Pihlaja, K. (2003). Modeling NMR parameters by DFT methods as an aid to the conformational analysis of cis-fused 7a (8a)-methyl octa (hexa) hydrocyclopenta [d] [1, 3] oxazines and [3, 1] benzoxazines. *J. Am. Chem. Soc. 125*, 4609-4618. doi: 10.1021/ja021237t

Table S1. Important thermodynamic parameters (a.u.) and Boltzmann distributions of the optimized compound **6** at B3LYP/6-31G (d, p) level in the gas phase

Conformations	E+ZPE	G	%
**6-a**	-837.566993	-837.611517	0.1
**6-b**	-837.566388	-837.610699	0.1
**6-c**	-837.572374	-837.615961	15
**6-d**	-837.572216	-837.616240	20.2
**6-e**	-837.568086	-837.612755	0.5
**6-f**	-837.567487	-837.611995	0.2
**6-g**	-837.566283	-837.610572	0.1
**6-h**	-837.572023	-837.615888	13.9
**6-i**	-837.571908	-837.615952	14.9
**6-j**	-837.571653	-837.615561	9.8
**6-k**	-837.567745	-837.612577	0.4
**6-l**	-837.567250	-837.611606	0.1
**6-m**	-837.567892	-837.612074	0.2
**6-n**	-837.566617	-837.610876	0.1
**6-o**	-837.566054	-837.610206	0
**6-p**	-837.572011	-837.615663	10.9
**6-q**	-837.571886	-837.615825	13
**6-r**	-837.567729	-837.612196	0.3
**6-s**	-837.567879	-837.611823	0.2

E+ZPE, G: total energy with zero point energy (ZPE) and Gibbs free energy in the gas phase at B3LYP/6-31G(d,p) level., %: Boltzmann distributions, using the relative Gibbs free energies as weighting factors

Table S2. Optimized Z-matrixes of compound **6** in the gas phase (Å) at B3LYP/6-31 G (d, p) level

**6-a**				**6-b**			
C	-1.61212	-1.39916	1.175353	C	-2.08876	-1.52322	0.795535
C	-2.60968	-2.33637	0.87043	C	-3.32413	-2.0476	0.3887
C	-3.48182	-2.10905	-0.19512	C	-4.14892	-1.31535	-0.46636
C	-3.38535	-0.95454	-0.98326	C	-3.77122	-0.05084	-0.93623
C	-2.39317	-0.03649	-0.66242	C	-2.54507	0.452367	-0.51914
C	-1.51477	-0.24777	0.410656	C	-1.70314	-0.27233	0.338245
C	-1.00545	1.818721	-0.70836	C	-0.72794	1.859028	-0.21122
C	-0.58217	0.946189	0.53361	C	-0.46205	0.556301	0.633726
O	-0.50254	2.856299	-1.07107	O	-0.01411	2.83056	-0.29948
N	-2.07903	1.174408	-1.29206	N	-1.94559	1.675684	-0.83991

C	0.874425	0.610275	0.525975	C	0.791796	-0.12556	0.187728
O	1.338983	-0.31728	-0.37766	O	1.989252	0.501989	0.447347
C	2.685851	-0.38278	-0.14628	C	2.938325	-0.31682	-0.08329
N	3.089107	0.40264	0.804655	N	2.455163	-1.3861	-0.64484
C	1.939194	1.052538	1.241154	C	1.081746	-1.27652	-0.47903
O	-0.87113	1.772921	1.66103	O	-0.37565	1.009121	1.98251
C	3.480064	-1.31935	-0.99652	C	4.362824	0.111864	0.050679
C	4.962688	-1.3466	-0.61975	C	5.343622	-0.88386	-0.57134
H	-0.91926	-1.58025	1.99332	H	-1.44261	-2.09366	1.457325
H	-2.69965	-3.24277	1.46019	H	-3.63863	-3.02521	0.739165
H	-4.24951	-2.8416	-0.42584	H	-5.10332	-1.72984	-0.77694
H	-4.06204	-0.78849	-1.81564	H	-4.41746	0.513557	-1.60119
H	-2.54346	1.549599	-2.10581	H	-2.35024	2.390548	-1.42628
H	1.958651	1.795043	2.023612	H	0.3994	-2.02429	-0.85366
H	-0.83261	1.206085	2.444531	H	-0.11858	0.25115	2.526129
H	3.041028	-2.32225	-0.91551	H	4.469406	1.101805	-0.41116
H	3.355431	-1.02554	-2.04709	H	4.582578	0.25888	1.116109
H	5.504982	-2.03837	-1.27032	H	6.370232	-0.52684	-0.45149
H	5.409181	-0.35415	-0.71752	H	5.259598	-1.86546	-0.09825
H	5.098021	-1.66589	0.416675	H	5.14412	-1.01595	-1.63762
**6-c**				**6-d**			
C	-1.3819	-1.73956	0.749629	C	-1.83537	-1.25396	1.291677
C	-2.35052	-2.6467	0.298317	C	-2.81029	-2.19227	0.925359
C	-3.36995	-2.22513	-0.5571	C	-3.49801	-2.05837	-0.28214
C	-3.45773	-0.89335	-0.98222	C	-3.24037	-0.99144	-1.15304
C	-2.49304	-0.00845	-0.51925	C	-2.27782	-0.06781	-0.76778
C	-1.45859	-0.41944	0.33453	C	-1.57905	-0.19117	0.441463
C	-1.30624	1.927653	-0.11444	C	-0.90128	1.78186	-0.73507
C	-0.57997	0.775164	0.647759	C	-0.5935	0.955438	0.560181
O	-1.00008	3.10488	-0.07437	O	-0.42488	2.868733	-0.99592
N	-2.3676	1.367239	-0.78237	N	-1.844	1.084395	-1.44969
C	0.821321	0.654297	0.131716	C	0.840755	0.520719	0.609386
O	1.533935	-0.47826	0.461542	O	1.370682	-0.12979	-0.47966
C	2.76739	-0.29034	-0.09131	C	2.668019	-0.3933	-0.1292
N	2.889297	0.836612	-0.72533	N	2.980228	0.02645	1.057556
C	1.652189	1.454307	-0.58815	C	1.81743	0.616575	1.545335
O	-0.61243	1.051655	2.047662	O	-0.87878	1.771806	1.686548
C	3.777505	-1.37299	0.105094	C	3.51515	-1.10462	-1.13263
C	5.114781	-1.06445	-0.57121	C	4.948971	-1.32374	-0.6457
H	-0.58492	-2.05859	1.412194	H	-1.29089	-1.35167	2.225787
H	-2.30774	-3.68333	0.61666	H	-3.02974	-3.02772	1.582234
H	-4.11272	-2.93869	-0.90099	H	-4.24811	-2.79377	-0.55732
H	-4.25398	-0.56817	-1.64446	H	-3.77763	-0.8929	-2.09113

H	-3.0332	1.926868	-1.29568	H	-2.25956	1.453067	-2.29288
H	1.44321	2.427145	-1.00568	H	1.76543	1.06125	2.526925
H	-0.21816	1.93243	2.154028	H	-0.45785	2.6281	1.502072
H	3.916604	-1.527	1.183213	H	3.038854	-2.06389	-1.37448
H	3.358609	-2.31313	-0.27677	H	3.50823	-0.52653	-2.06582
H	5.822281	-1.88011	-0.39822	H	5.531027	-1.84783	-1.40884
H	4.988338	-0.93652	-1.64911	H	5.438053	-0.37096	-0.42856
H	5.546853	-0.14009	-0.18022	H	4.964569	-1.91661	0.272125
**6-e**				**6-f**			
C	-1.79924	-1.25598	1.286472	C	-2.16496	-1.41386	0.947208
C	-2.77656	-2.19742	0.935772	C	-3.36718	-1.96761	0.48486
C	-3.48544	-2.06098	-0.25971	C	-4.08888	-1.33415	-0.52888
C	-3.24856	-0.98967	-1.13099	C	-3.64387	-0.13536	-1.10174
C	-2.27926	-0.06392	-0.76377	C	-2.45326	0.400699	-0.62652
C	-1.55636	-0.19259	0.431088	C	-1.71181	-0.23179	0.382159
C	-0.89094	1.793274	-0.75505	C	-0.69594	1.857651	-0.22998
C	-0.58616	0.971899	0.56113	C	-0.4677	0.588306	0.680377
O	-0.39053	2.842884	-1.09034	O	-0.04248	2.877152	-0.24236
N	-1.8673	1.093612	-1.44029	N	-1.8111	1.589595	-1.00421
C	0.85041	0.563379	0.609961	C	0.772102	-0.12245	0.241633
O	1.343157	-0.16945	-0.44234	O	1.975155	0.516548	0.425282
C	2.65675	-0.38956	-0.12411	C	2.906034	-0.33634	-0.08266
N	3.009289	0.129822	1.010572	N	2.403737	-1.4335	-0.56836
C	1.859718	0.749077	1.494566	C	1.03607	-1.30898	-0.3681
O	-0.78951	1.769537	1.718382	O	-0.29285	0.948987	2.042552
C	3.473564	-1.17065	-1.1004	C	4.335705	0.089775	-0.00938
C	4.918848	-1.36518	-0.63744	C	5.293734	-0.94367	-0.60485
H	-1.23644	-1.35238	2.210351	H	-1.59646	-1.89245	1.738752
H	-2.98104	-3.03642	1.592962	H	-3.73893	-2.89115	0.916744
H	-4.23804	-2.79785	-0.52415	H	-5.01754	-1.77323	-0.88108
H	-3.80509	-0.88942	-2.05766	H	-4.21386	0.355482	-1.88447
H	-2.24696	1.425942	-2.31467	H	-2.1877	2.276147	-1.64176
H	1.840734	1.284151	2.430528	H	0.341178	-2.0752	-0.67615
H	-1.73729	1.944246	1.794093	H	-0.98096	1.587337	2.277167
H	2.987113	-2.14072	-1.26691	H	4.436321	1.054112	-0.52387
H	3.445802	-0.65458	-2.06906	H	4.584069	0.288468	1.041196
H	5.479938	-1.93829	-1.38086	H	6.32568	-0.58987	-0.52851
H	5.415345	-0.40268	-0.49233	H	5.214159	-1.89939	-0.08086
H	4.956345	-1.89981	0.314955	H	5.066657	-1.12773	-1.6579
**6-g**				**6-h**			
C	-2.27001	-1.44281	0.651416	C	1.184883	1.812144	0.573917
C	-3.56878	-1.73553	0.21048	C	2.178753	2.706121	0.152373
C	-4.27495	-0.80663	-0.55498	C	3.30963	2.240059	-0.52045

C	-3.71359	0.429655	-0.90011	C	3.486148	0.876308	-0.78724
C	-2.427	0.701623	-0.45138	C	2.4942	0.005752	-0.35575
C	-1.70299	-0.22261	0.317167	C	1.348873	0.460606	0.314551
C	-0.42063	1.78469	-0.03001	C	1.318845	-1.92436	0.105951
C	-0.35439	0.379987	0.679562	C	0.468802	-0.7294	0.640647
O	0.432067	2.641746	-0.03384	O	1.043541	-3.10232	0.239021
N	-1.65188	1.849267	-0.65542	N	2.440103	-1.39369	-0.48389
C	0.788092	-0.43069	0.157553	C	-0.85529	-0.71825	-0.06017
O	2.061532	-0.00807	0.463995	O	-1.64052	0.406619	0.065483
C	2.887367	-0.89857	-0.15131	C	-2.78373	0.116434	-0.6227
N	2.258172	-1.82941	-0.80865	N	-2.78412	-1.06849	-1.15655
C	0.914246	-1.54238	-0.61874	C	-1.55755	-1.61556	-0.80263
O	-0.20943	0.68	2.065762	O	0.323781	-0.85917	2.054388
C	4.356992	-0.69023	-0.00355	C	-3.85322	1.155748	-0.65755
C	4.837681	0.648691	-0.58953	C	-4.44433	1.466391	0.72848
H	-1.71669	-2.16629	1.244131	H	0.301737	2.165203	1.094847
H	-4.02474	-2.68681	0.464732	H	2.06836	3.767345	0.350786
H	-5.27968	-1.04206	-0.89297	H	4.070897	2.943641	-0.84383
H	-4.26813	1.147898	-1.4959	H	4.368528	0.517039	-1.30746
H	-1.94331	2.670213	-1.16504	H	3.182056	-1.97602	-0.84461
H	0.13389	-2.14658	-1.05618	H	-1.26381	-2.61184	-1.09548
H	-0.07713	-0.15838	2.530316	H	-0.05275	-1.74176	2.202525
H	4.617385	-0.73827	1.061404	H	-3.44425	2.074175	-1.0969
H	4.847141	-1.53275	-0.49771	H	-4.62723	0.786425	-1.33475
H	5.914167	0.765948	-0.4349	H	-5.216	2.237537	0.648757
H	4.639984	0.698059	-1.66433	H	-4.89842	0.573696	1.168389
H	4.327492	1.489296	-0.11199	H	-3.67154	1.826818	1.413106
**6-i**				**6-j**			
C	-1.69783	-0.96933	1.500555	C	-2.29885	-1.38501	0.769859
C	-2.63537	-1.98929	1.286888	C	-3.58557	-1.69852	0.309361
C	-3.296	-2.09324	0.061476	C	-4.25506	-0.82944	-0.55385
C	-3.04764	-1.18885	-0.97943	C	-3.6707	0.371339	-0.97787
C	-2.12246	-0.18026	-0.74489	C	-2.39861	0.66804	-0.50592
C	-1.45148	-0.06508	0.480918	C	-1.71175	-0.2001	0.356203
C	-0.80214	1.691281	-1.01228	C	-0.42403	1.775092	-0.06036
C	-0.50303	1.116635	0.414274	C	-0.36014	0.394884	0.680354
O	-0.35216	2.730812	-1.45169	O	0.402606	2.660925	0.020613
N	-1.7049	0.847237	-1.61122	N	-1.6072	1.799719	-0.76527
C	0.941417	0.744267	0.568292	C	0.769036	-0.43492	0.149282
O	1.518479	-0.0665	-0.37927	O	2.042921	-0.01509	0.458989
C	2.813372	-0.22524	0.040028	C	2.869782	-0.91704	-0.14817
N	3.080232	0.401634	1.144533	N	2.236215	-1.84285	-0.8039
C	1.889405	1.030379	1.495534	C	0.890916	-1.54763	-0.62154

O	-0.83996	2.108285	1.373222	O	-0.22157	0.64523	2.074432
C	3.710225	-1.07353	-0.797	C	4.339689	-0.71864	0.008241
C	3.24307	-2.53511	-0.90466	C	4.840443	0.603554	-0.59849
H	-1.1743	-0.88268	2.447629	H	-1.77012	-2.05132	1.444023
H	-2.84712	-2.70268	2.076746	H	-4.06332	-2.61981	0.62633
H	-4.0171	-2.89017	-0.09381	H	-5.2504	-1.08361	-0.9058
H	-3.56364	-1.27525	-1.93056	H	-4.19819	1.044256	-1.64658
H	-2.10992	1.048228	-2.51409	H	-1.91283	2.620265	-1.26839
H	1.798012	1.635958	2.383767	H	0.10918	-2.15206	-1.05576
H	-0.44044	2.932591	1.048595	H	0.536921	1.245012	2.161611
H	3.780037	-0.63584	-1.80086	H	4.591966	-0.75167	1.075692
H	4.70465	-1.01571	-0.34761	H	4.825154	-1.57433	-0.4675
H	3.928603	-3.10821	-1.53568	H	5.917513	0.710406	-0.44032
H	3.209999	-3.00912	0.080772	H	4.648754	0.636576	-1.67499
H	2.243719	-2.59586	-1.34439	H	4.339658	1.460034	-0.1391
**6-k**				**6-l**			
C	1.637099	1.02414	1.467605	C	-2.3434	-1.34497	0.783194
C	2.576431	2.040582	1.246476	C	-3.6104	-1.64984	0.265826
C	3.274746	2.100396	0.038592	C	-4.21578	-0.79653	-0.65899
C	3.064751	1.155102	-0.97392	C	-3.5874	0.380796	-1.08599
C	2.132931	0.152198	-0.73441	C	-2.33477	0.668504	-0.55794
C	1.421062	0.084089	0.472136	C	-1.70951	-0.18652	0.361449
C	0.812198	-1.73802	-0.97802	C	-0.37972	1.781375	-0.00253
C	0.493729	-1.12158	0.44254	C	-0.35919	0.392529	0.747998
O	0.346834	-2.74877	-1.4538	O	0.42424	2.681344	0.099814
N	1.753118	-0.91217	-1.56522	N	-1.51505	1.782625	-0.79351
C	-0.95565	-0.77561	0.556874	C	0.764535	-0.43869	0.218399
O	-1.48124	0.09172	-0.36865	O	2.045972	-0.00705	0.461237
C	-2.80039	0.213589	-0.01844	C	2.847357	-0.92171	-0.1511
N	-3.12546	-0.48653	1.024526	N	2.192874	-1.87106	-0.75474
C	-1.95195	-1.13177	1.403985	C	0.857157	-1.57375	-0.52565
O	0.736488	-2.06611	1.474732	O	-0.14822	0.556174	2.142713
C	-3.65305	1.113453	-0.84757	C	4.321912	-0.71716	-0.05773
C	-3.21143	2.586316	-0.80164	C	4.790541	0.603604	-0.69217
H	1.083071	0.968103	2.400052	H	-1.86309	-1.99601	1.507253
H	2.759918	2.785072	2.014405	H	-4.12285	-2.55145	0.58543
H	3.997755	2.894172	-0.1242	H	-5.19617	-1.04351	-1.05531
H	3.61291	1.207056	-1.90951	H	-4.06733	1.04329	-1.79961
H	2.134293	-1.10451	-2.48003	H	-1.77412	2.588863	-1.34352
H	-1.90691	-1.80419	2.245748	H	0.060054	-2.19076	-0.91154
H	1.689265	-2.22538	1.51206	H	-0.74491	1.248772	2.459572
H	-3.64187	0.759061	-1.88593	H	4.616593	-0.74074	0.998982
H	-4.67548	1.003825	-0.47767	H	4.791349	-1.57452	-0.54651

H	-3.86261	3.199339	-1.43165	H	5.872577	0.717081	-0.5784
H	-3.25873	2.976961	0.219195	H	4.555672	0.629232	-1.76044
H	-2.1848	2.699293	-1.16117	H	4.303454	1.459195	-0.21694
**6-m**				**6-n**			
C	-1.1713	-1.81259	0.5469	C	1.718169	1.107612	1.359368
C	-2.16168	-2.70718	0.119045	C	2.74508	2.030093	1.112368
C	-3.29689	-2.23775	-0.545	C	3.497302	1.943205	-0.05991
C	-3.48127	-0.87191	-0.79489	C	3.249432	0.946938	-1.01359
C	-2.49073	0.000233	-0.36016	C	2.230513	0.040471	-0.74855
C	-1.33968	-0.45789	0.299165	C	1.471796	0.110507	0.429282
C	-1.31414	1.939165	0.115791	C	0.720863	-1.70524	-0.95657
C	-0.46494	0.732974	0.662121	C	0.476197	-1.03761	0.449488
O	-1.04789	3.119556	0.198014	O	0.116322	-2.63786	-1.43173
N	-2.44496	1.398039	-0.46428	N	1.777105	-1.02705	-1.53369
C	0.861854	0.738899	-0.02303	C	-0.94907	-0.63373	0.649193
O	1.635254	-0.39693	0.069684	O	-1.43424	0.458332	-0.03154
C	2.776685	-0.09965	-0.61756	C	-2.74798	0.54651	0.343487
N	2.783727	1.097494	-1.12349	N	-3.11128	-0.3754	1.182629
C	1.565181	1.647198	-0.74814	C	-1.9701	-1.14389	1.383398
O	-0.22504	0.853959	2.063615	O	0.814114	-2.05882	1.388327
C	3.83914	-1.14482	-0.68022	C	-3.56298	1.646222	-0.24916
C	4.445249	-1.47714	0.694351	C	-3.71084	1.538548	-1.7766
H	-0.28279	-2.16596	1.059305	H	1.118089	1.182048	2.262852
H	-2.0452	-3.77029	0.303513	H	2.951053	2.816483	1.831128
H	-4.05616	-2.94097	-0.87392	H	4.28895	2.663281	-0.24423
H	-4.36799	-0.51134	-1.30692	H	3.833631	0.890321	-1.92673
H	-3.16451	1.979007	-0.86936	H	2.137274	-1.28131	-2.4416
H	1.278205	2.653679	-1.00984	H	-1.96417	-2.01038	2.026142
H	-1.06289	0.688008	2.519881	H	0.889421	-1.63318	2.254258
H	3.42011	-2.05497	-1.12729	H	-4.5404	1.608784	0.238194
H	4.607391	-0.7702	-1.3611	H	-3.10574	2.610028	0.008218
H	5.212414	-2.25065	0.595195	H	-4.31231	2.367967	-2.15991
H	4.907749	-0.59223	1.140892	H	-2.73452	1.569899	-2.26815
H	3.678716	-1.8428	1.383237	H	-4.20196	0.602131	-2.05651
**6-o**				**6-p**			
C	-2.08043	-1.50868	0.770689	C	1.090953	1.706592	0.838239
C	-3.38691	-1.9215	0.471806	C	1.969315	2.721326	0.433929
C	-4.24415	-1.0773	-0.23537	C	3.029695	2.43601	-0.42806
C	-3.82971	0.191444	-0.66065	C	3.249142	1.137709	-0.90594
C	-2.53272	0.583062	-0.35207	C	2.372415	0.144869	-0.48896
C	-1.6578	-0.25498	0.356051	C	1.297709	0.418647	0.370032
C	-0.59515	1.84215	-0.16105	C	1.377755	-1.91211	-0.17158
C	-0.33506	0.467229	0.561944	C	0.536437	-0.86656	0.625399

O	0.173966	2.768772	-0.26641	O	1.188637	-3.11431	-0.17907
N	-1.88398	1.784899	-0.65833	N	2.382945	-1.2248	-0.80767
C	0.810338	-0.26151	-0.06511	C	-0.86493	-0.86086	0.095073
O	2.070182	0.268445	0.095085	O	-1.68399	0.189212	0.447081
C	2.896671	-0.57236	-0.58636	C	-2.8904	-0.09858	-0.12418
N	2.279763	-1.5693	-1.15172	N	-2.89865	-1.21441	-0.79049
C	0.943993	-1.38155	-0.82816	C	-1.61052	-1.7157	-0.65537
O	-0.07029	0.815051	1.918914	O	0.582151	-1.19404	2.01378
C	4.354516	-0.25606	-0.59047	C	-4.00035	0.877978	0.074098
C	4.977128	-0.22675	0.81541	C	-3.72138	2.253561	-0.55582
H	-1.40893	-2.16629	1.316124	H	0.264108	1.918382	1.507206
H	-3.73136	-2.89998	0.790256	H	1.824612	3.734762	0.794173
H	-5.25368	-1.40604	-0.46333	H	3.701745	3.231656	-0.73542
H	-4.50156	0.843309	-1.21021	H	4.077367	0.919008	-1.57266
H	-2.29621	2.565067	-1.14823	H	3.104579	-1.69653	-1.33344
H	0.173675	-2.05666	-1.16894	H	-1.30601	-2.65269	-1.09566
H	0.179902	0.003032	2.381723	H	0.273045	-2.11189	2.08098
H	4.837944	-1.01008	-1.21657	H	-4.89597	0.427405	-0.3606
H	4.502831	0.716722	-1.07589	H	-4.18246	0.996632	1.149536
H	6.04067	0.020994	0.754167	H	-4.5592	2.932562	-0.37284
H	4.488327	0.523022	1.443282	H	-2.81917	2.703537	-0.13232
H	4.88266	-1.19967	1.307284	H	-3.58218	2.167319	-1.63744
**6-q**				**6-r**			
C	-2.01137	-1.02533	1.314048	C	-1.96648	0.939424	-1.38486
C	-3.03755	-1.89521	0.920127	C	-2.992	1.840669	-1.06804
C	-3.58518	-1.80409	-0.36074	C	-3.56043	1.837023	0.207556
C	-3.13314	-0.84837	-1.28023	C	-3.13185	0.94055	1.195318
C	-2.12243	0.009792	-0.86782	C	-2.11717	0.051741	0.861059
C	-1.56338	-0.07149	0.415386	C	-1.5346	0.048457	-0.41472
C	-0.57033	1.715022	-0.83308	C	-0.56023	-1.66316	0.968871
C	-0.48163	0.98307	0.549457	C	-0.47365	-1.03972	-0.48138
O	0.041034	2.723019	-1.12709	O	0.073241	-2.60125	1.396965
N	-1.50541	1.049853	-1.58771	N	-1.5265	-0.94604	1.650315
C	0.88763	0.421848	0.791335	C	0.903686	-0.53081	-0.75893
O	1.458717	-0.36723	-0.17789	O	1.433809	0.389238	0.111501
C	2.677813	-0.7234	0.336585	C	2.678423	0.664212	-0.39147
N	2.90483	-0.23322	1.516588	N	2.954583	0.007677	-1.47606
C	1.764955	0.503757	1.822875	C	1.824732	-0.76621	-1.72495
O	-0.795	1.920179	1.569633	O	-0.71734	-2.02496	-1.47504
C	3.556523	-1.58555	-0.50549	C	3.52434	1.63885	0.356144
C	3.979353	-0.91864	-1.82573	C	3.875074	1.174698	1.780193
H	-1.57482	-1.09119	2.305853	H	-1.51183	0.934363	-2.37121
H	-3.4065	-2.64433	1.613214	H	-3.34356	2.545311	-1.81471

H	-4.37685	-2.4862	-0.65603	H	-4.35218	2.541642	0.444037
H	-3.56188	-0.78295	-2.27536	H	-3.57985	0.942662	2.18416
H	-1.79438	1.379907	-2.49718	H	-1.78219	-1.16642	2.601734
H	1.65513	1.033305	2.756445	H	1.755567	-1.43613	-2.56707
H	-0.26954	2.712406	1.367855	H	-1.64812	-2.27947	-1.41635
H	4.430828	-1.83014	0.102801	H	4.430086	1.785678	-0.23747
H	3.034301	-2.52645	-0.72033	H	3.004177	2.604104	0.402294
H	4.613472	-1.59426	-2.40713	H	4.492305	1.924588	2.283651
H	3.106611	-0.66293	-2.43285	H	2.971402	1.020808	2.376389
H	4.542083	0.000216	-1.63706	H	4.430812	0.23283	1.758619
**6-s**							
C	1.08184	1.703488	0.824159				
C	1.958289	2.717708	0.415006				
C	3.018757	2.428017	-0.44614				
C	3.240524	1.127322	-0.91614				
C	2.363647	0.134191	-0.49689				
C	1.286959	0.412547	0.359089				
C	1.36566	-1.93144	-0.16865				
C	0.530689	-0.87603	0.645666				
O	1.177715	-3.12825	-0.22617				
N	2.378693	-1.23516	-0.79912				
C	-0.87019	-0.88076	0.127636				
O	-1.67798	0.186147	0.453397				
C	-2.88304	-0.0999	-0.12009				
N	-2.89837	-1.22725	-0.76677				
C	-1.61753	-1.74046	-0.61266				
O	0.477598	-1.20682	2.032636				
C	-3.98552	0.889272	0.056023				
C	-3.69027	2.253834	-0.58996				
H	0.252726	1.916604	1.490586				
H	1.811789	3.73328	0.768441				
H	3.6899	3.222647	-0.75815				
H	4.069955	0.906305	-1.58071				
H	3.069569	-1.70165	-1.36875				
H	-1.31935	-2.69011	-1.02873				
H	1.356546	-1.04643	2.406101				
H	-4.8817	0.440516	-0.37938				
H	-4.17507	1.02362	1.128339				
H	-4.52312	2.94285	-0.42205				
H	-2.78695	2.70162	-0.1663				
H	-3.54422	2.152221	-1.66939				

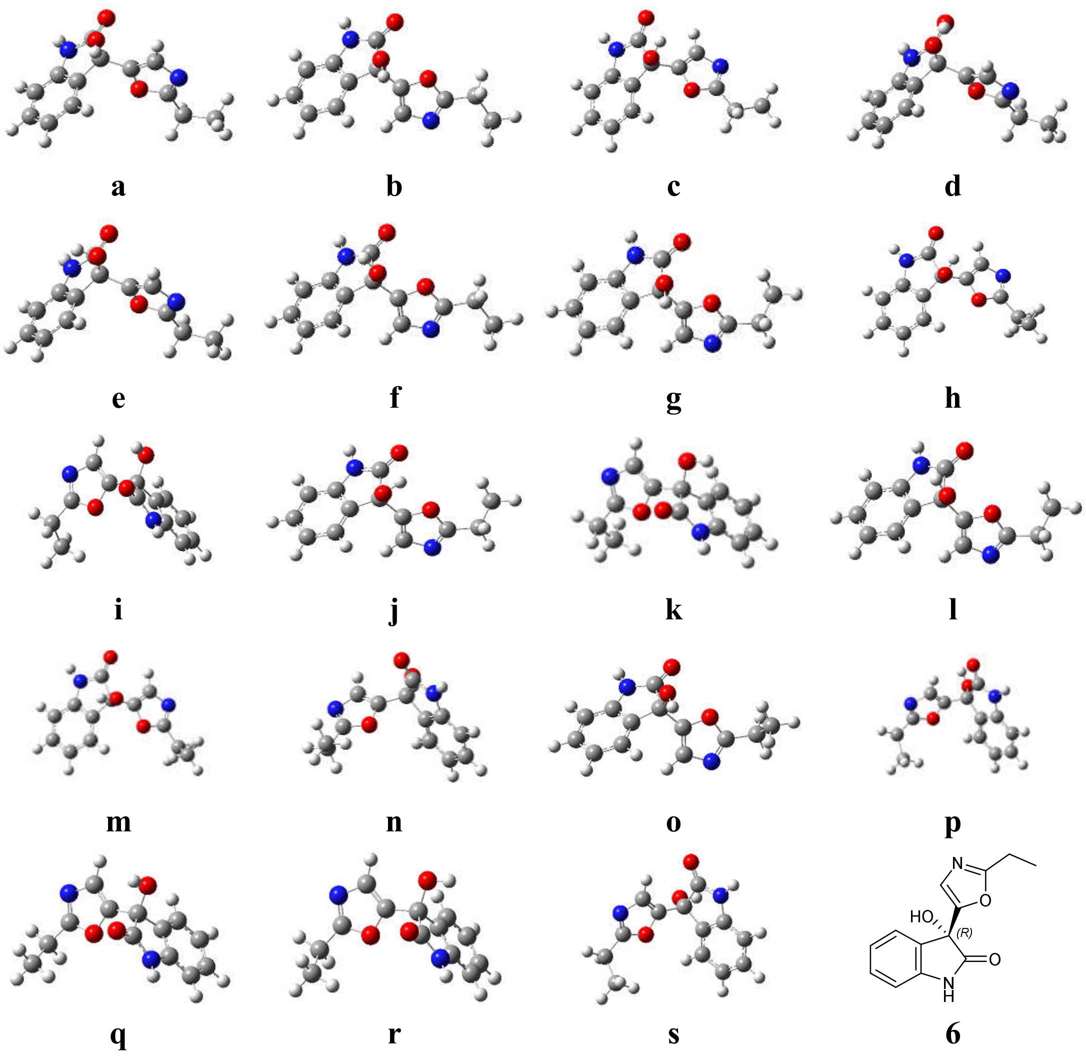

Figure S1. Optimized geometries of predominant conformers for compound **6** at the B3LYP/6-31G(d,p) level in the gas phase.

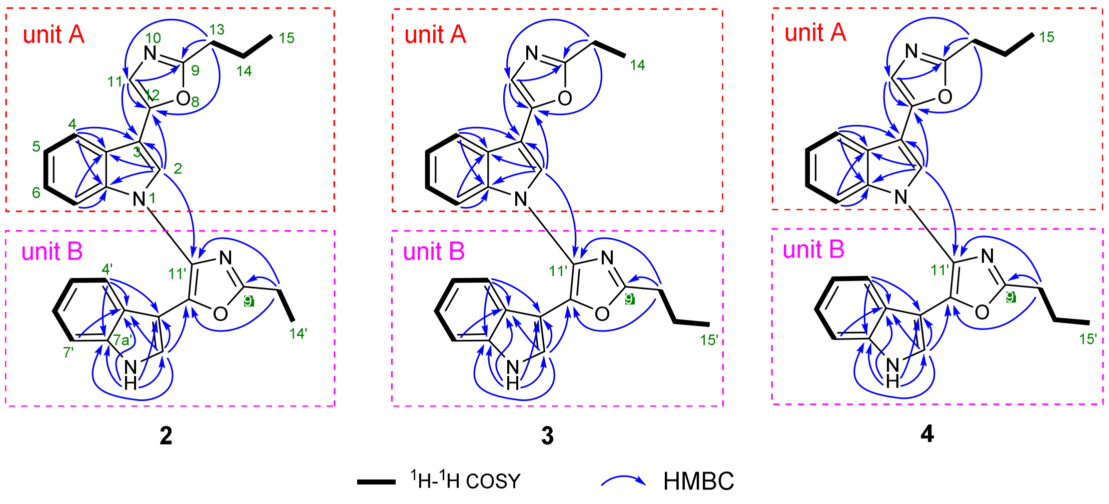

Figure S2. 2D NMR correlations of **2**, **3** and **4**.

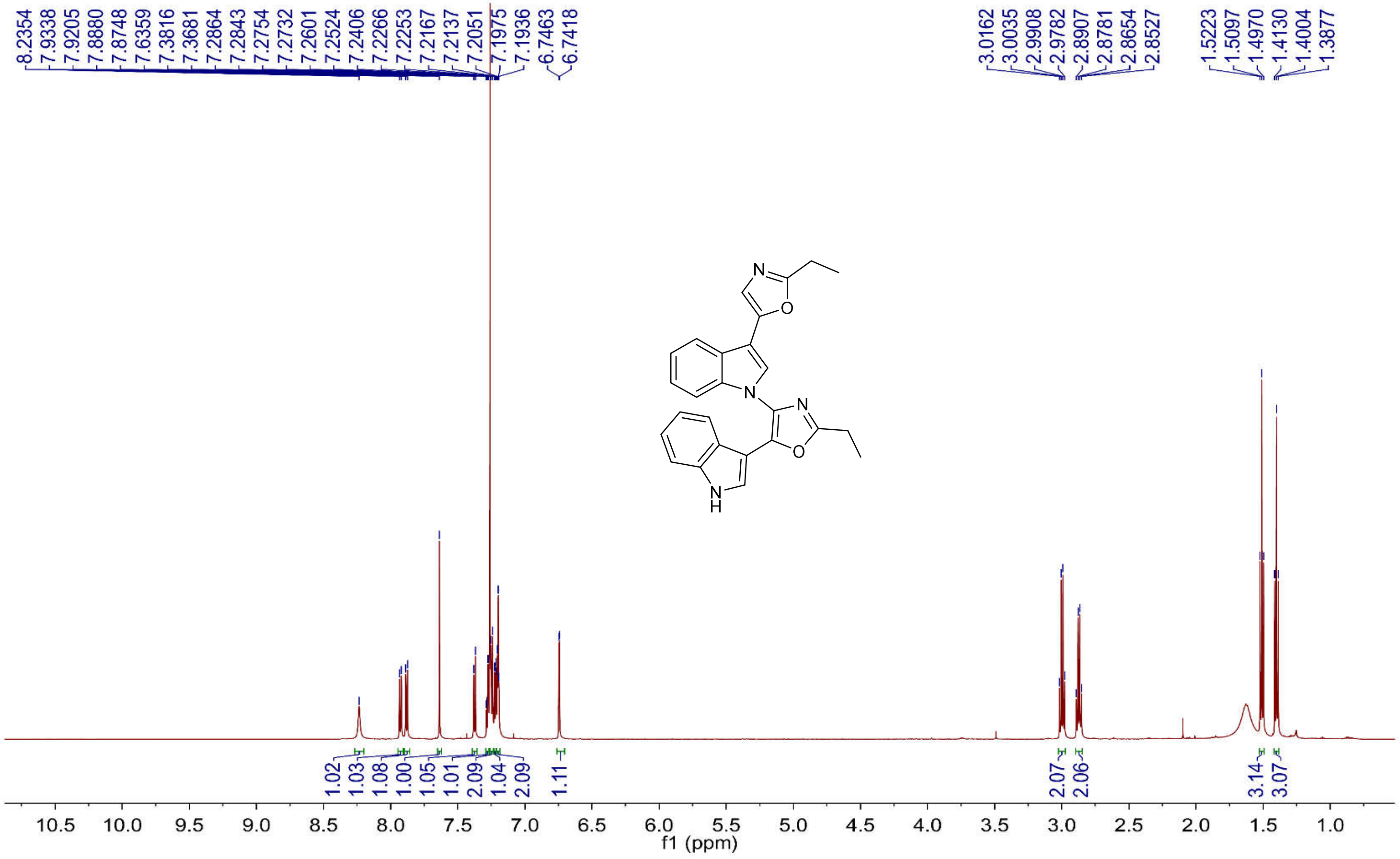

Figure S3. ^{1}H NMR (600 MHz) spectrum of compound **1** in $CDCl_3$.

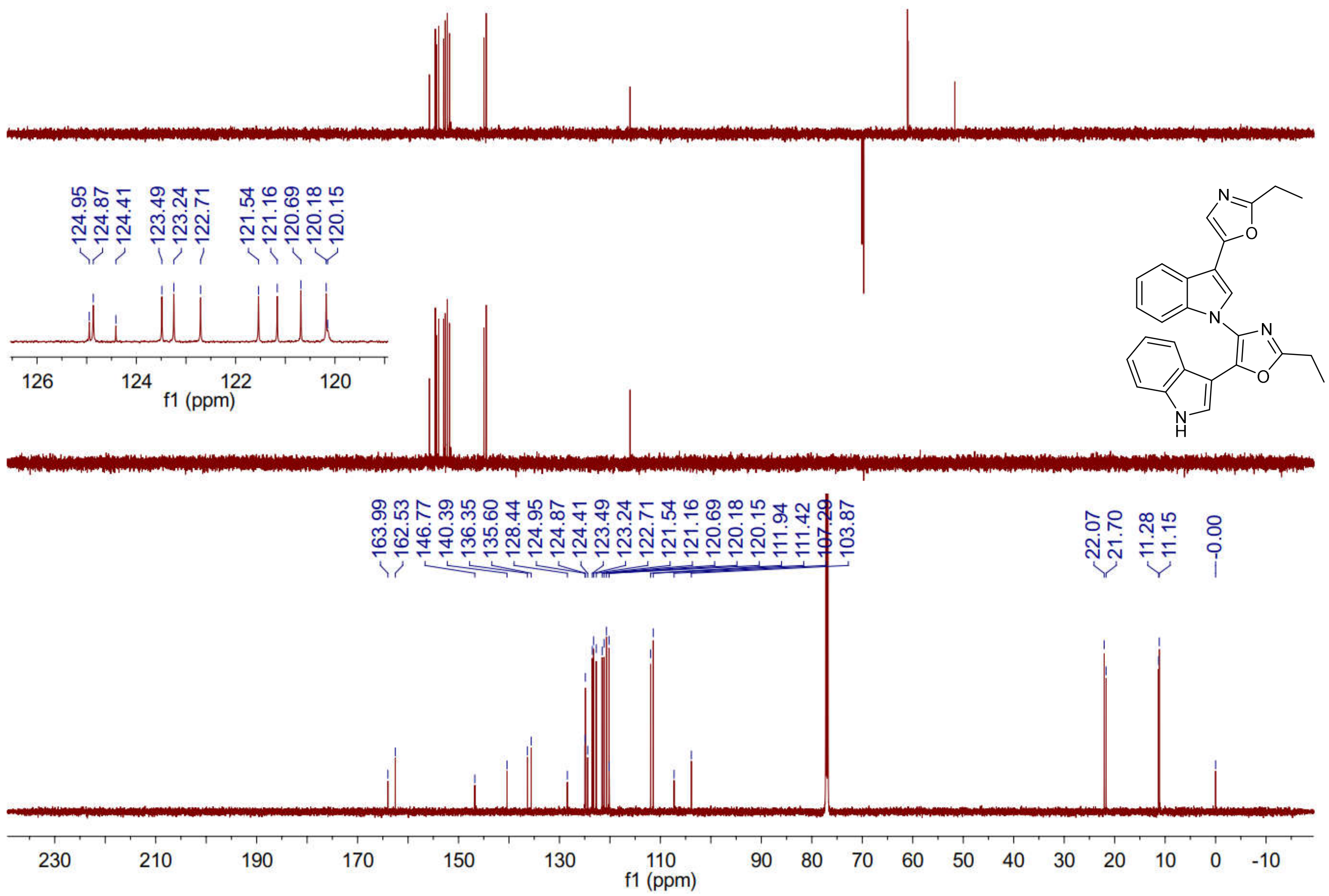

Figure S4. ^{13}C NMR and DEPT (150 MHz) spectrum of compound **1** in $CDCl_3$.

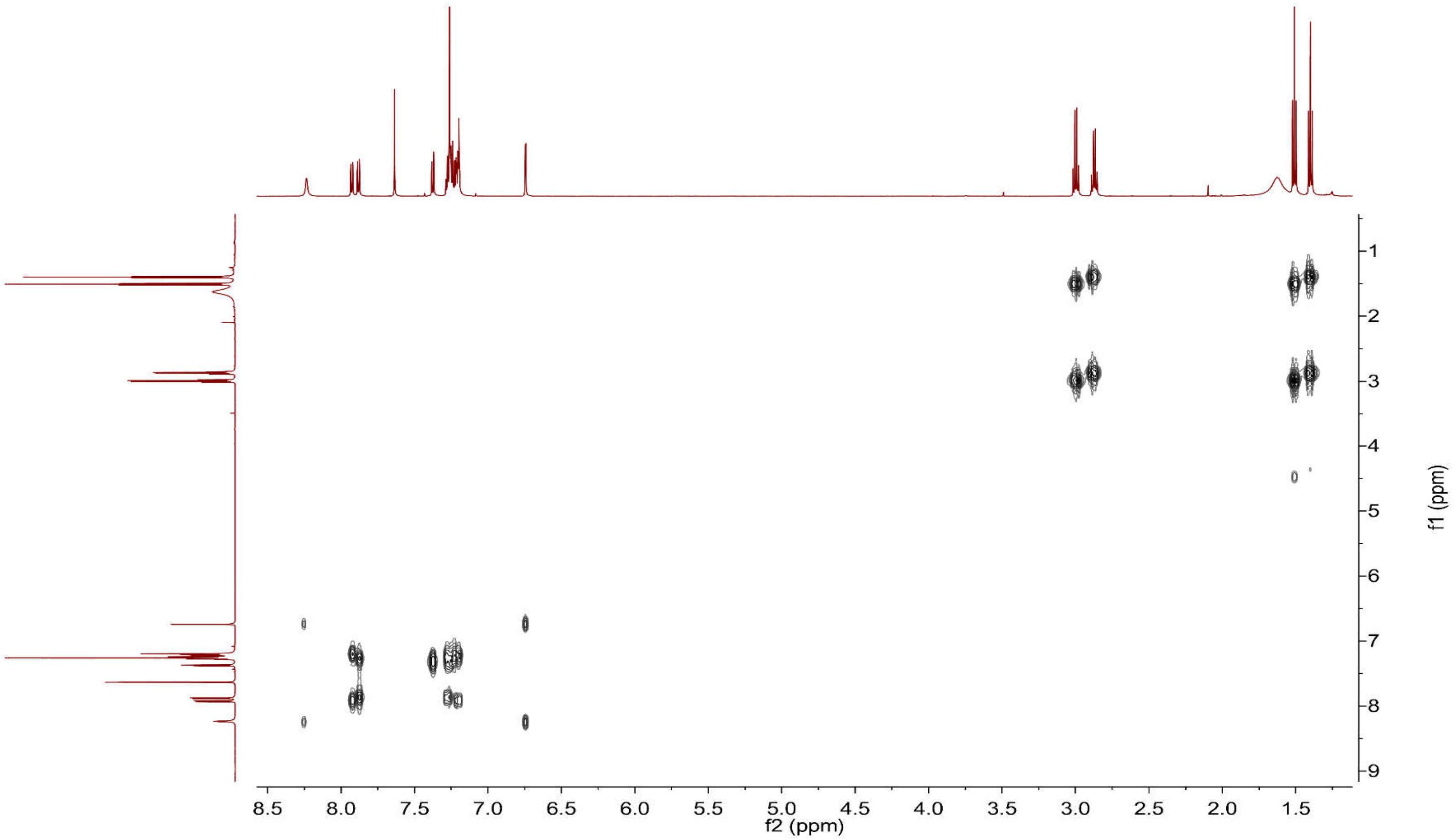

Figure S5. $^1H-^1H$ COSY (600 MHz) spectrum of compound **1** in $CDCl_3$.

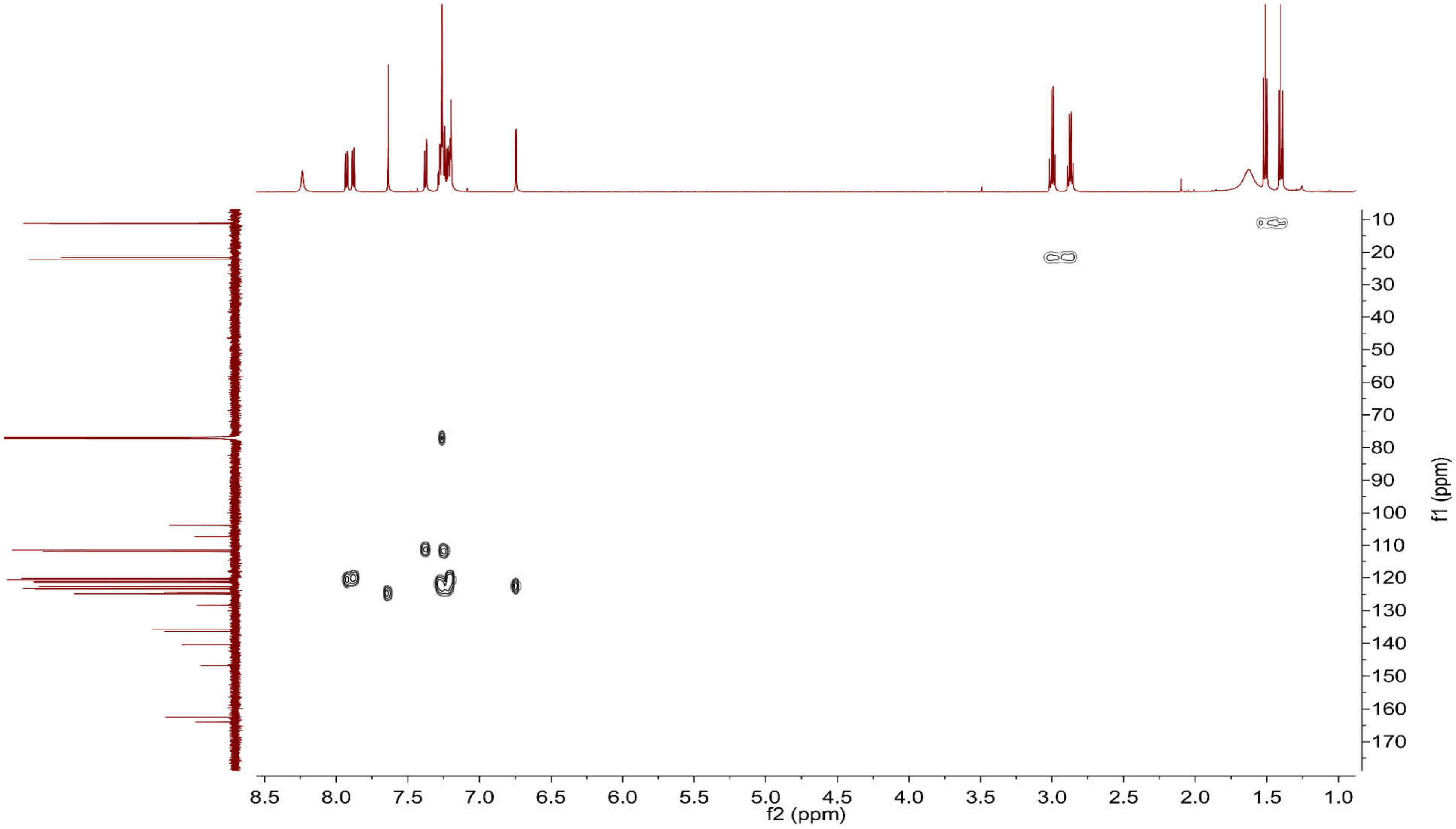

Figure S6. HSQC (600 MHz) spectrum of compound **1** in $CDCl_3$.

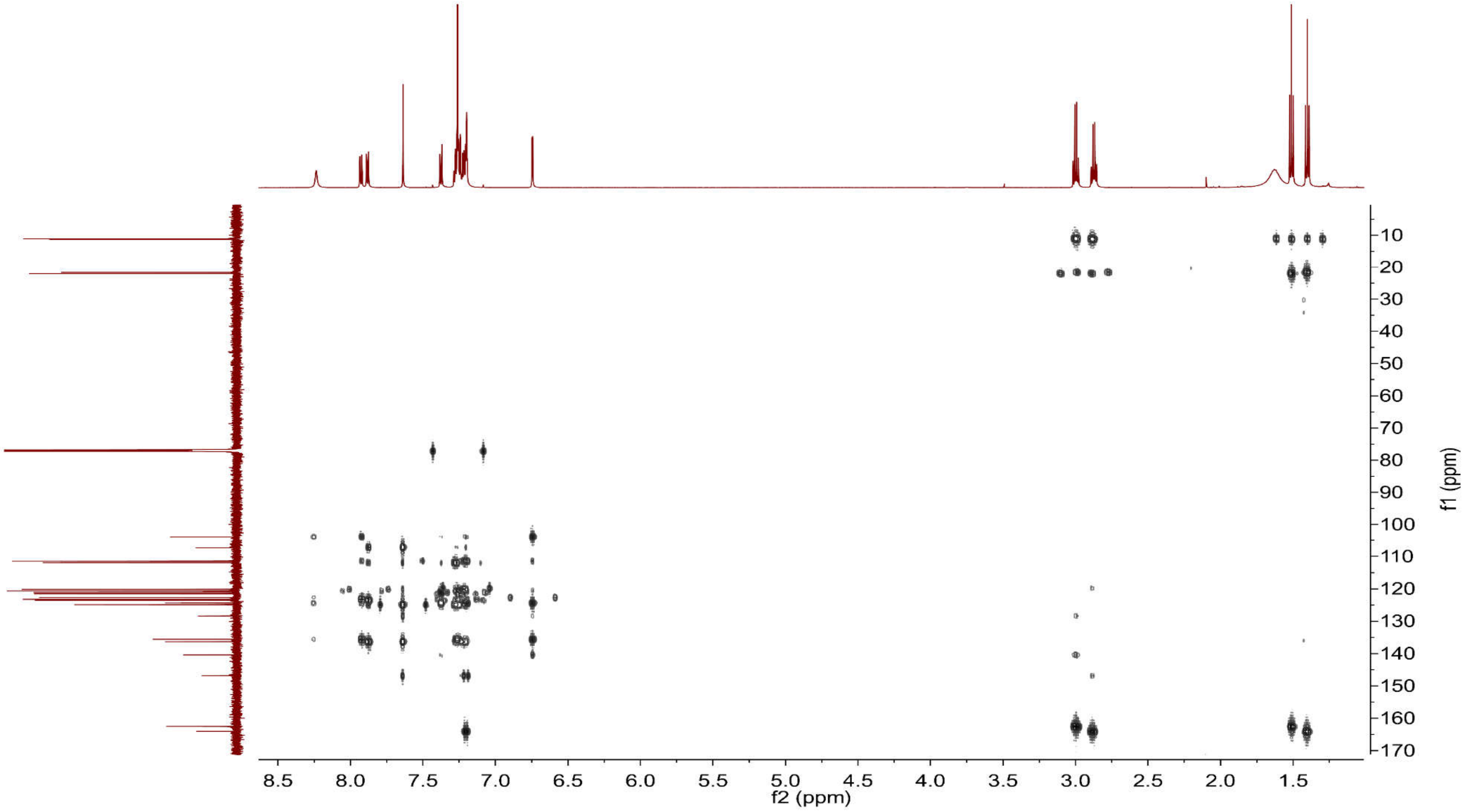

Figure S7. HMBC (600 MHz) spectrum of compound **1** in $CDCl_3$.

Data File: E:\DATA\2018\1213\HSXC905.lcd

Elmt	Val.	Min	Max	Elmt	Val.	Min	Max	Elmt	Val.	Min	Max	Elmt	Val.	Min	Max	Use Adduct
H	1	10	100	F	1	0	0	S	2	0	0	Br	1	0	0	H
C	4	10	50	Na	1	0	0	Cl	1	0	0	Ag	1	0	0	Cl
N	3	0	10	Mg	2	0	0	Cu	2	0	0	I	3	0	0	
O	2	0	20	Si	4	0	0	Se	2	0	0					

Error Margin (ppm):	5	DBE Range:	-2.0 - 100.0	Electron Ions:	both
HC Ratio:	unlimited	Apply N Rule:	yes	Use MSn Info:	yes
Max Isotopes:	all	Isotope RI (%):	1.00	Isotope Res:	10000
MSn Iso RI (%):	75.00	MSn Logic Mode:	OR	Max Results:	10

Event#: 2 MS(E-) Ret. Time : 0.467 Scan# : 72

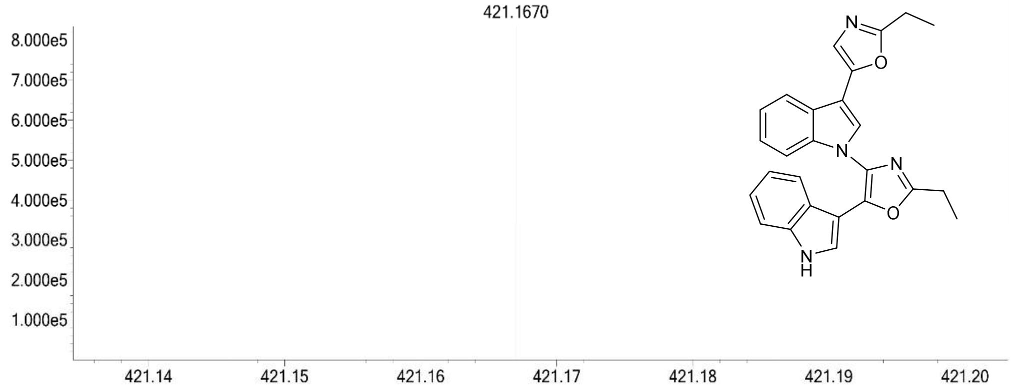

Measured region for 421.1670 m/z

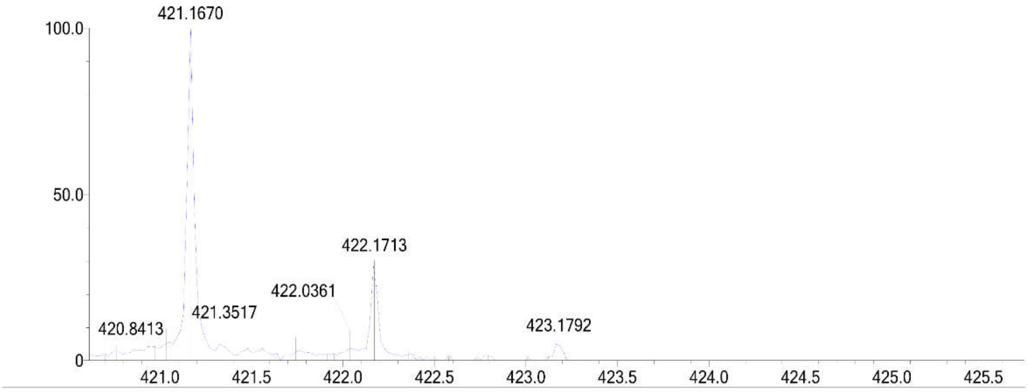

C26 H22 N4 O2 [M-H]- : Predicted region for 421.1670 m/z

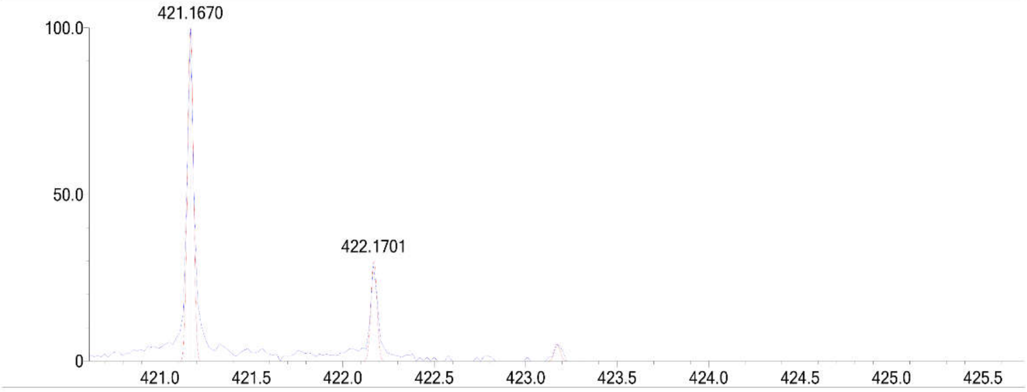

Formula (M)	Ion	Meas. m/z	Pred. m/z	Df. (mDa)	Df. (ppm)	DBE
C26 H22 N4 O2	[M-H]-	421.1670	421.1670	0.0	0.00	18.0

Figure S8. HRESIMS spectrum of compound **1**.

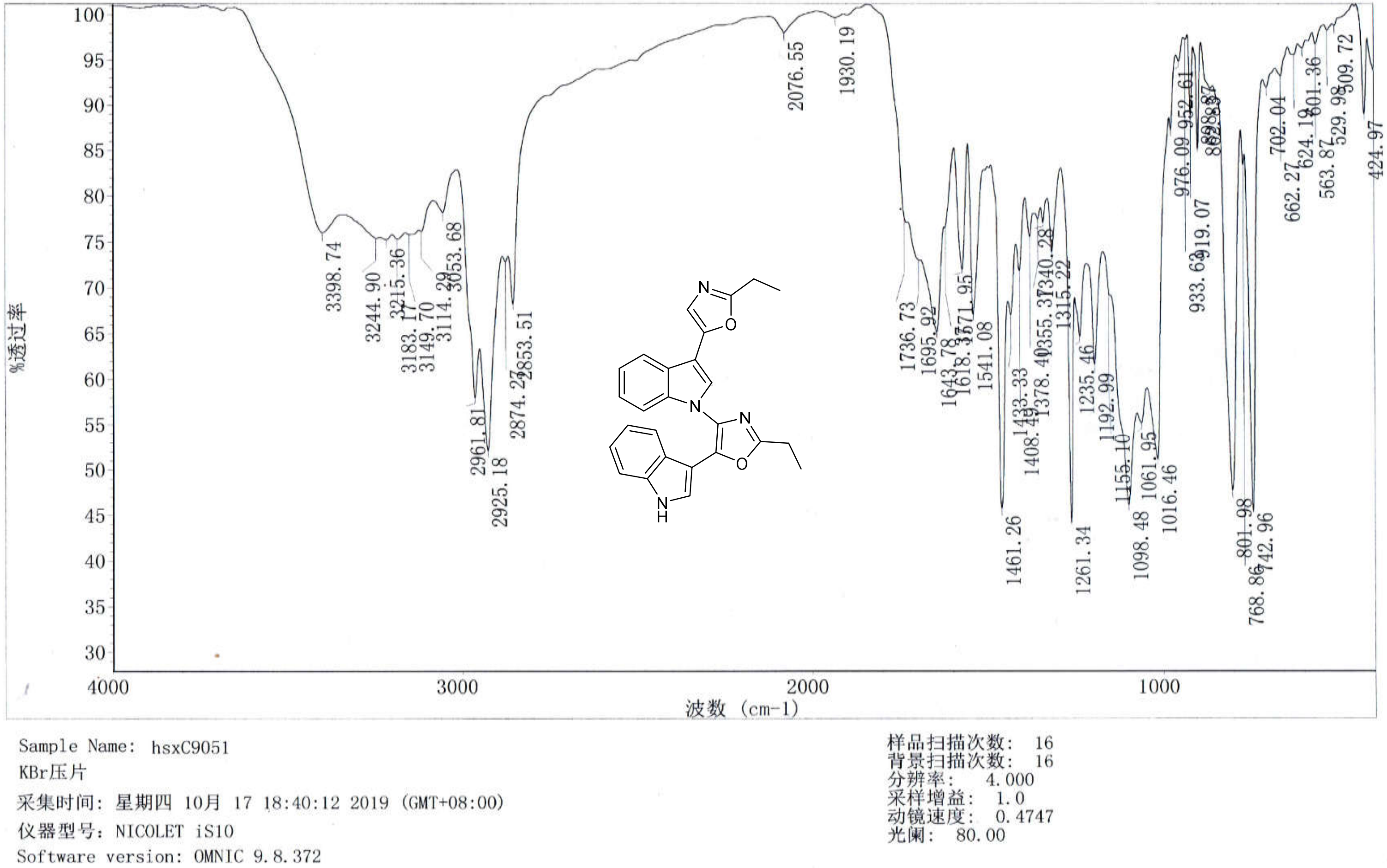

Figure S9. IR spectrum of compound **1**.

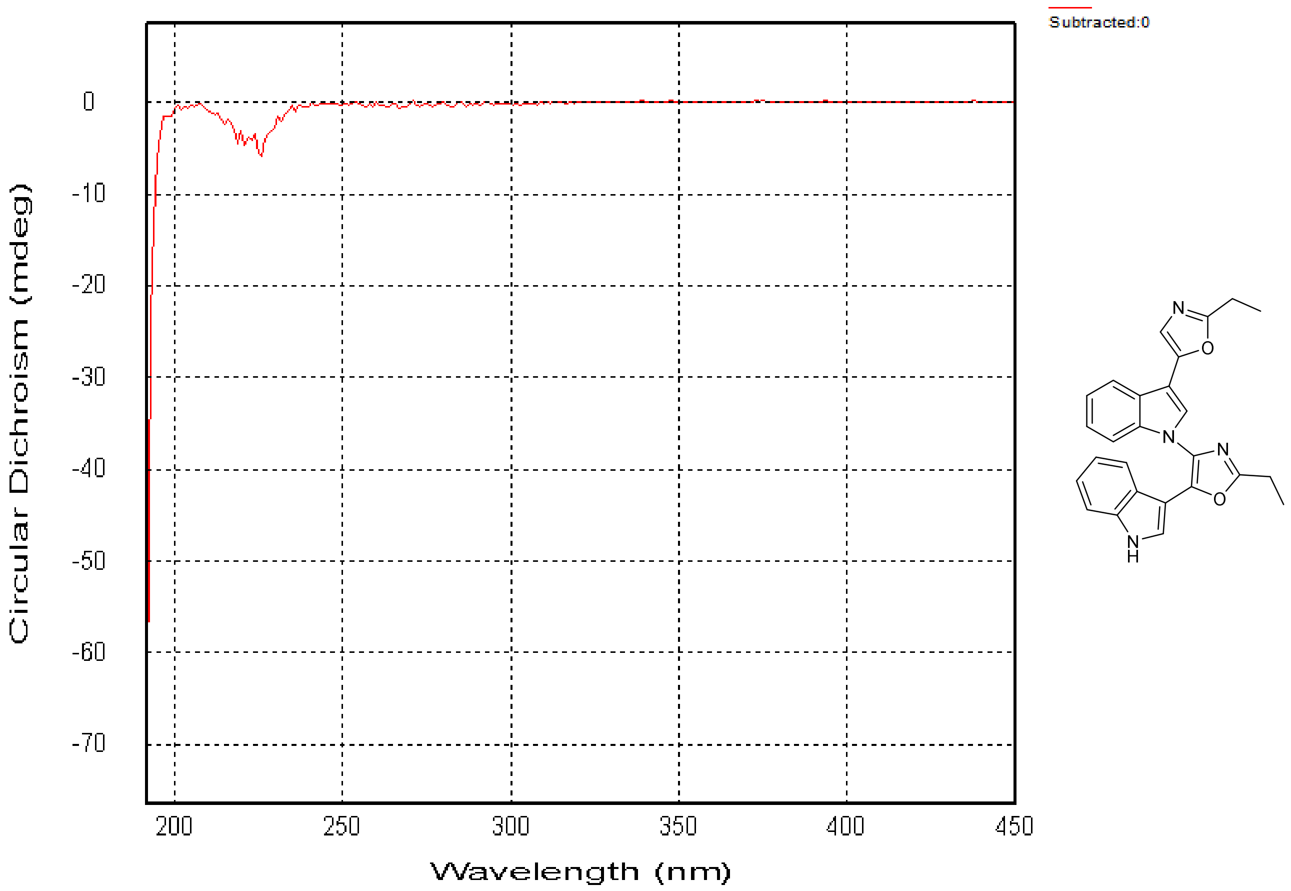

Figure S10. CD spectrum of compound **1**.

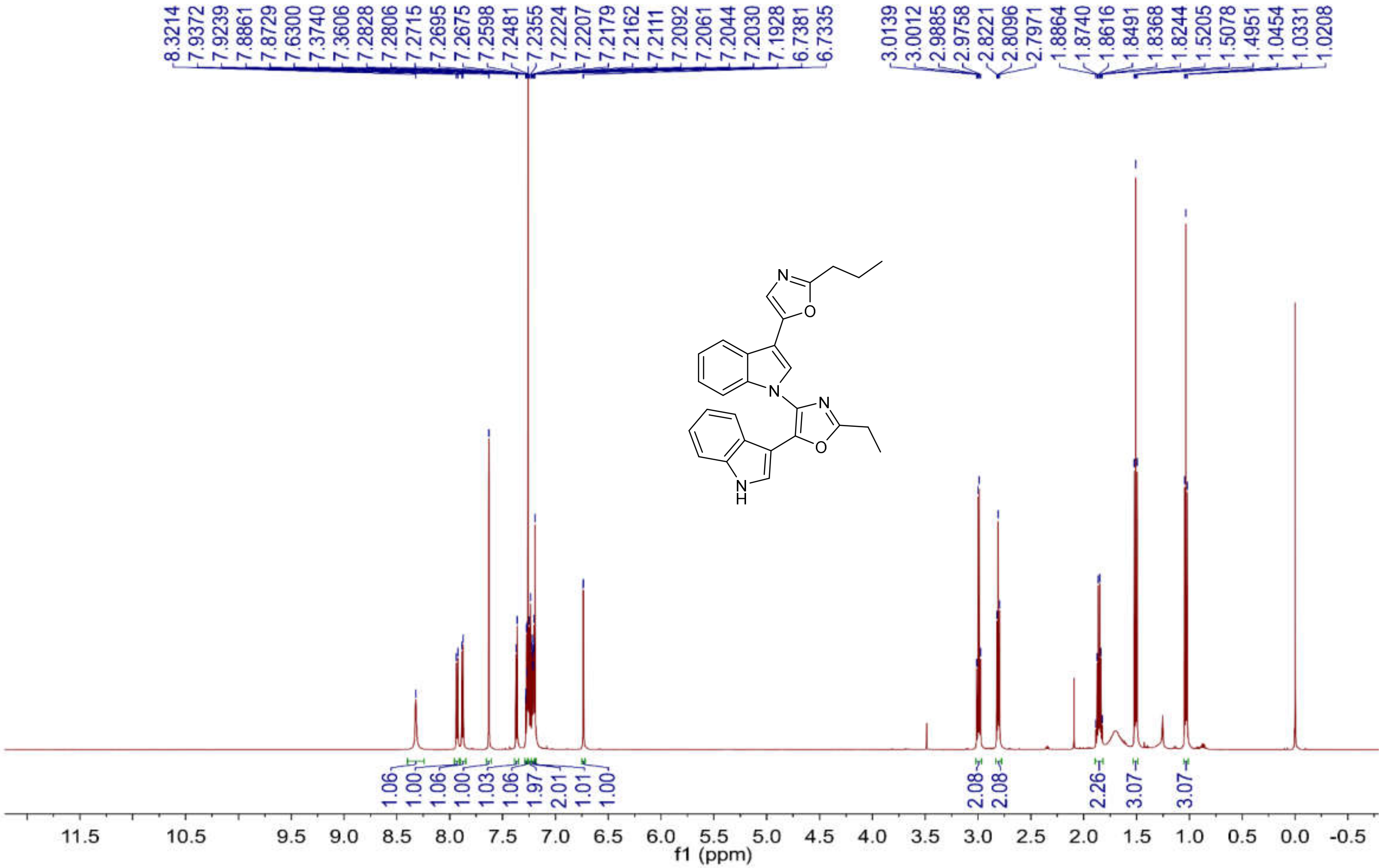

Figure S11. ^{1}H NMR (600 MHz) spectrum of compound **2** in $CDCl_3$.

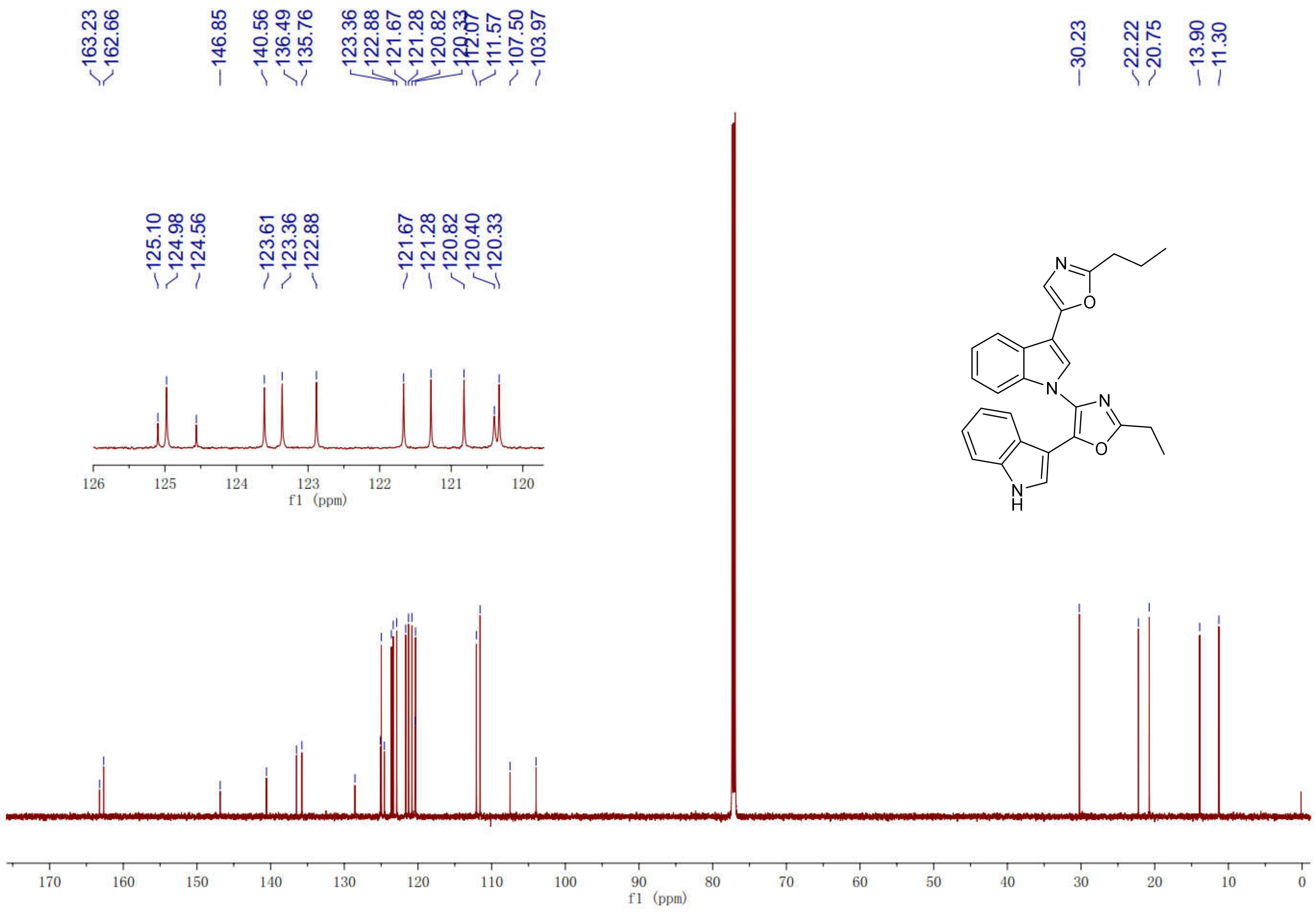

Figure S12. ^{13}C NMR (150 MHz) spectrum of compound **2** in CDCl3.

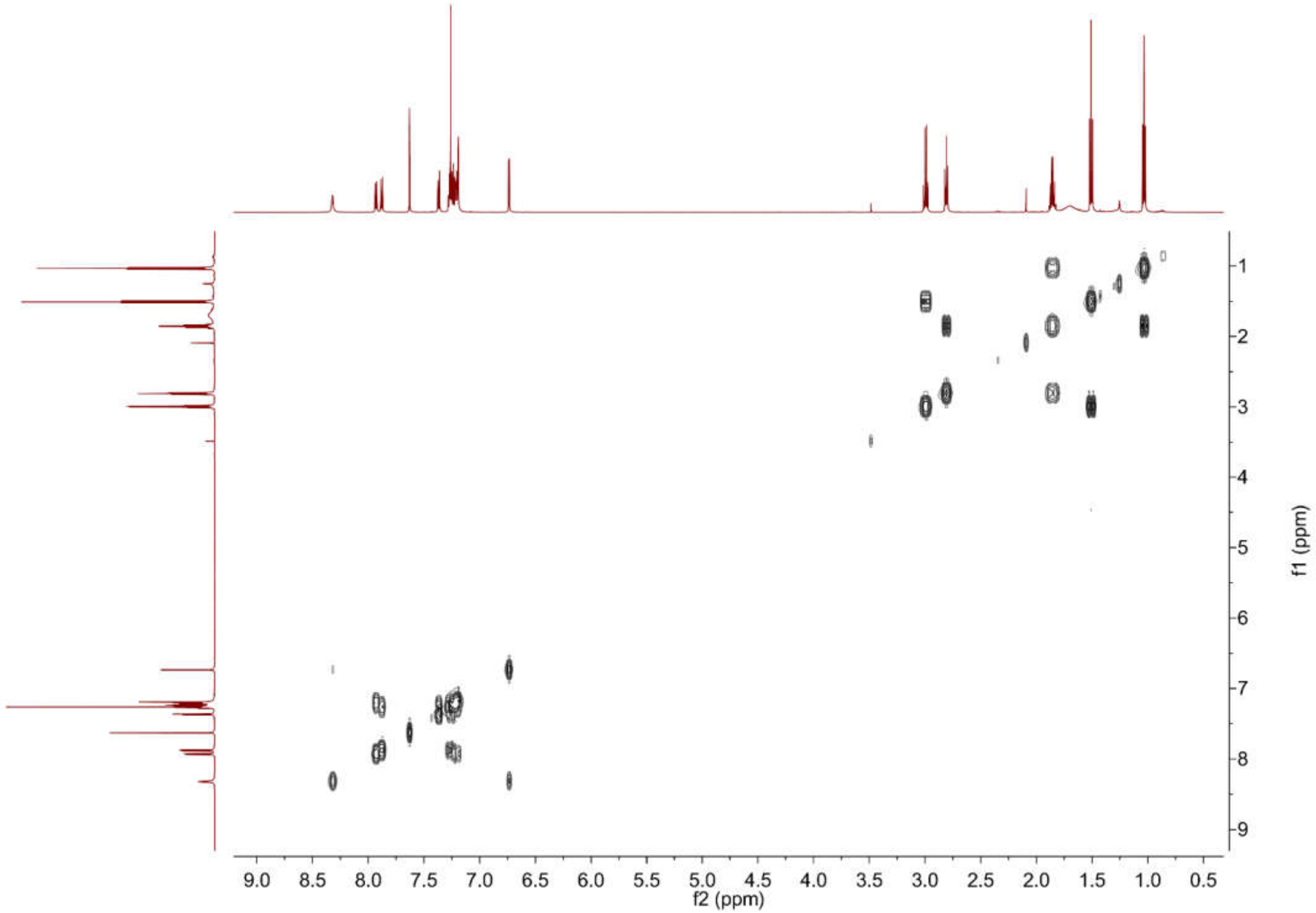

Figure S13. ^1H–^1H COSY (600 MHz) spectrum of compound **2** in $CDCl_3$.

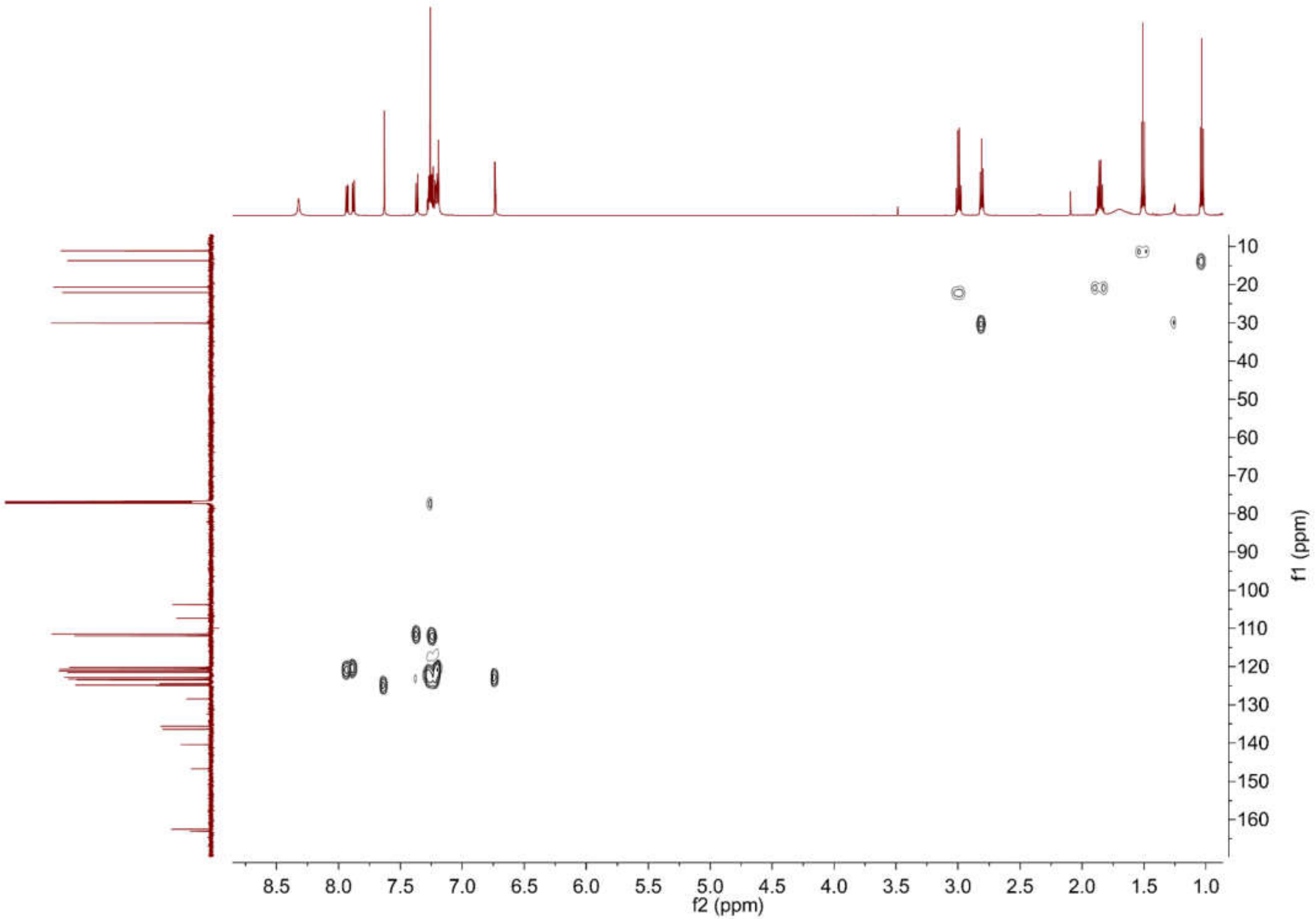

Figure S14. HSQC (600 MHz) spectrum of compound **2** in $CDCl_3$.

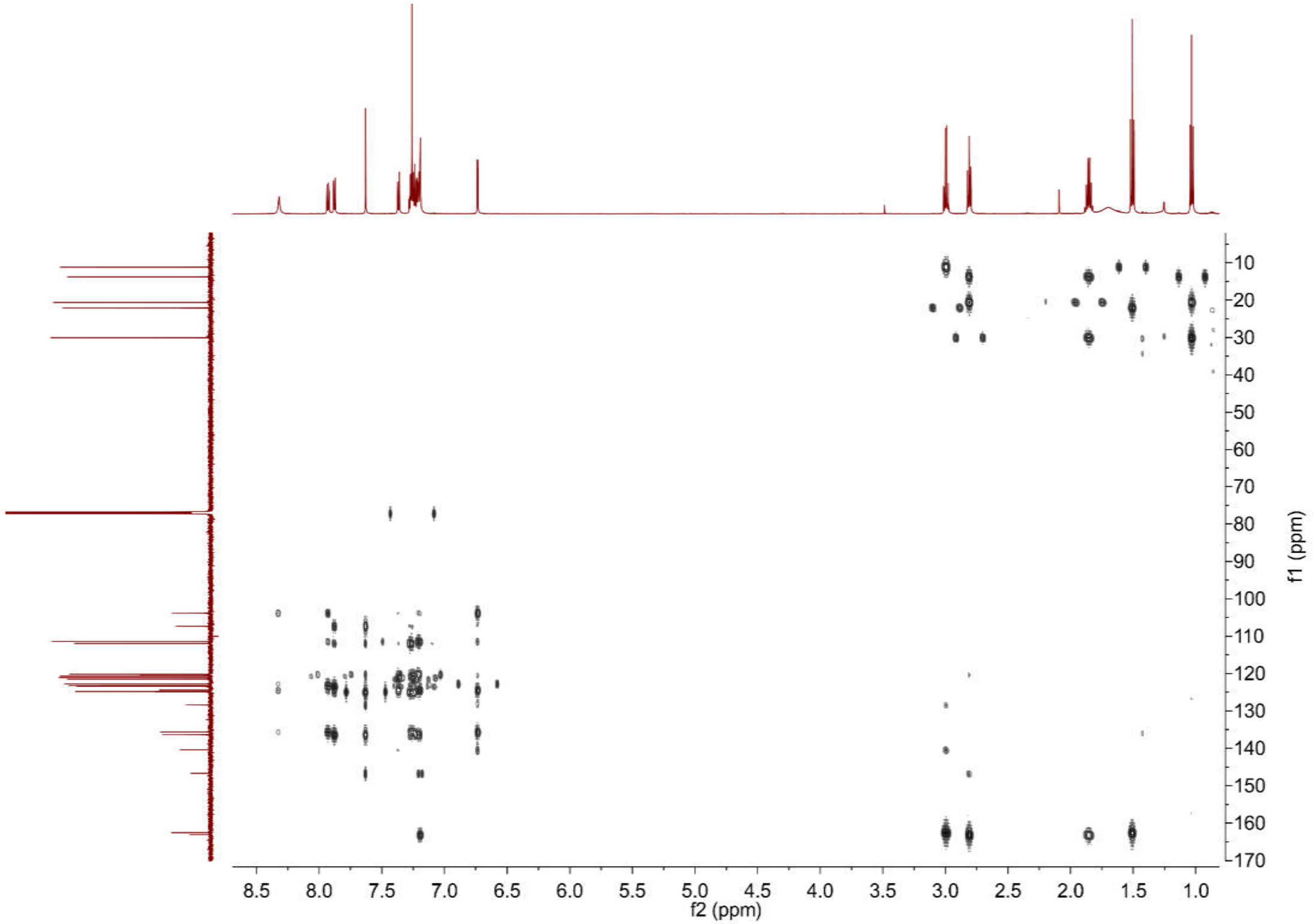

Figure S15. HMBC (600 MHz) spectrum of compound **2** in $CDCl_3$.

Qualitative Analysis Report

**Data Filename**	HSXC9371.d	**Sample Name**	HSXC9371
**Sample Type**	Sample	**Position**	P1-A1
**Instrument Name**	Instrument 1	**User Name**	
**Acq Method**	s-.m	**Acquired Time**	5/16/2019 1:51:09 PM
**IRM Calibration Status**	Success	**DA Method**	Default.m
**Comment**			

**Sample Group**		**Info.**
**Acquisition SW Version**	6200 series TOF/6500 series Q-TOF B.05.01 (B5125.2)	

User Spectra

Fragmentor Voltage	Collision Energy	Ionization Mode
135	0	ESI

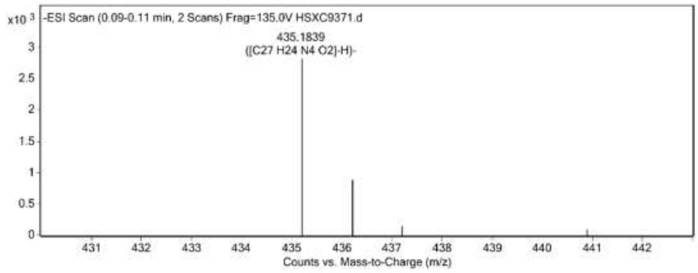

Peak List

m/z	z	Abund	Formula	Ion
61.9884		16416.02		
112.9854		1067.94		
130.966		2417.48		
146.9658		3900.64		
174.9556	1	2362.22		
435.1839	1	2825.02	C27 H24 N4 O2	(M-H)-
436.1874	1	899.05	C27 H24 N4 O2	(M-H)-
965.9994	1	3093.87		
982.9906	1	4884.5		
983.9939	1	1305.05		

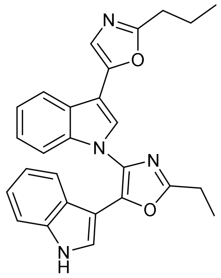

Formula Calculator Element Limits

Element	Min	Max
C	3	60
H	0	120
O	0	30
N	0	10

Formula Calculator Results

Formula	CalculatedMass	CalculatedMz	Mz	Diff. (mDa)	Diff. (ppm)	DBE
C27 H24 N4 O2	436.1899	435.1826	435.1839	-1.30	-2.99	18.0000

--- End Of Report ---

Figure S16. HRESIMS spectrum of compound **2**.

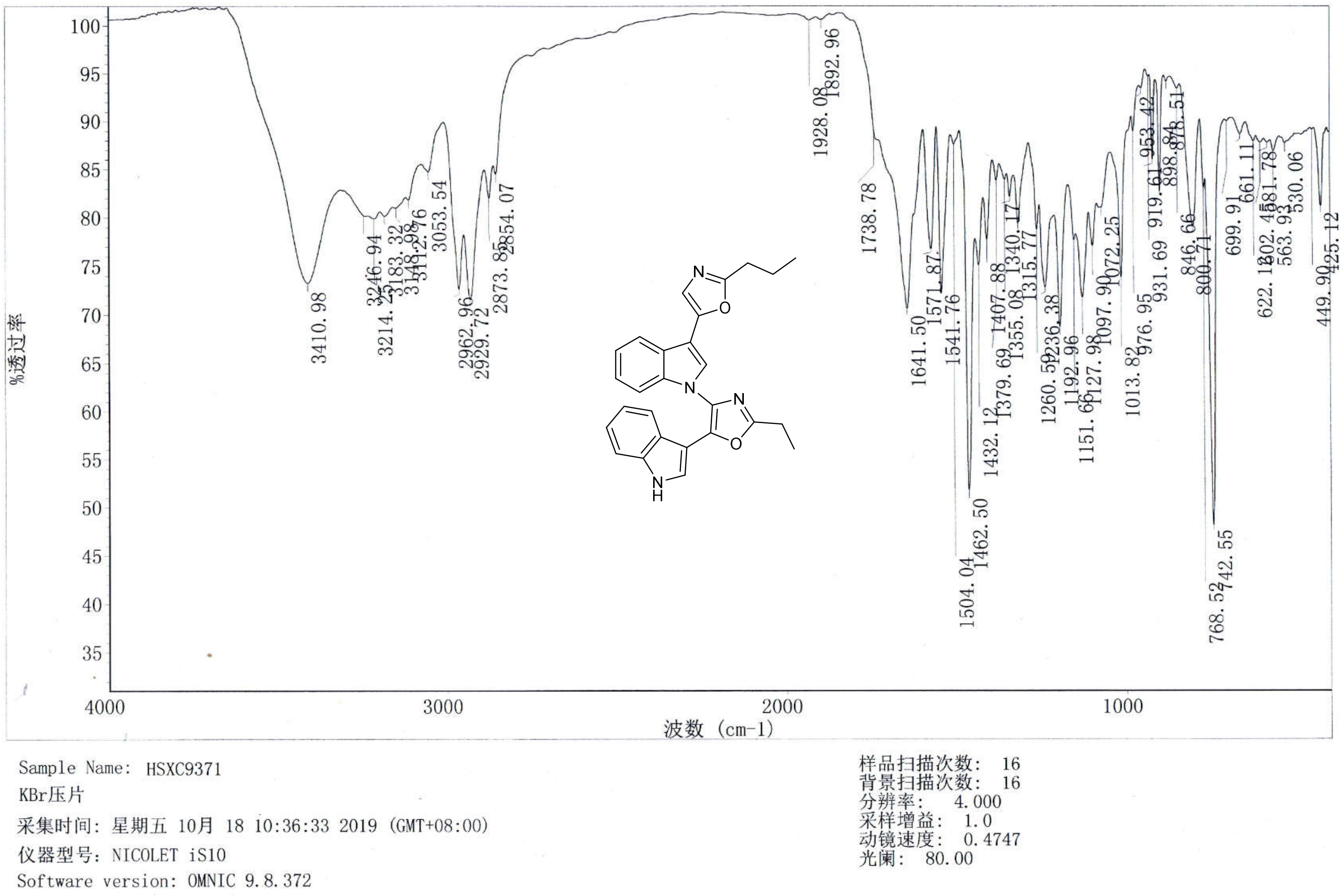

Figure S17. IR spectrum of compound **2**.

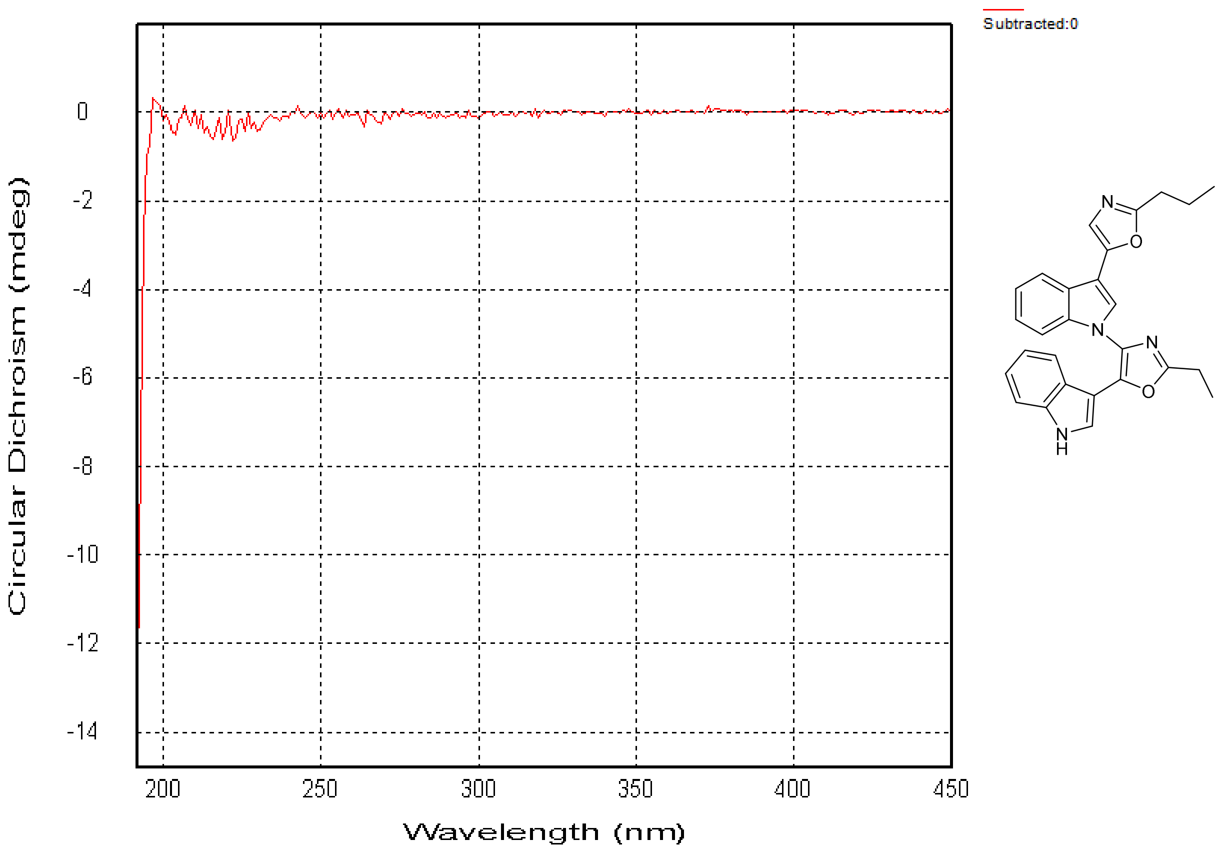

Figure S18. CD spectrum of compound **2**.

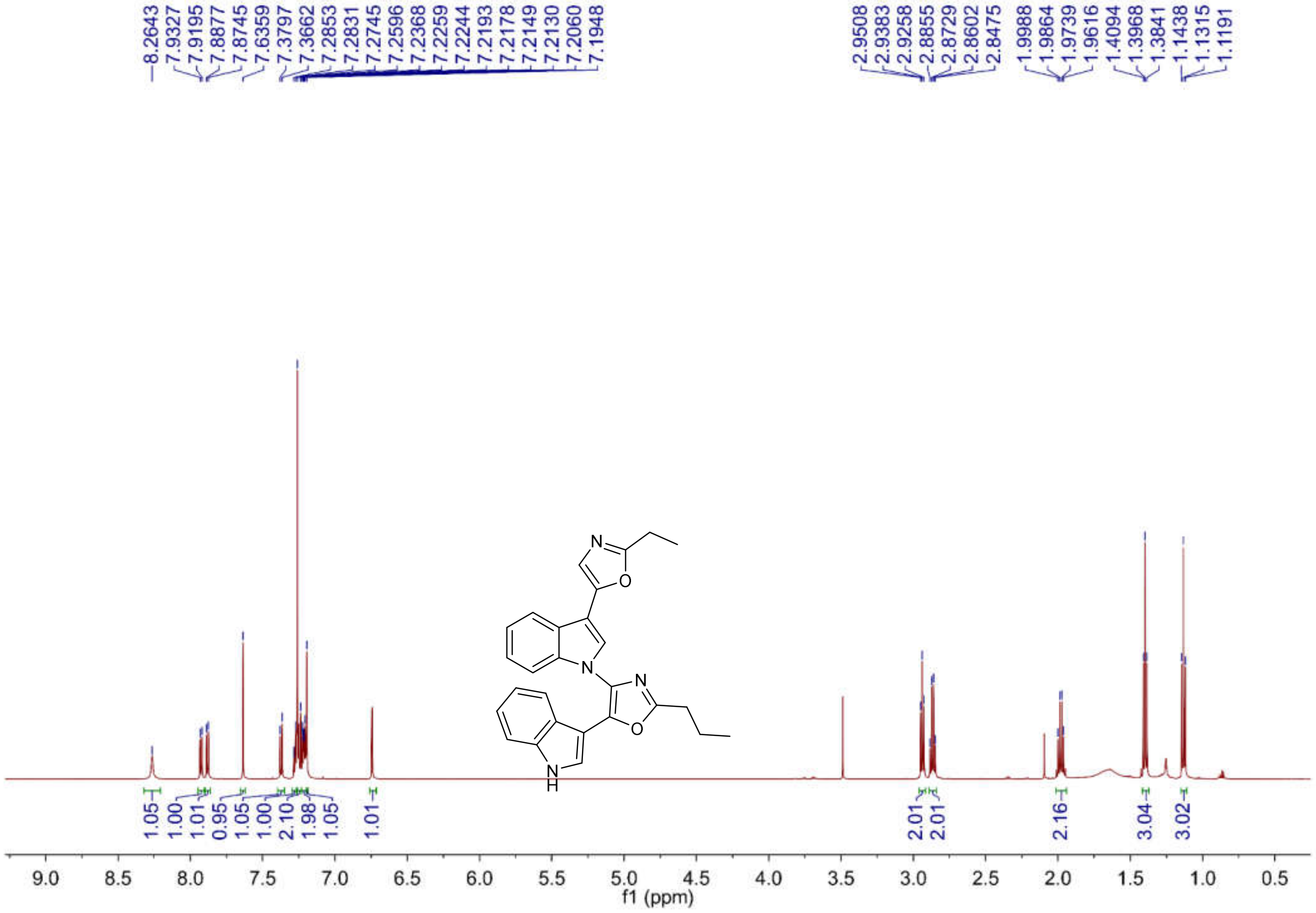

Figure S19. ^{1}H NMR (600 MHz) spectrum of compound **3** in $CDCl_3$.

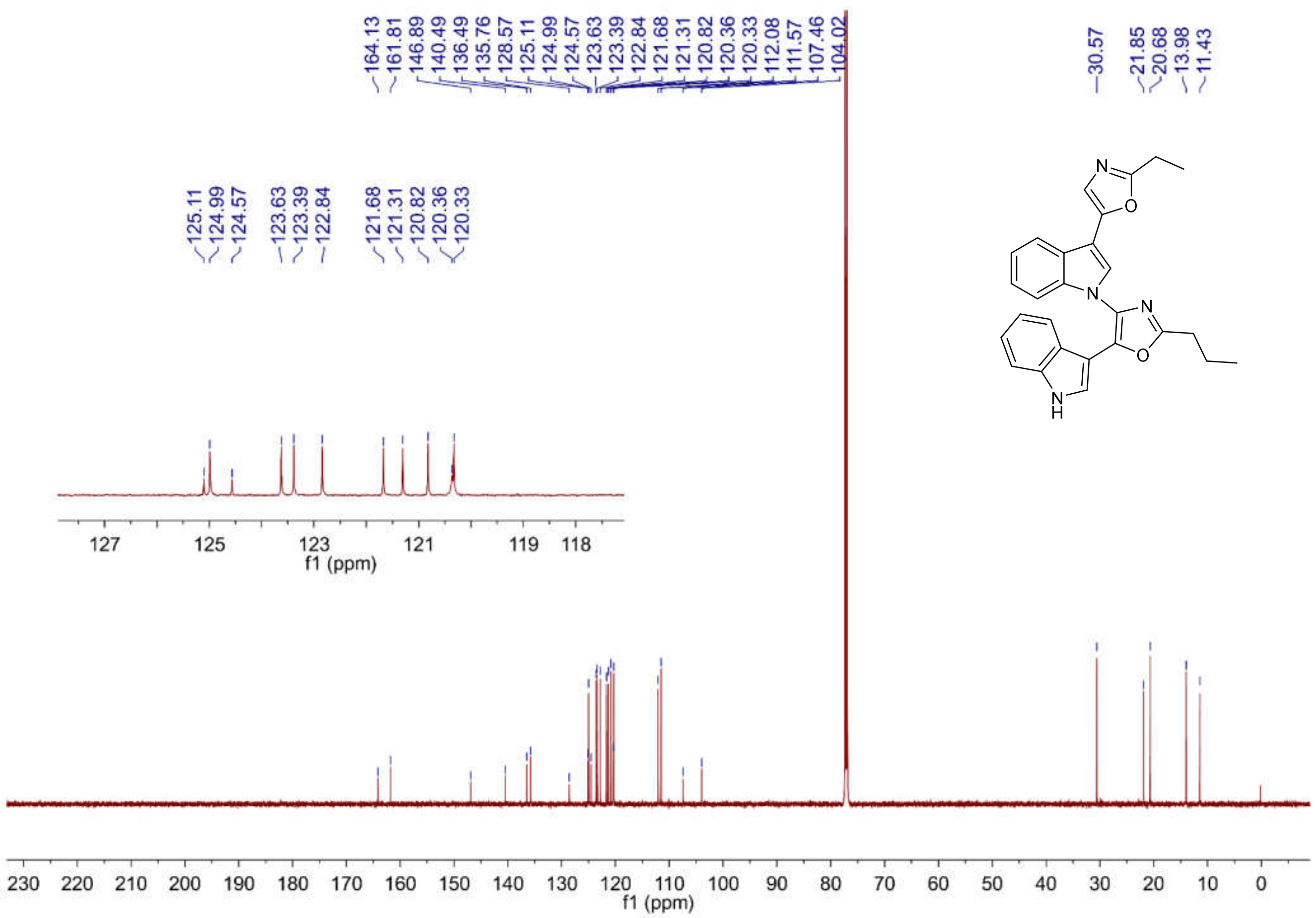

Figure S20. ^{13}C NMR (150 MHz) spectrum of compound **3** in $CDCl_3$.

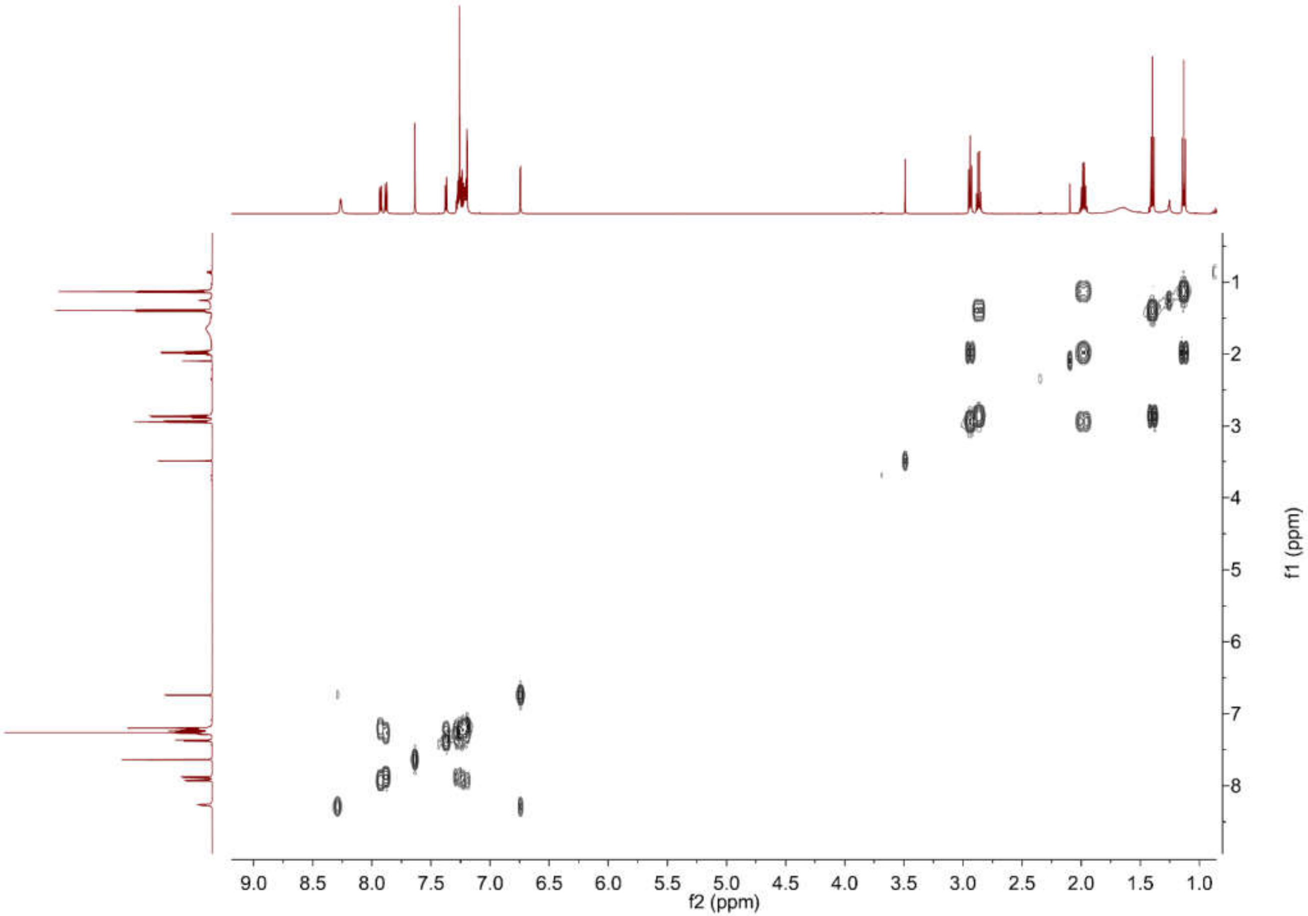

Figure S21. $^{1}H–^{1}H$ COSY (600 MHz) spectrum of compound **3** in $CDCl_3$.

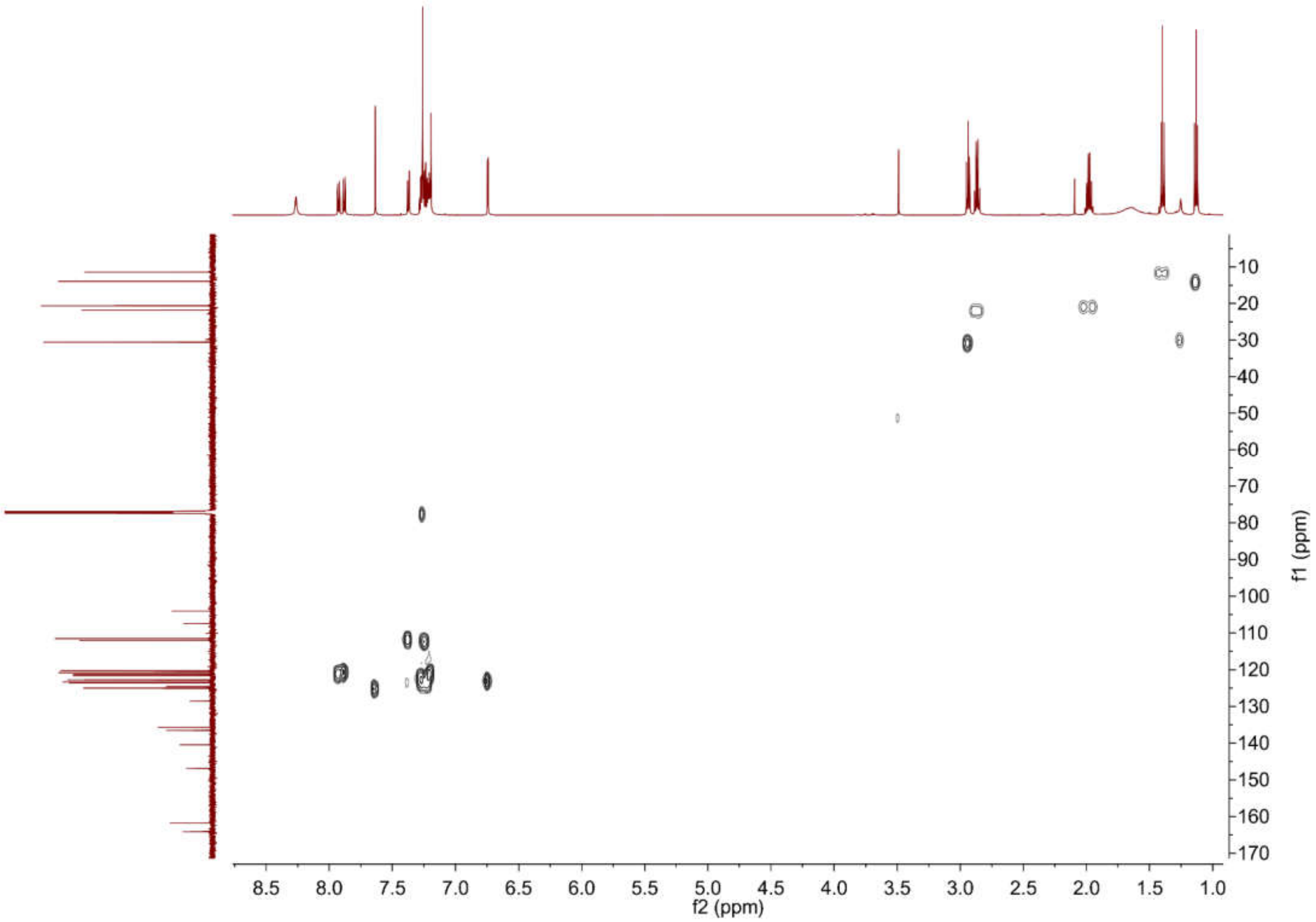

Figure S22. HSQC (600 MHz) spectrum of compound **3** in $CDCl_3$.

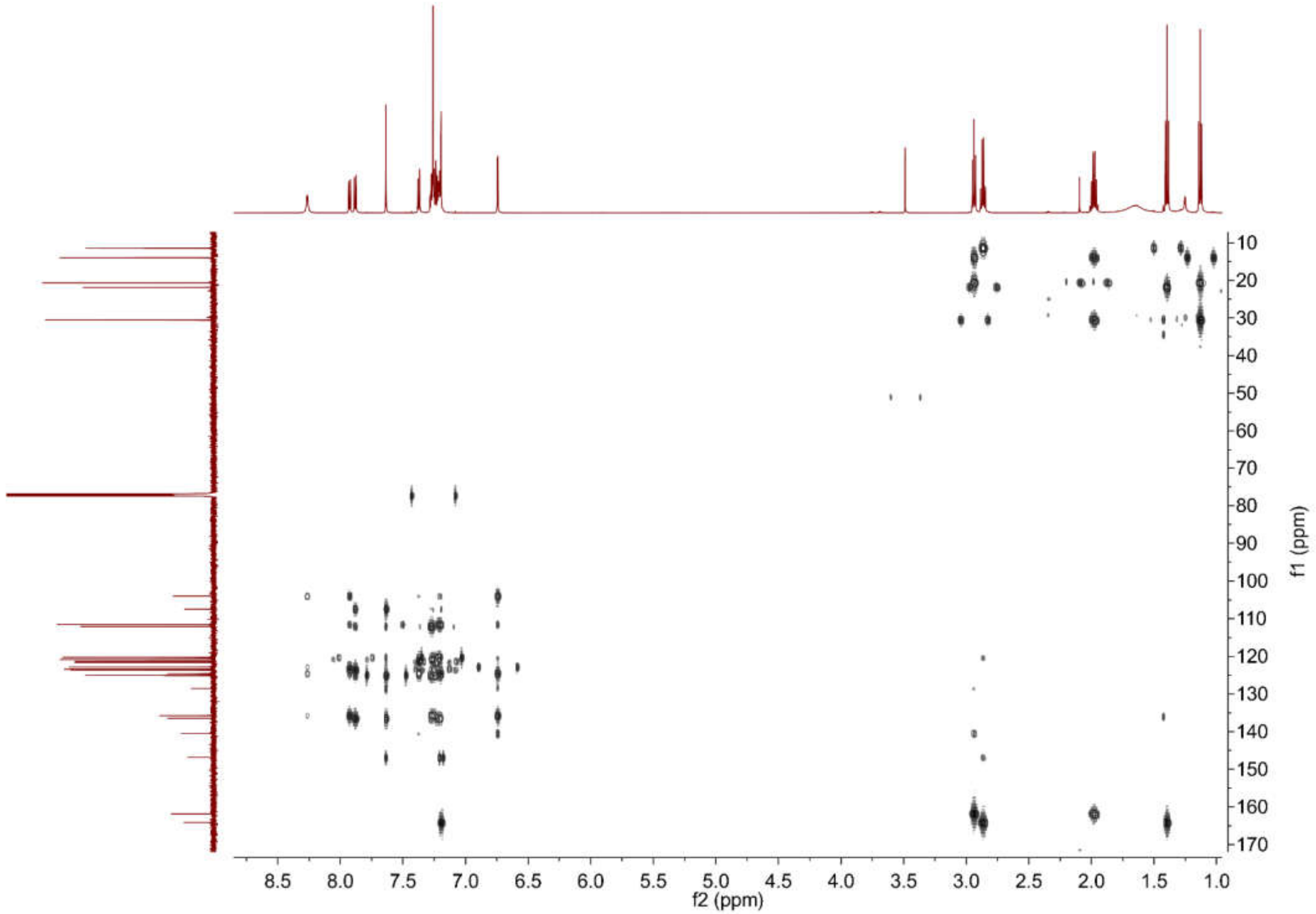

Figure S23. HMBC (600 MHz) spectrum of compound **3** in $CDCl_3$.

Qualitative Analysis Report

**Data Filename**	HSXC9381.d	**Sample Name**	HSXC9381
**Sample Type**	Sample	**Position**	P1-A2
**Instrument Name**	Instrument 1	**User Name**	
**Acq Method**	s-.m	**Acquired Time**	5/16/2019 1:52:20 PM
**IRM Calibration Status**	Success	**DA Method**	Default.m
**Comment**			

**Sample Group**		**Info.**
**Acquisition SW**	6200 series TOF/6500 series	
**Version**	Q-TOF B.05.01 (B5125.2)	

User Spectra

Fragmentor Voltage	Collision Energy	Ionization Mode
135	0	ESI

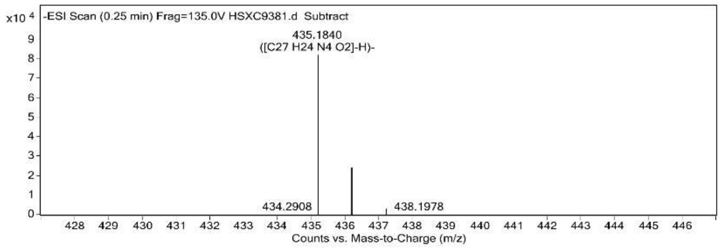

Peak List

m/z	z	Abund	Formula	Ion
61.9885	1	101314.23		
112.9855		7789.38		
211.0875	1	3459.17		
435.184	1	82482.02	C27 H24 N4 O2	(M-H)-
436.1869	1	24455.5	C27 H24 N4 O2	(M-H)-
437.1902	1	3249.07	C27 H24 N4 O2	(M-H)-
471.1612	1	11666.1		
472.1643	1	3754.37		
473.1595	1	3581.12		
966.0008	1	1419.69		

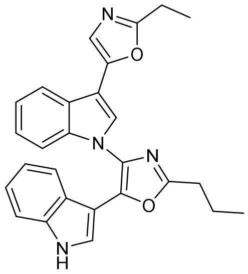

Formula Calculator Element Limits

Element	Min	Max
C	3	60
H	0	120
O	0	30
N	0	10

Formula Calculator Results

Formula	CalculatedMass	CalculatedMz	Mz	Diff. (mDa)	Diff. (ppm)	DBE
C27 H24 N4 O2	436.1899	435.1826	435.1840	-1.40	-3.22	18.0000

--- End Of Report ---

Figure S24. HRESIMS spectrum of compound **3**.

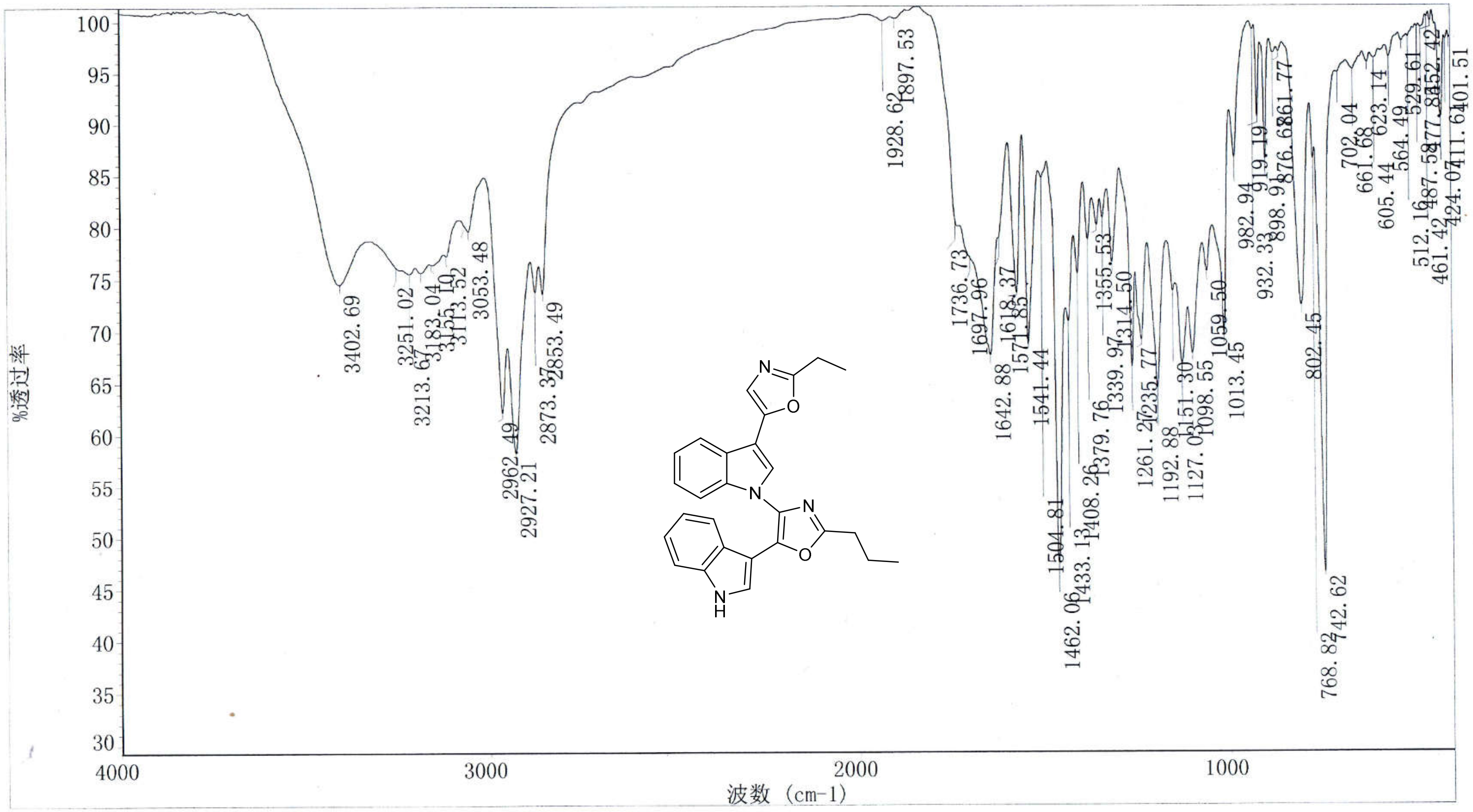

Sample Name: HSXC9381
KBr压片
采集时间: 星期五 10月 18 10:59:22 2019 (GMT+08:00)
仪器型号: NICOLET iS10
Software version: OMNIC 9.8.372

样品扫描次数: 16
背景扫描次数: 16
分辨率: 4.000
采样增益: 1.0
动镜速度: 0.4747
光阑: 80.00

Figure S25. IR spectrum of compound **3**.

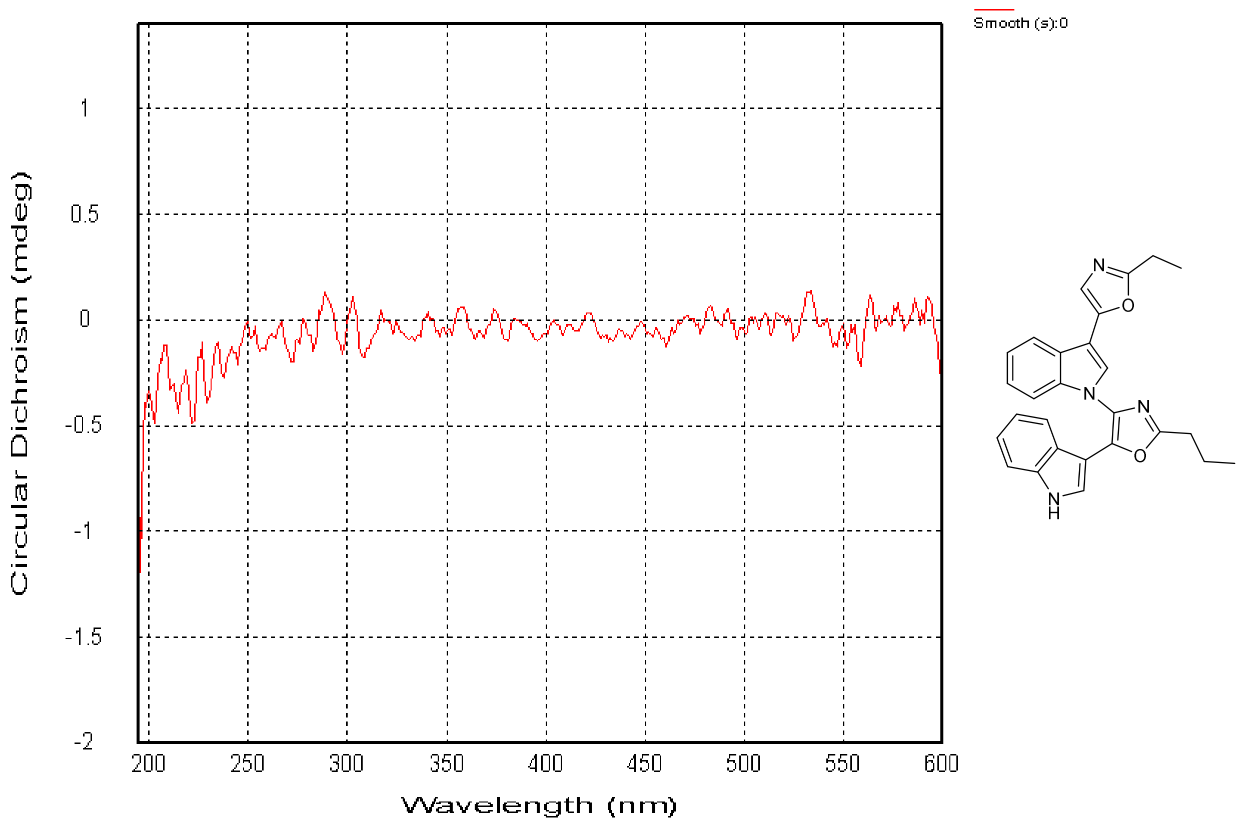

Figure S26. CD spectrum of compound **3**.

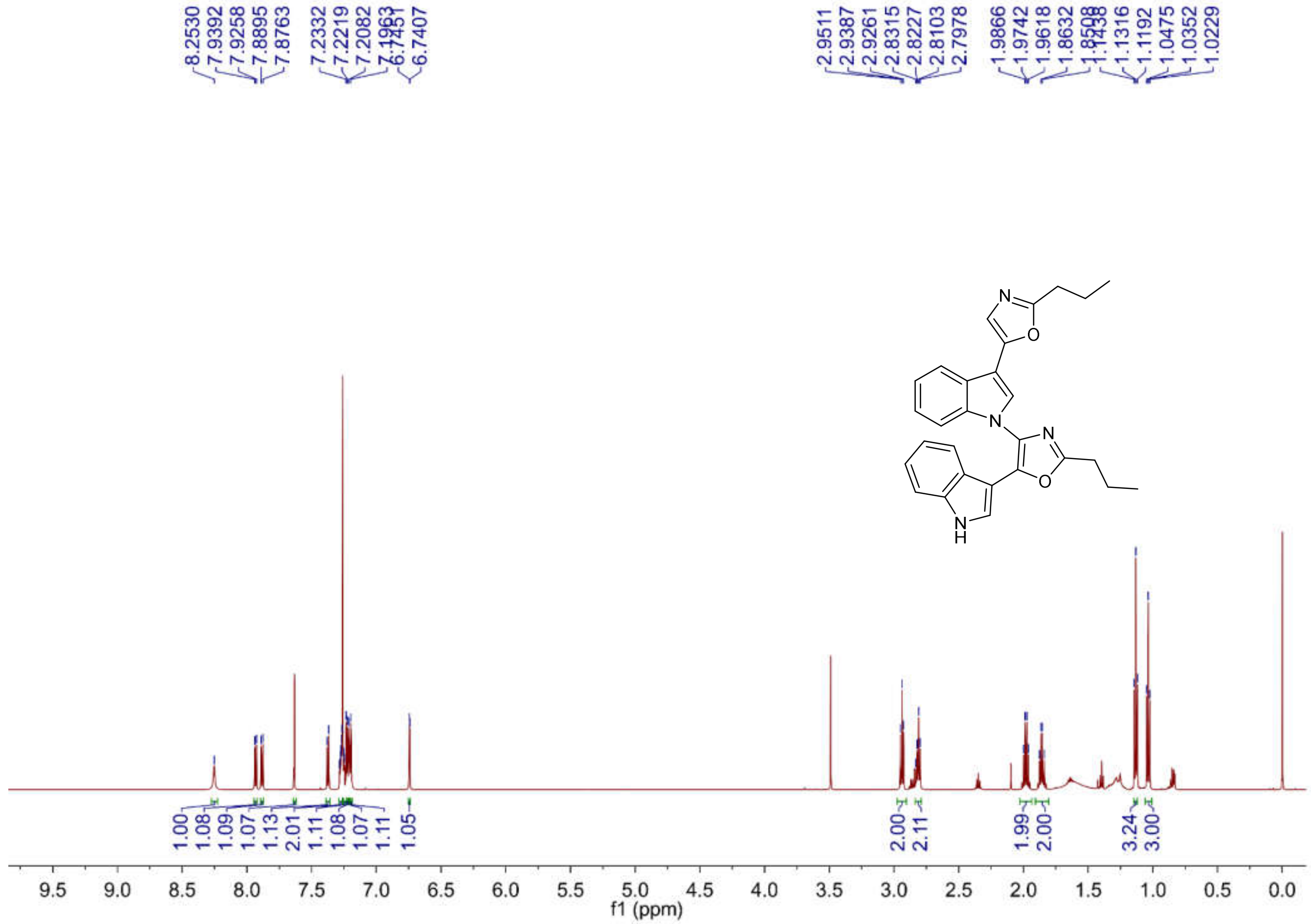

Figure S27. ^{1}H NMR (600 MHz) spectrum of compound **4** in $CDCl_3$.

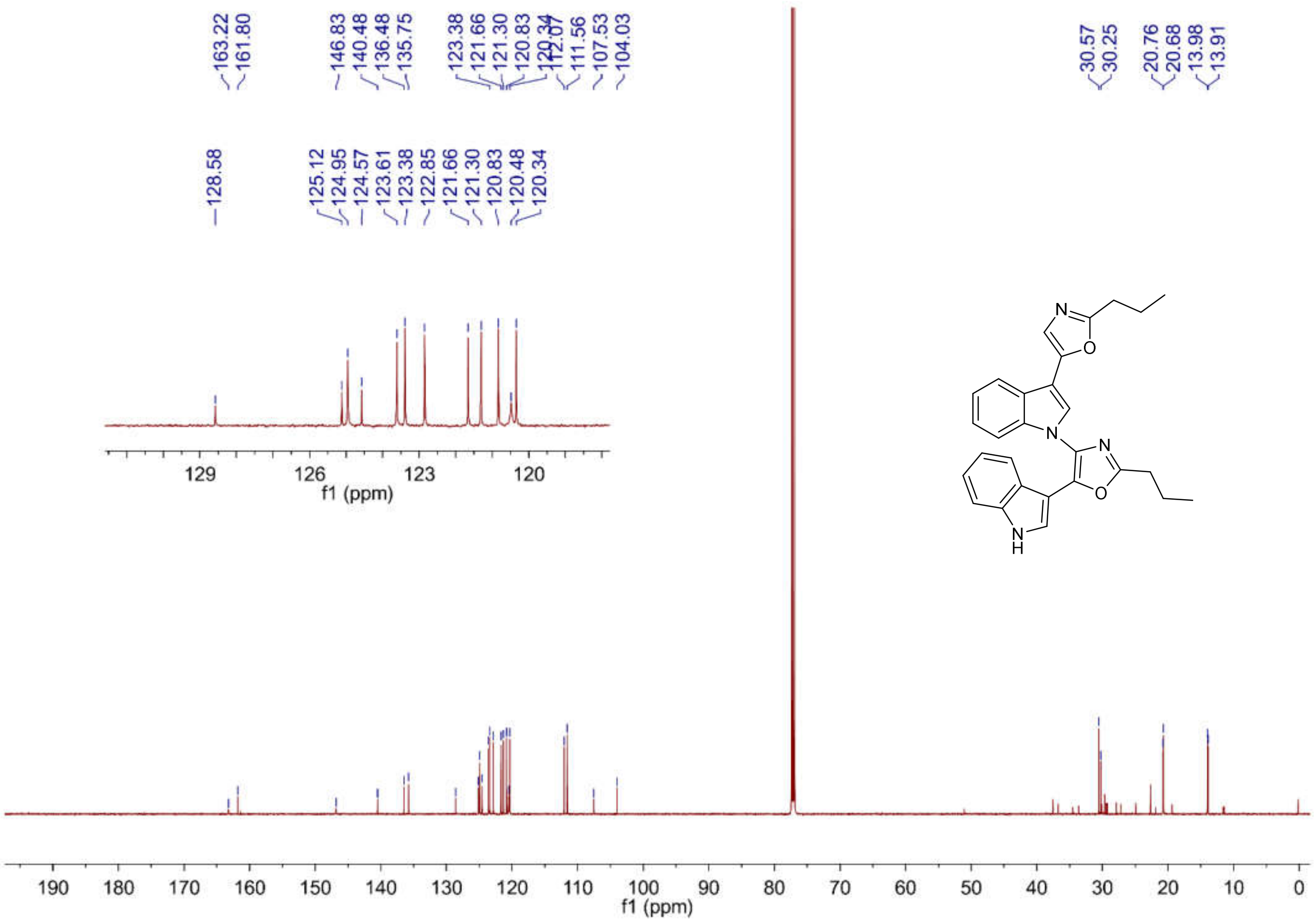

Figure S28. ^{13}C NMR (150 MHz) spectrum of compound **4** in $CDCl_3$.

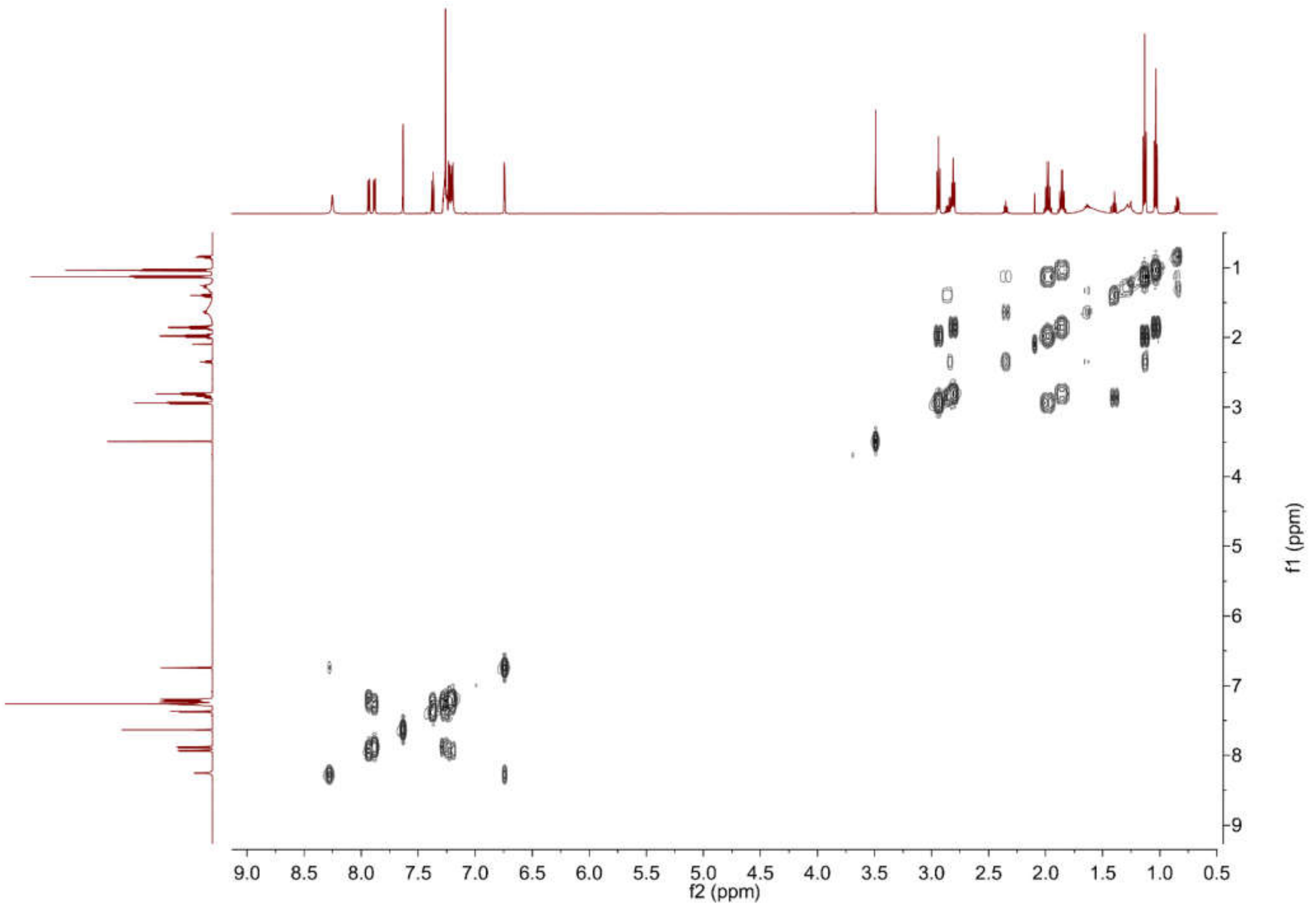

Figure S29. $^1H-^1H$ COSY (600 MHz) spectrum of compound **4** in $CDCl_3$.

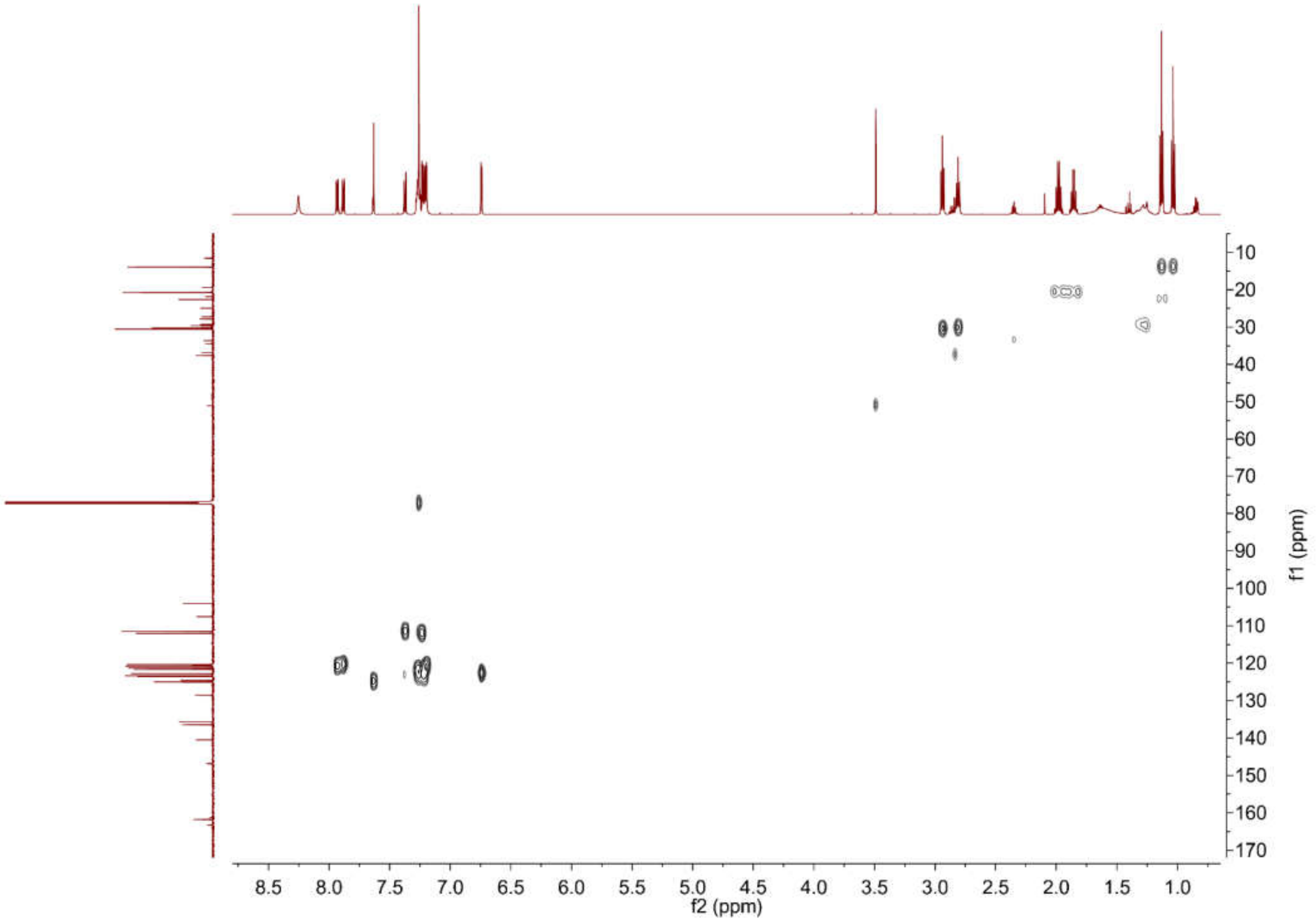

Figure S30. HSQC (600 MHz) spectrum of compound **4** in $CDCl_3$.

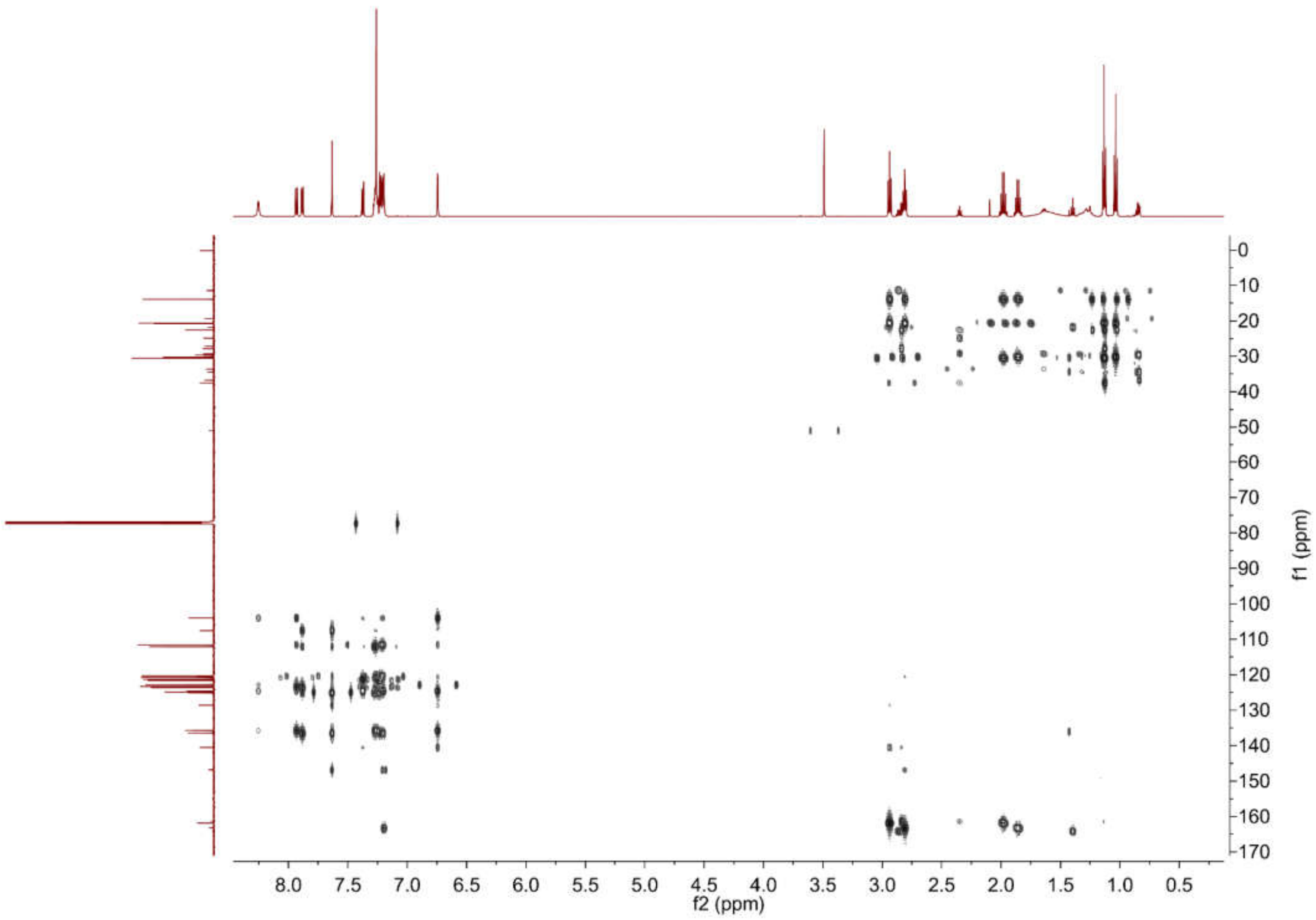

Figure S31. HMBC (600 MHz) spectrum of compound **4** in $CDCl_3$.

Qualitative Analysis Report

**Data Filename**	HSXC9401.d	**Sample Name**	HSXC9401
**Sample Type**	Sample	**Position**	P1-A3
**Instrument Name**	Instrument 1	**User Name**	
**Acq Method**	s-.m	**Acquired Time**	5/16/2019 1:53:31 PM
**IRM Calibration Status**	Success	**DA Method**	Default.m
**Comment**			

**Sample Group**		**Info.**
**Acquisition SW**	6200 series TOF/6500 series	
**Version**	Q-TOF B.05.01 (B5125.2)	

User Spectra

Fragmentor Voltage	Collision Energy	Ionization Mode
135	0	ESI

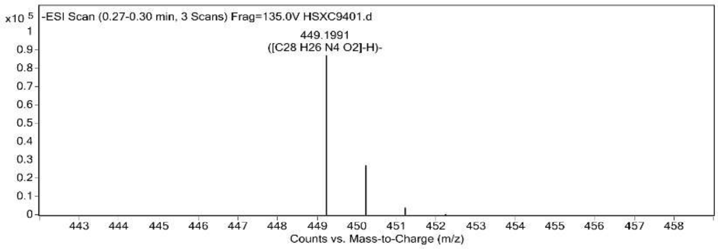

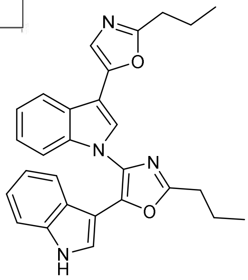

Peak List

m/z	z	Abund	Formula	Ion
61.9883		150617.39		
112.9852		9761.9		
449.1991	1	87511.09	C28 H26 N4 O2	(M-H)-
450.2021	1	27671.96	C28 H26 N4 O2	(M-H)-
451.2054	1	4453.44	C28 H26 N4 O2	(M-H)-
485.1761	1	13296		
486.1791	1	4193.23		
487.1752	1	4243.07		
965.998	1	5177.63		
982.9885	1	6930.86		

Formula Calculator Element Limits

Element	Min	Max
C	3	60
H	0	120
O	0	30
N	0	10

Formula Calculator Results

Formula	CalculatedMass	CalculatedMz	Mz	Diff. (mDa)	Diff. (ppm)	DBE
C28 H26 N4 O2	450.2056	449.1983	449.1991	-0.80	-1.78	18.0000

--- End Of Report ---

Figure S32. HRESIMS spectrum of compound **4**.

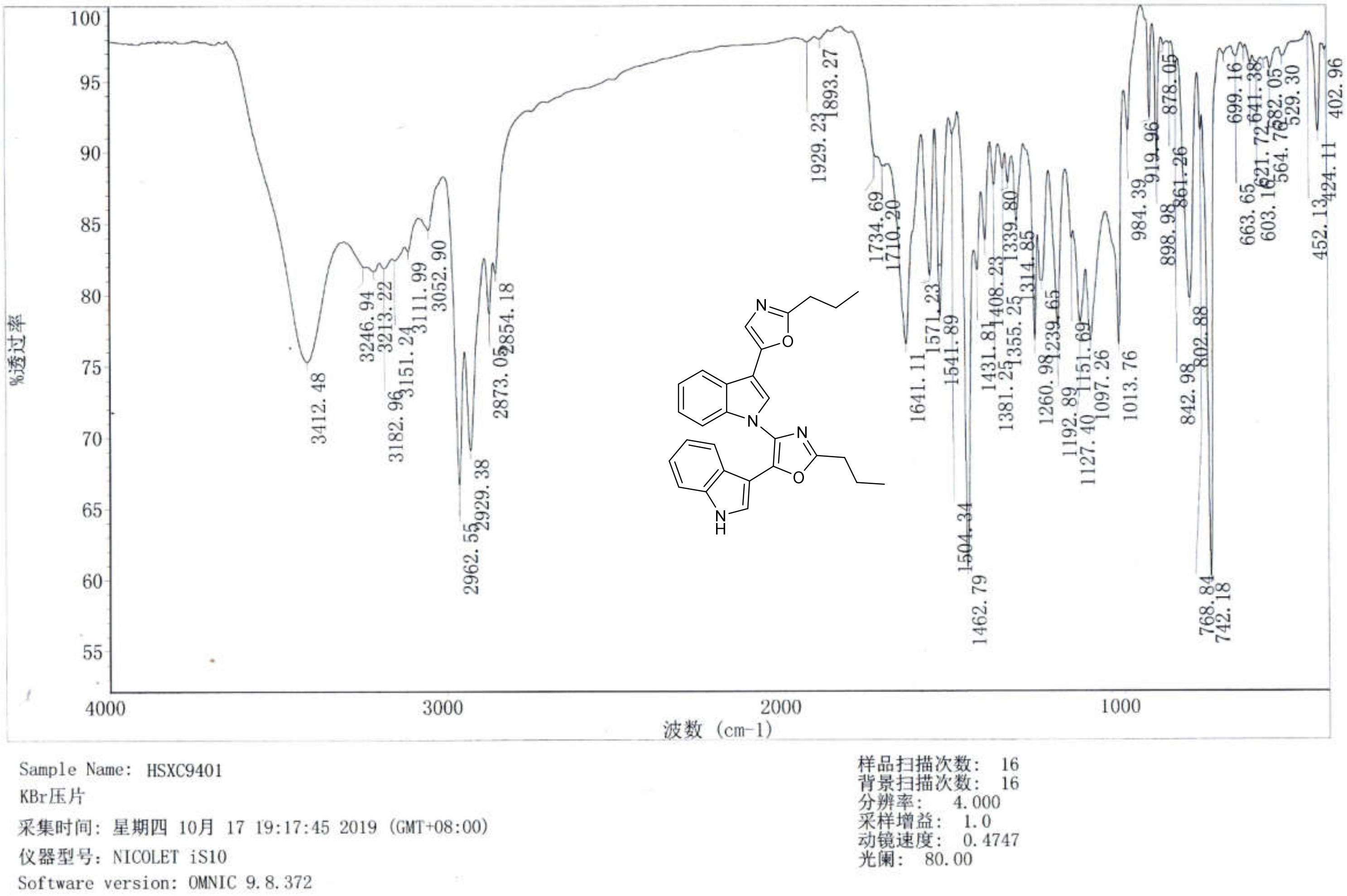

Figure S33. IR spectrum of compound **4**.

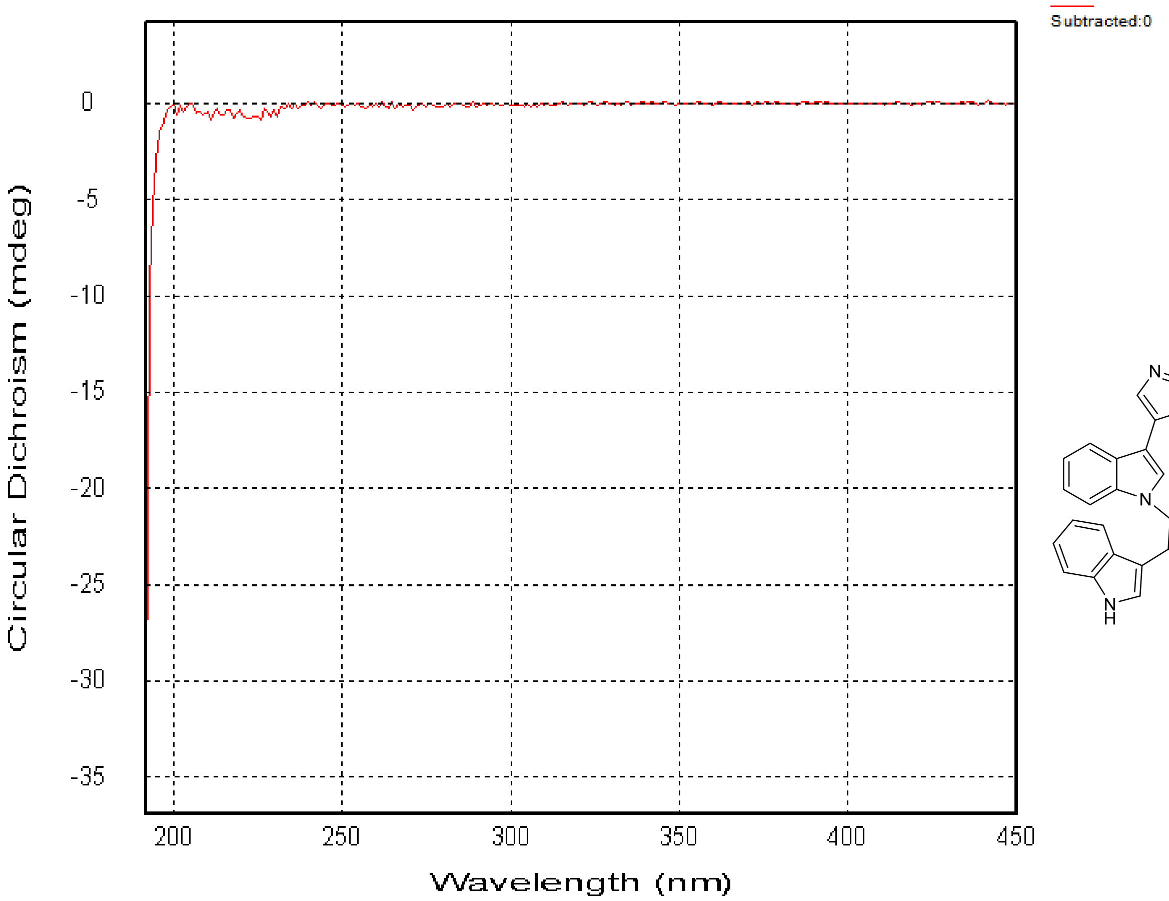

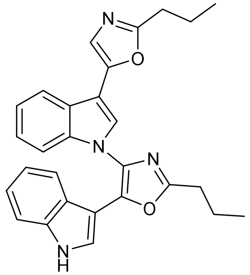

Figure S34. CD spectrum of compound **4**.

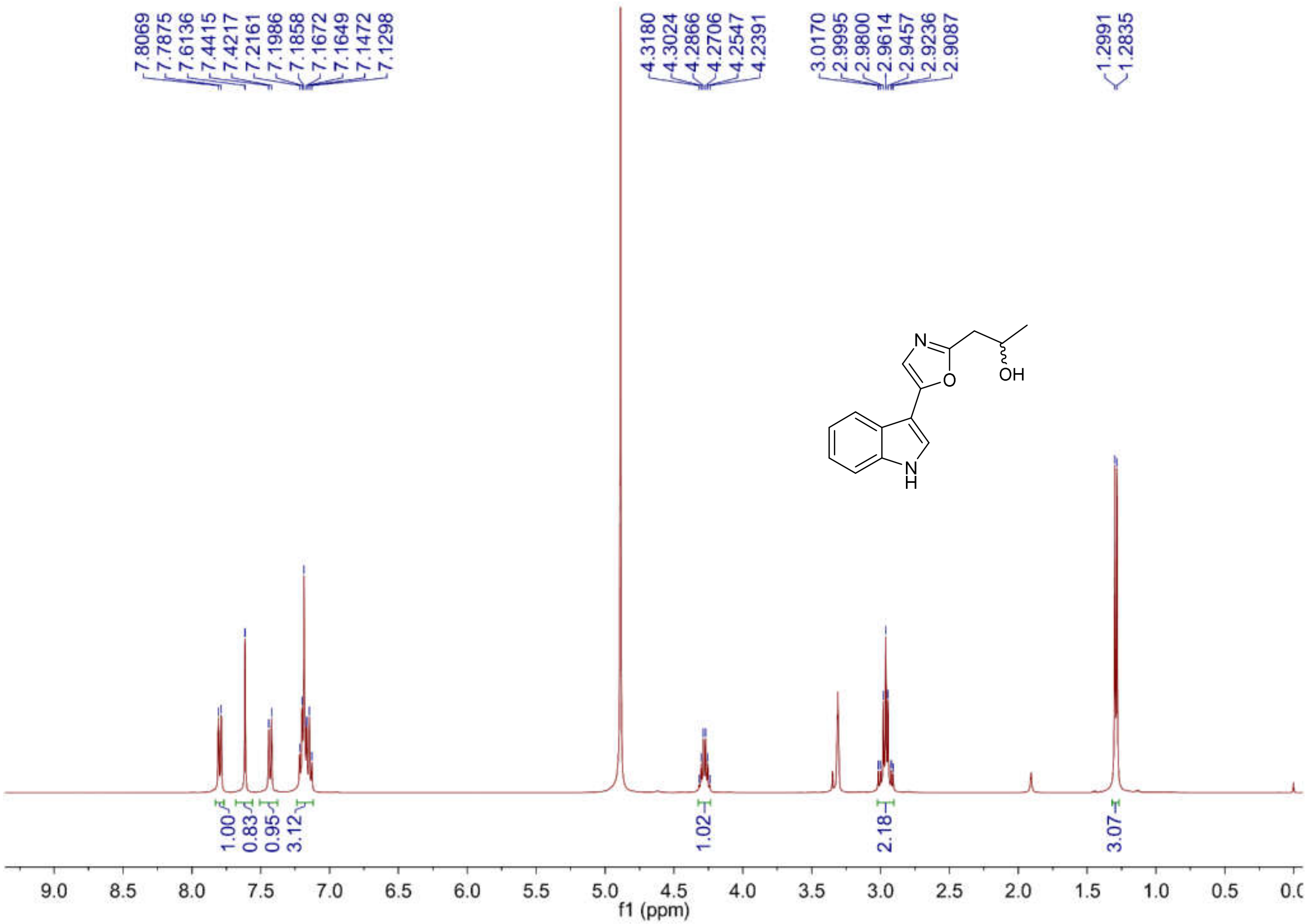

Figure S35. ^{1}H NMR (600 MHz) spectrum of compound **5** in methanol-d_4.

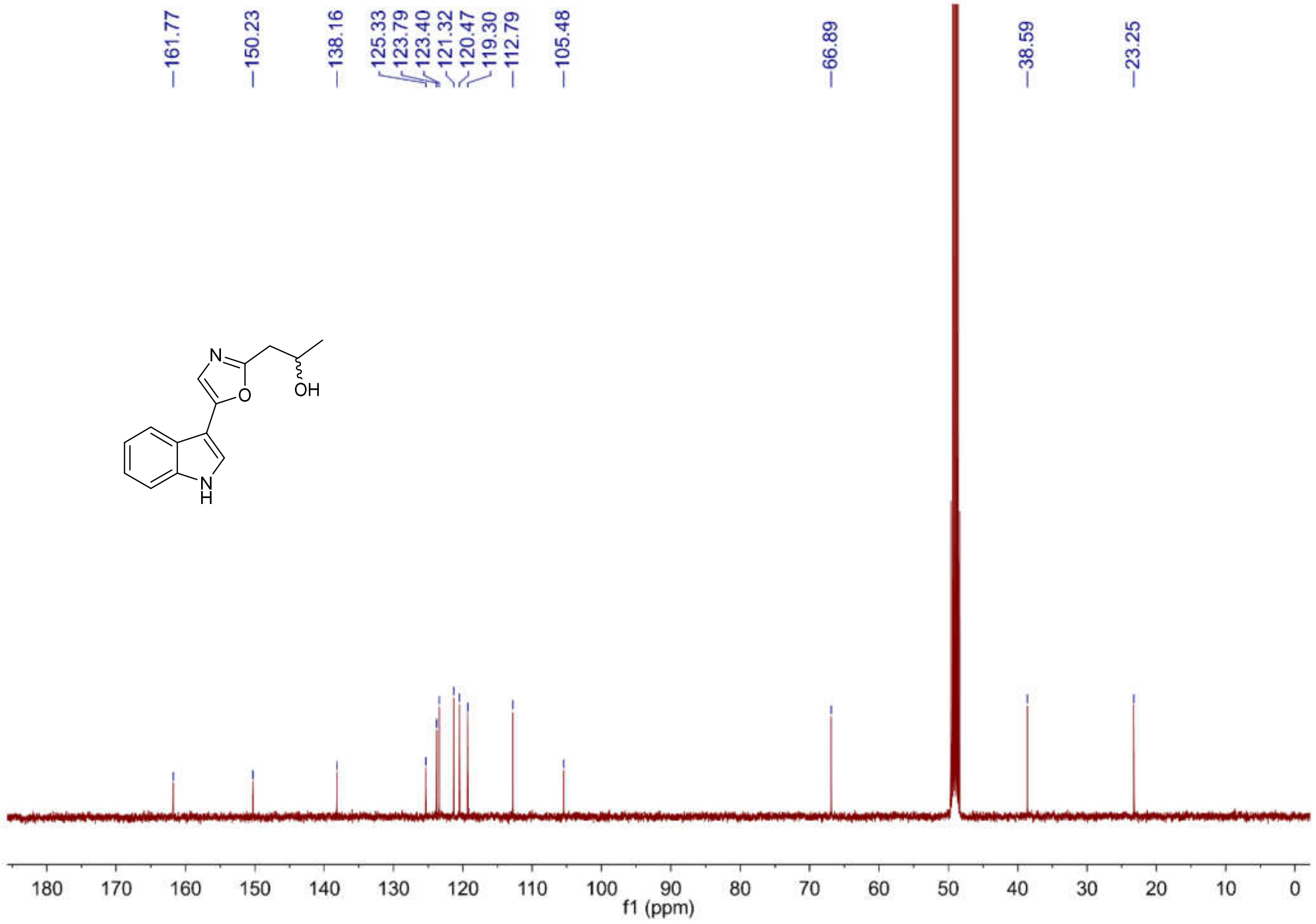

Figure S36. ^{13}C NMR (150 MHz) spectrum of compound **5** in methanol-d_4.

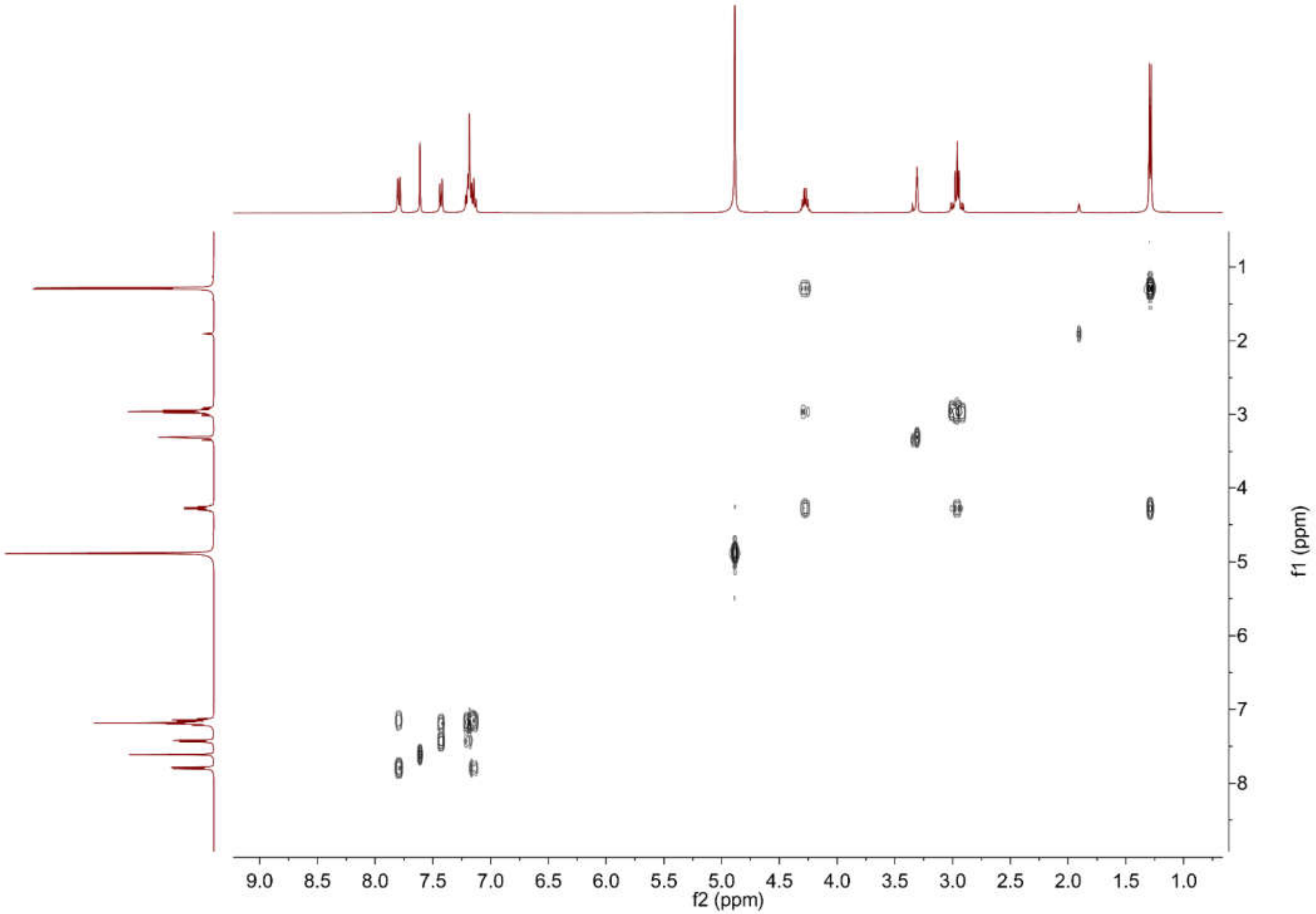

Figure S37. $^1H-^1H$ COSY (600 MHz) spectrum of compound **5** in methanol-d_4.

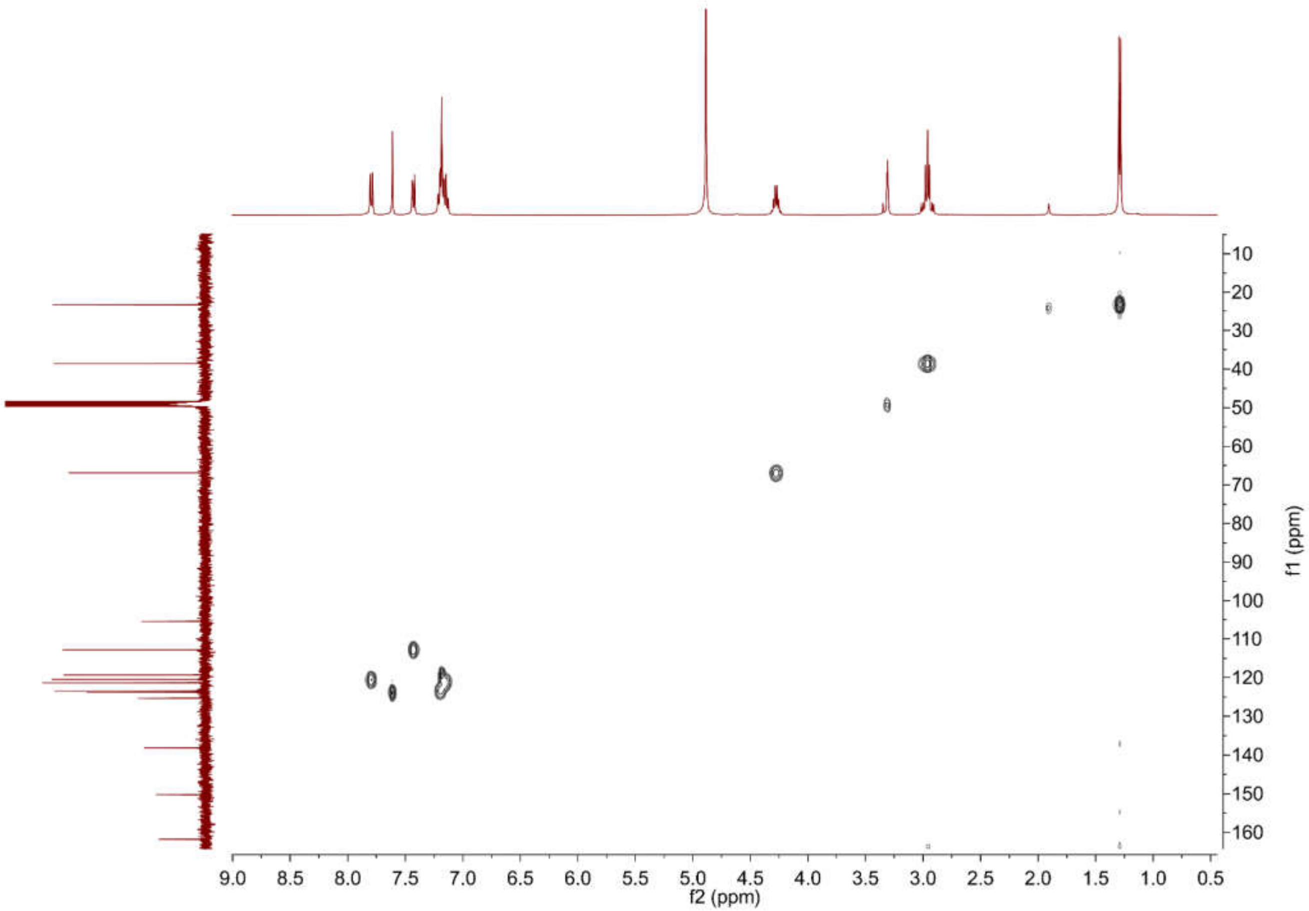

Figure S38. HSQC (600 MHz) spectrum of compound **5** in methanol-d_4.

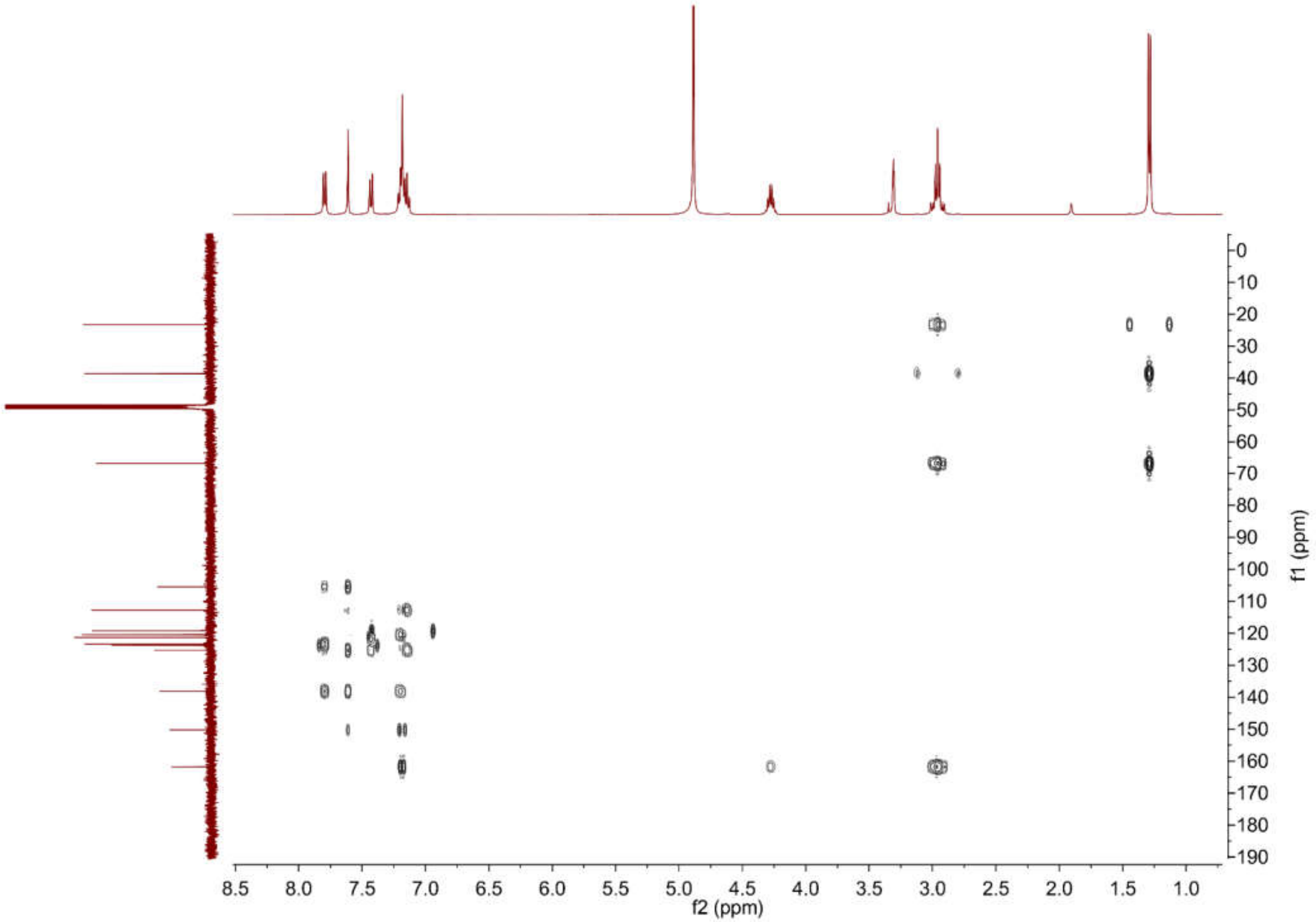

Figure S39. HMBC (600 MHz) spectrum of compound **5** in methanol-d_4

Qualitative Analysis Report

**Data Filename**	HSXC932.d	**Sample Name**	HSXC932
**Sample Type**	Sample	**Position**	P1-A6
**Instrument Name**	Instrument 1	**User Name**	
**Acq Method**	s.m	**Acquired Time**	4/11/2019 9:40:50 AM
**IRM Calibration Status**	Success	**DA Method**	Default.m
**Comment**			

**Sample Group**		**Info.**
**Acquisition SW**	6200 series TOF/6500 series	
**Version**	Q-TOF B.05.01 (B5125.2)	

User Spectra

Fragmentor Voltage	Collision Energy	Ionization Mode
135	0	ESI

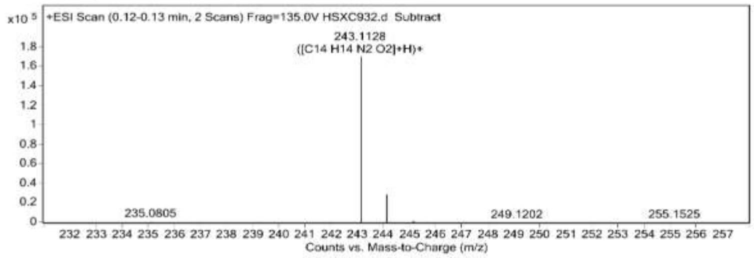

Peak List

m/z	z	Abund	Formula	Ion
102.1277		2333.24		
225.1023	1	9347.92		
226.1061	1	1712.77		
243.1128	1	170024.94	C14 H14 N2 O2	(M+H)+
244.116	1	29051.45	C14 H14 N2 O2	(M+H)+
245.1185	1	2376.75	C14 H14 N2 O2	(M+H)+
265.0948	1	104560.63		
266.0978	1	15578.21		
505.1847	1	1739.44		
507.2003	1	4994.41		

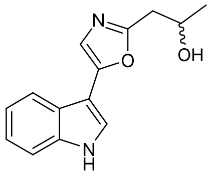

Formula Calculator Element Limits

Element	Min	Max
C	3	60
H	0	120
O	0	10
N	0	5
S	0	3

Formula Calculator Results

Formula	CalculatedMass	CalculatedMz	Mz	Diff. (mDa)	Diff. (ppm)	DBE
C14 H14 N2 O2	242.1055	243.1128	243.1128	0.00	0.00	9.0000

--- End Of Report ---

Figure S40. HRESIMS spectrum of compound **5**.

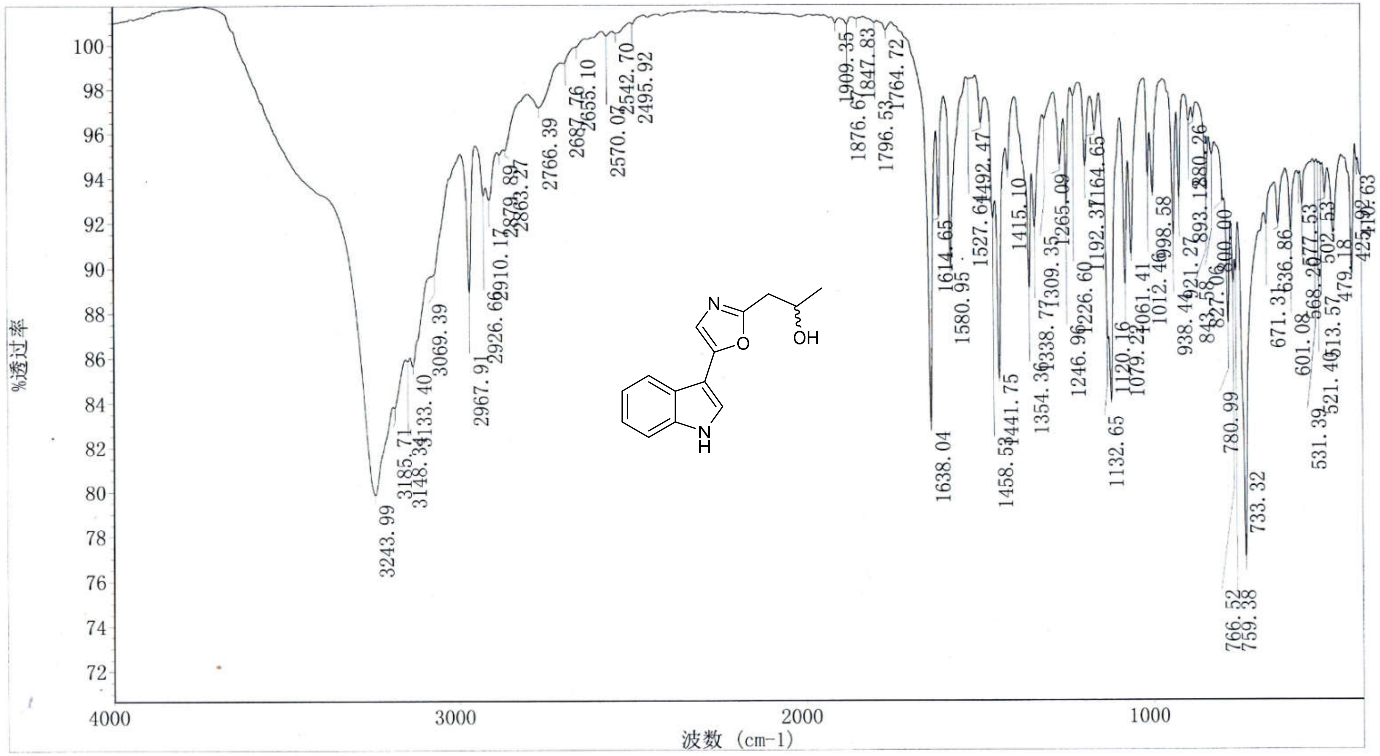

Sample Name: HSXC932
KBr压片
采集时间: 星期四 10月 17 19:43:36 2019 (GMT+08:00)
仪器型号: NICOLET iS10
Software version: OMNIC 9.8.372

样品扫描次数: 16
背景扫描次数: 16
分辨率: 4.000
采样增益: 1.0
动镜速度: 0.4747
光阑: 80.00

Figure S41. IR spectrum of compound **5**

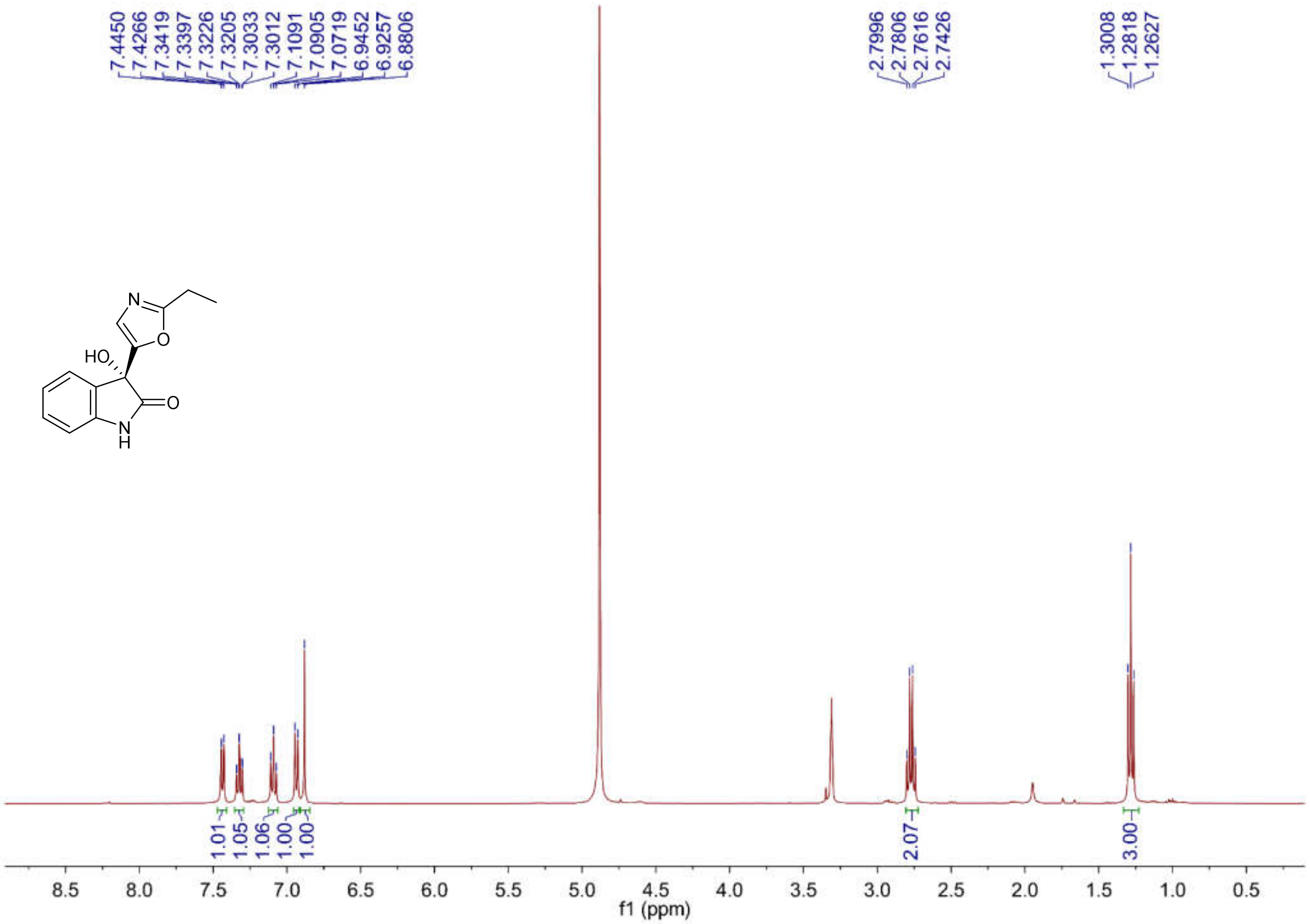

Figure S42. ^{1}H NMR (600 MHz) spectrum of compound **6** in methanol-d_4.

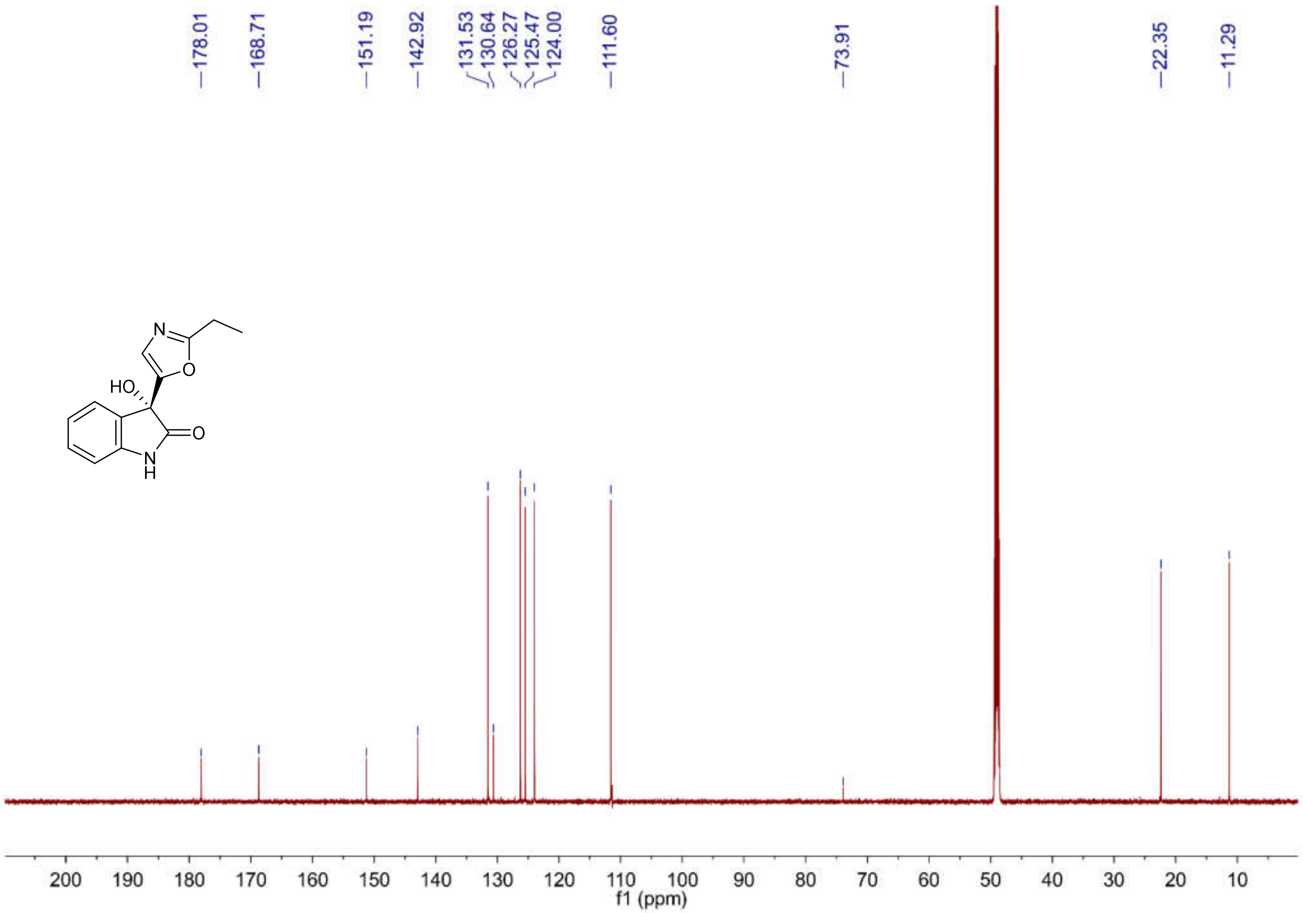

Figure S43. ^{13}C NMR (150 MHz) spectrum of compound **6** in methanol-d_4.

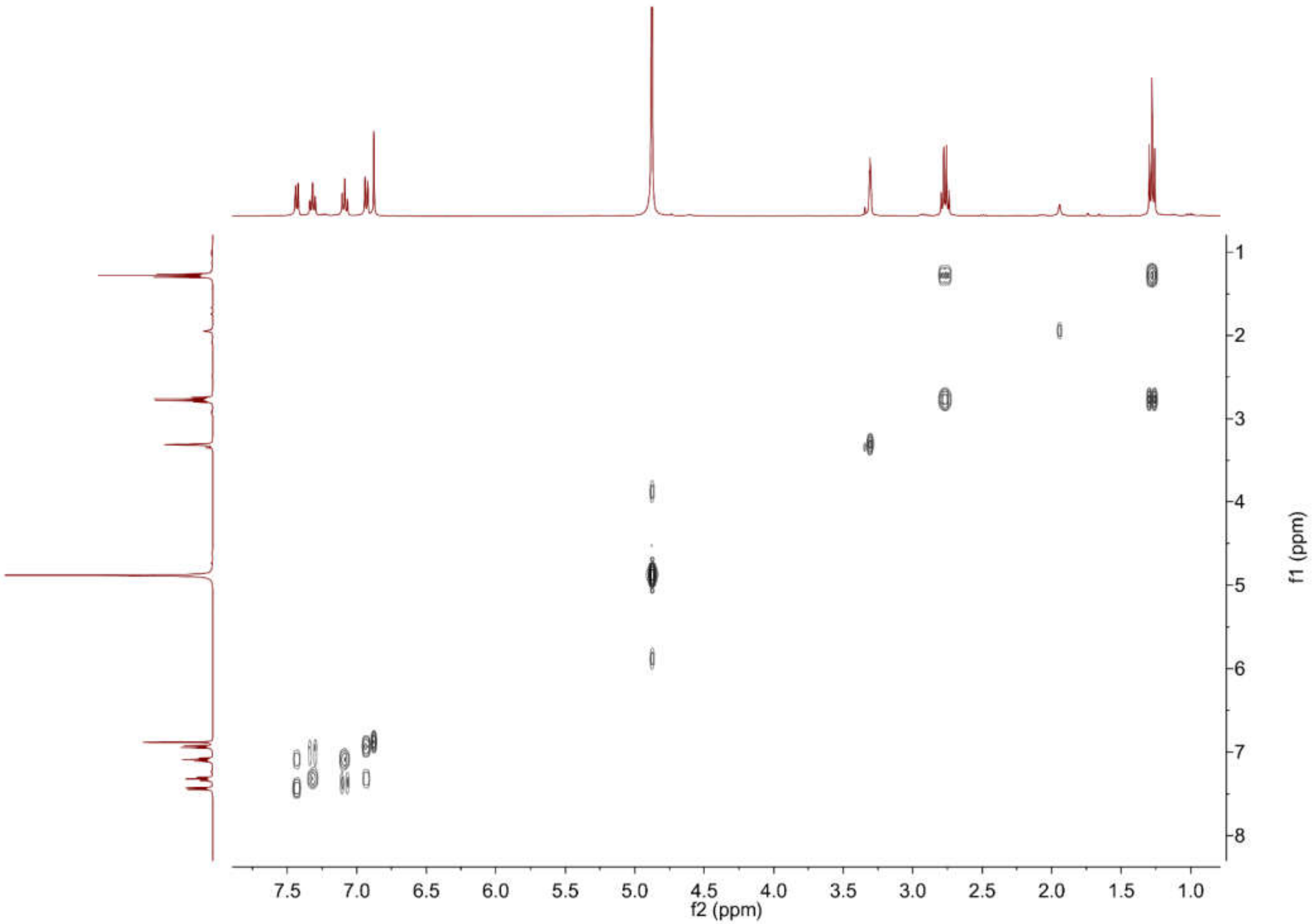

Figure S44. $^{1}H-^{1}H$ COSY (600 MHz) spectrum of compound **6** in methanol-d_4.

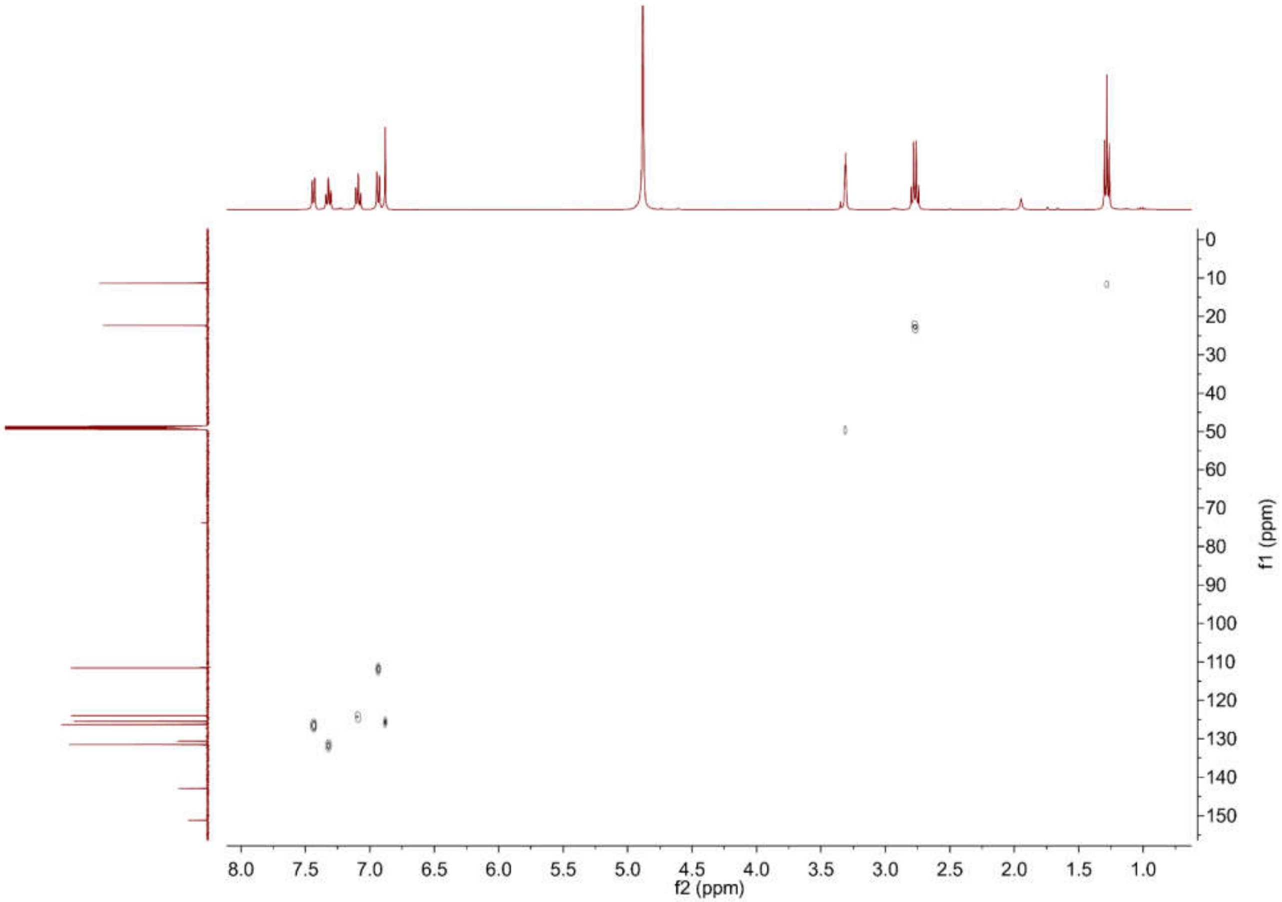

Figure S45. HSQC (600 MHz) spectrum of compound **6** in methanol-d_4.

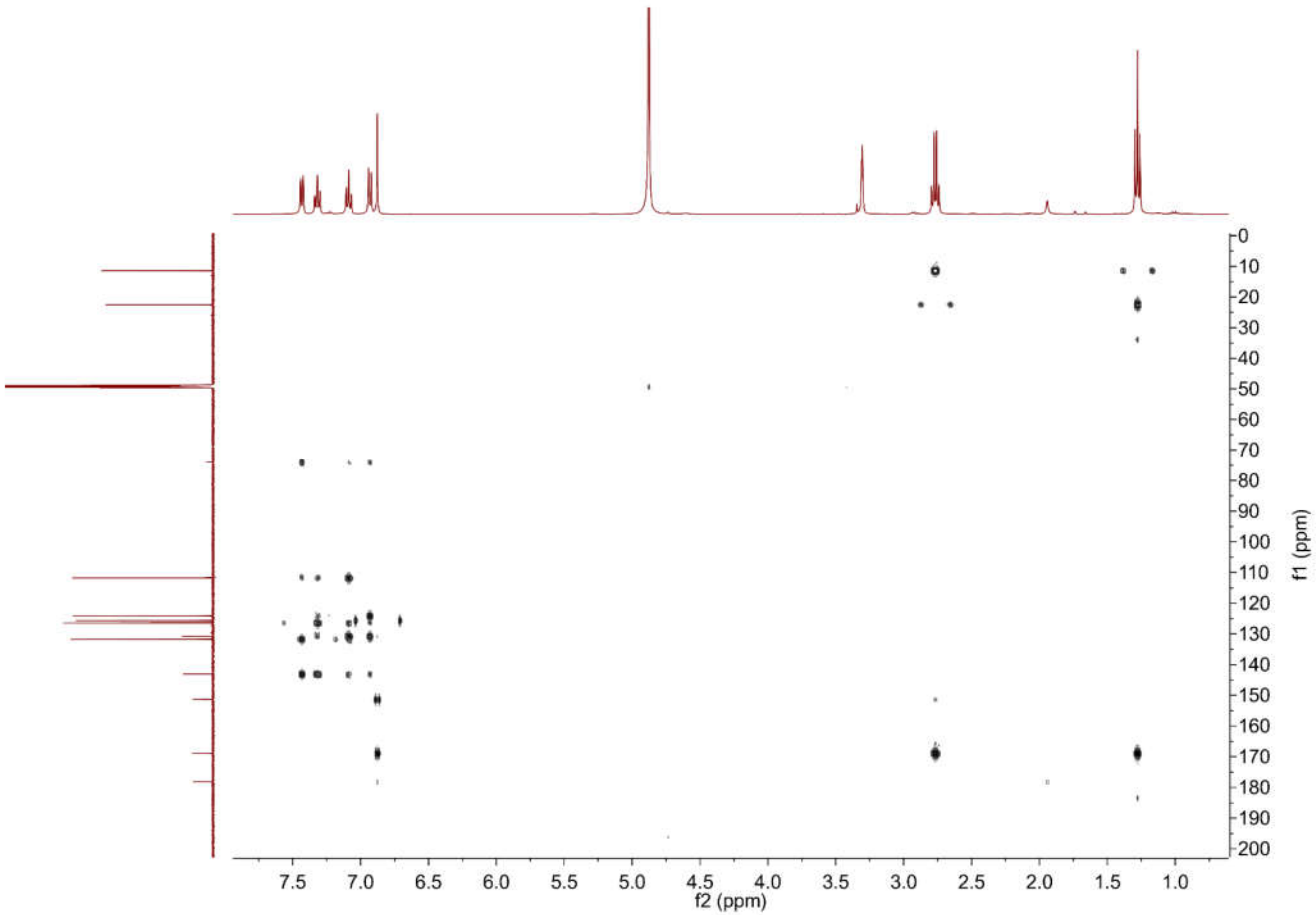

Figure S46. HMBC (600 MHz) spectrum of compound **6** in methanol-d_4.

Qualitative Analysis Report

**Data Filename**	HSXC935.d	**Sample Name**	HSXC935
**Sample Type**	Sample	**Position**	P1-A1
**Instrument Name**	Instrument 1	**User Name**	
**Acq Method**	s.m	**Acquired Time**	5/22/2019 4:07:39 PM
**IRM Calibration Status**	Success	**DA Method**	Default.m
**Comment**			

Sample Group **Info.**
Acquisition SW 6200 series TOF/6500 series
Version Q-TOF B.05.01 (B5125.2)

User Spectra

Fragmentor Voltage	Collision Energy	Ionization Mode
135	0	ESI

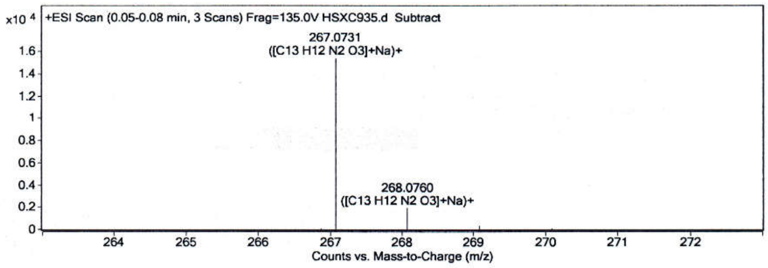

Peak List

m/z	z	Abund	Formula	Ion
107.9662		792.06		
150.1114	1	2084.28		
245.0912	1	12831.15		
246.0938	1	1645.69		
267.0731	1	15404.96	C13 H12 N2 O3	(M+Na)+
268.076	1	1920.1	C13 H12 N2 O3	(M+Na)+
283.0475	1	3709.39		
284.0508	1	627.77		
352.052		812.39		
511.1589	1	1498.89		

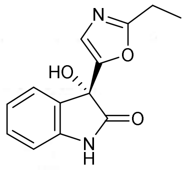

Formula Calculator Element Limits

Element	Min	Max
C	3	120
H	0	240
O	0	30
N	0	15

Formula Calculator Results

Formula	CalculatedMass	CalculatedMz	Mz	Diff. (mDa)	Diff. (ppm)	DBE
C13 H12 N2 O3	244.0848	267.0740	267.0731	0.90	3.37	9.0000

--- End Of Report ---

Figure S47. HRESIMS spectrum of compound **6**.

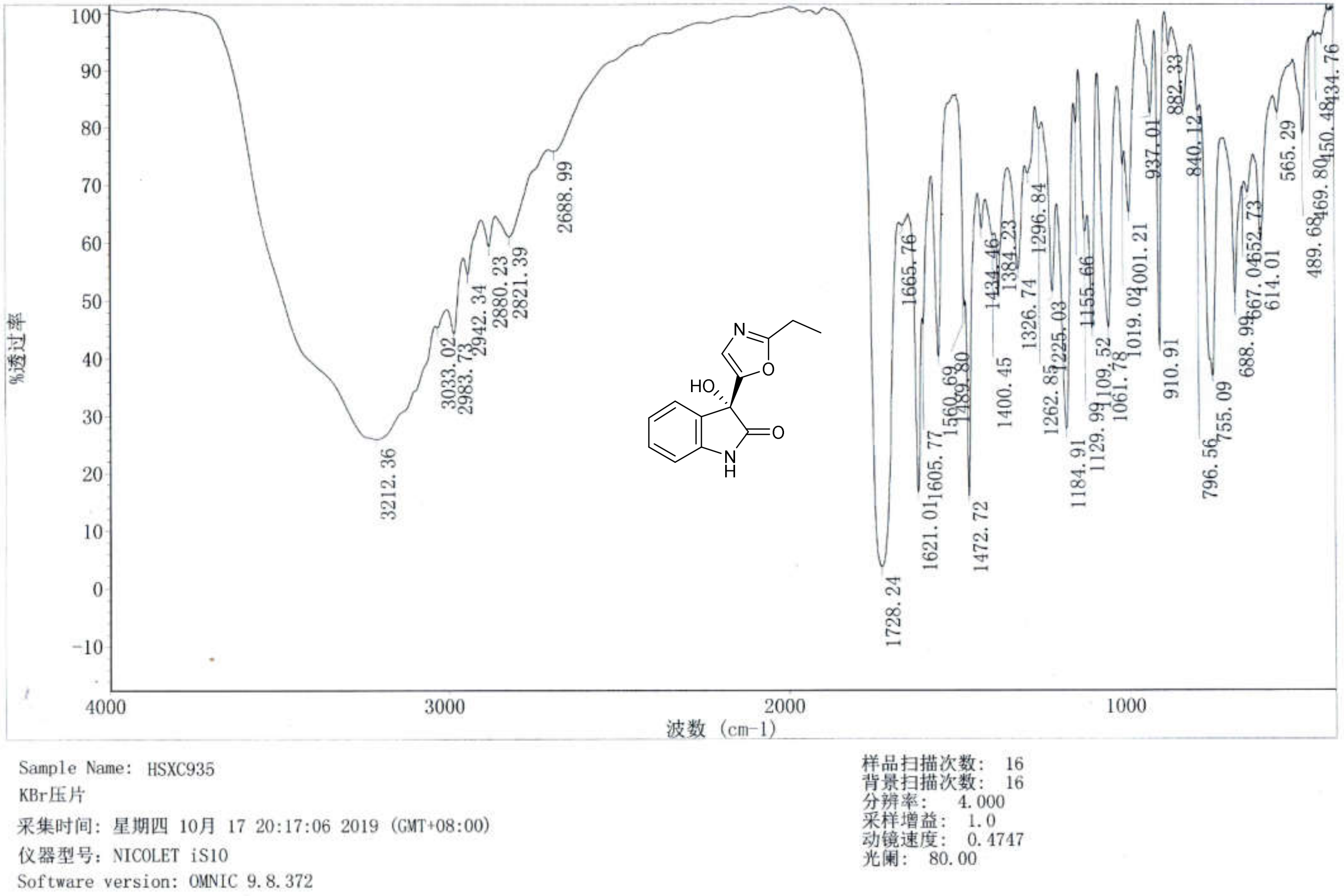

Figure S48. IR spectrum of compound **6**.

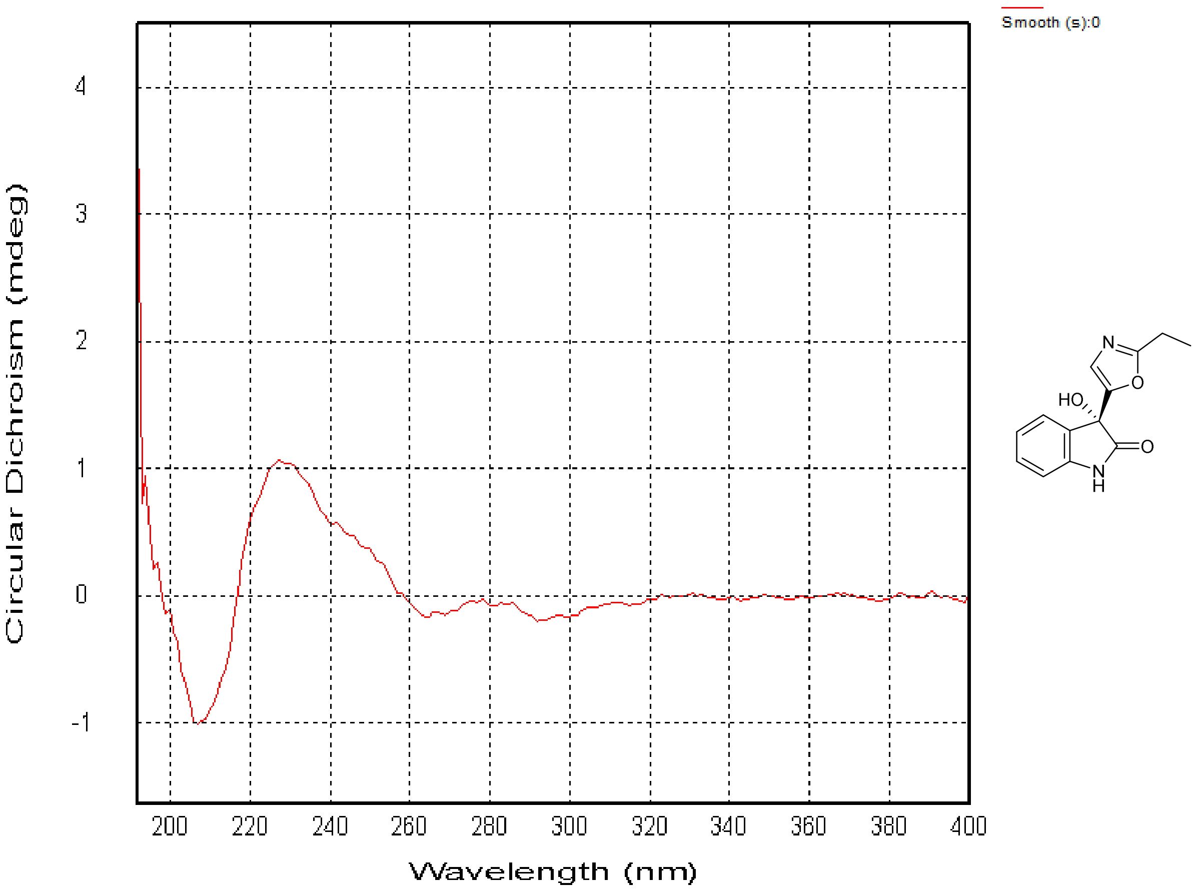

Figure S49. CD spectrum of compound **6**.
